# Synthesis of Azacarbolines via PhIO_2_-Promoted Intramolecular Oxidative Cyclization of α-Indolylhydrazones

**DOI:** 10.1021/acs.joc.1c02217

**Published:** 2021-12-06

**Authors:** Matteo Corrieri, Lucia De Crescentini, Fabio Mantellini, Giacomo Mari, Stefania Santeusanio, Gianfranco Favi

**Affiliations:** Department of Biomolecular Sciences, Section of Chemistry and Pharmaceutical Technologies, University of Urbino “Carlo Bo”, Via I Maggetti 24, 61029 Urbino, Italy

## Abstract

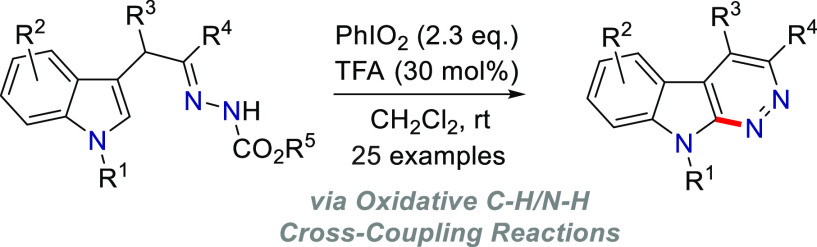

An unprecedented synthesis of polysubstituted indole-fused pyridazines (azacarbolines) from α-indolylhydrazones under oxidative conditions using a combination of iodylbenzene (PhIO_2_) and trifluoroacetic acid (TFA) has been developed. This transformation is conducted without the need for transition metals, harsh conditions, or an inert atmosphere.

## Introduction

**Figure 1:**
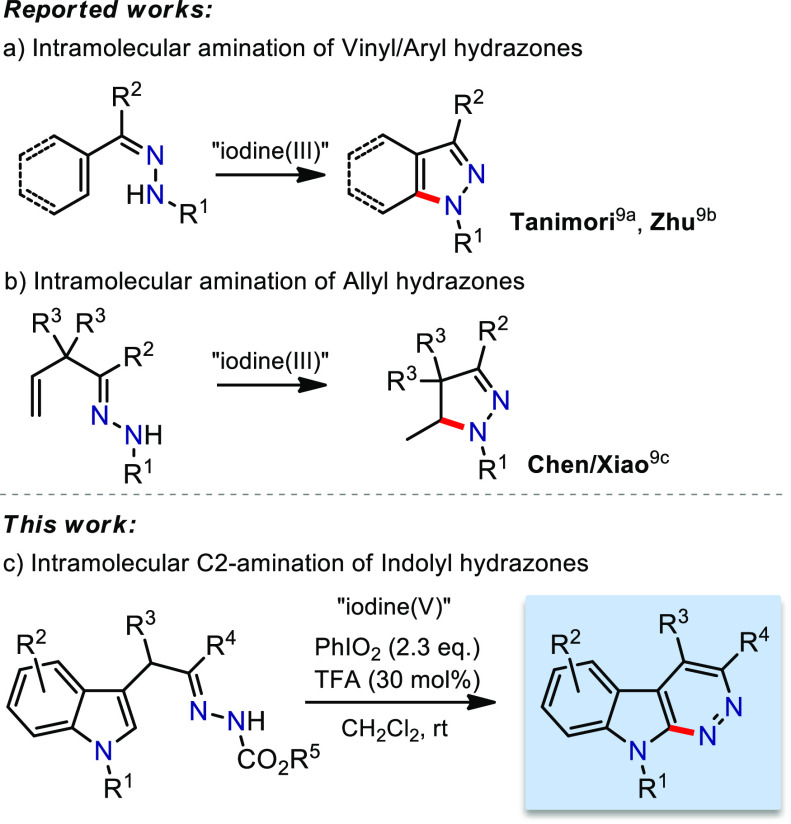
Hypervalent iodine-promoted C(sp^2^)–H cycloamination of hydrazones.

Selective carbon–nitrogen (C–N) bond formation is one of the most important processes in organic chemistry since it enables key steps in the synthesis of complex nitrogen-containing compounds from simple precursors.^1^ Traditionally, methods for C–N bond construction were routinely focused on copper-catalyzed Ullmann–Goldberg,^2^ Chan–Lam,^3^ and Pd-catalyzed Buchwald–Hartwig^4^ aminations using (pseudo)halocarbon or organometallic reagents. The recent maturation of methodologies (photochemical included) operating via transition-metal [Pd, Rh, Ru, Cu, etc.] catalyzed direct C–H bond amination^5^ without prefunctionalization of simple starting materials offers a valuable alternative. However, these reactions generally suffer from high reaction temperature, narrow substrate scope, and high loading of the catalyst and/or metal oxidant. In addition, the contamination of heavy metals in the final product has limited their potential application in drug synthesis in the later stages. Hence, the development of alternative, effective, and safe metal-free methods for the formation of C–N bonds that can be performed at milder conditions starting from nonprefunctionalized simple precursor bonds is highly desirable. In this context, hypervalent iodine reagents^6^ (HIRs) have captured our attention because of their inherent low toxicity, ready accessibility, low cost, high chemoselectivity, and mild conditions. Despite substantial advances in the oxidative C–H amination/amidation aiming at a greener goal,^7,8^ to the best of our knowledge, the application of HIRs in the C(sp^2^)–H/N–H dehydrogenative coupling annulation reactions of hydrazone systems^9^ to assemble N-heterocycles, especially those fused, still remains limited. Specifically, Tanimori’s^9a^ and Zhu’s^9b^ groups independently reported the synthesis of structurally diversified pyrazole/1*H*-indazole derivatives through metal-free oxidative C(sp^2^)–H cycloamination of both vinyl and aryl hydrazones (Figure 1a). Almost simultaneously, Chen, Xiao, and coauthors^9c^ disclosed a PhI(OAc)_2_-promoted radical cyclization of allyl hydrazones for the assembly of a wide range of five-membered dihydropyrazoles (Figure 1b). Although an excellent example describing a copper-catalyzed intramolecular C–N bond formation to afford cinnolines has been reported by Xiao, Xu, and co-workers,^10^ a metal-free approach to access a fused six-membered pyridazine skeleton from hydrazone substrates is yet to be realized.

Following our interest in the construction of polycyclic N-heterocycles^11^ and aware of the privileged role of the indole nucleus in natural products and medicinal science,^12,13^ we envisaged that the NH moiety in α-(indol-3-yl)hydrazones can be used as a N donor in coupling with the indole C2–H^7a,7i,14^ bond in the presence of the hypervalent iodine reagents (HIRs) to construct fused indole pyridazines (Figure 1c).

Herein, we report an unprecedented example of PhIO_2_-promoted dehydrogenative cyclization of α-indolylhydrazones **1**, whereby a sequential C–N bond formation, aromatization, N–C bond cleavage of a carbamate residue, ring expansion, and oxidative process are involved. Notably, this approach has resulted in a convenient assemblage of two biologically important heterocycles such as indole and pyridazine frameworks. The fusion of these two privileged heterocycles in one molecule^15^ may create rigid entities endowed with either enhanced (synergistic effect) or new biological activities, which may feature promising bioactivity for screening. Furthermore, compared with Xiao and Xu’s protocol,^10^ this method offers the clear advantage of not requiring the use of transition metal catalysts and harsh reaction conditions.

## Results and Discussion

Generation of the required substrates **1** is readily achieved in 23–95% yields by ZnCl_2_-catalyzed reaction of the indoles with azoalkenes in CH_2_Cl_2_^16^ (see Supporting Information). The intramolecular cyclization of α-indolylhydrazone **1a** was initially investigated by applying Reddy’s conditions.^7e^ To our satisfaction, the combination of PIDA with TFA (30 mol %) in CH_2_Cl_2_ at room temperature for 0.5 h afforded the product **2a** in 56% yield (Table 1, entry 1). Conducting the reaction at 0 °C instead of ambient temperature resulted in a slower and lower conversion (entry 2). The replacement of TFA by diphenyl phosphoric acid (DPP) under identical reaction conditions also decreased the yield of **2a** (entry 3). Additional variations of the initial conditions, including the use of I_2_ or Cu(OTf)_2_ as a promoter, led to poorer results (entries 4 and 5). It was also found that basic additives such as DBU and K_2_CO_3_ had a detrimental effect, as lower yields were achieved (entries 6 and 7). Whereas Cu(OTf)_2_ was crucial as an additive in previously reported iodine(III)-promoted oxidative C(sp^2^)–H cycloamination,^18^ here it showed lower efficiency (entry 8). Though a more rapid consumption of α-indolylhydrazone **1a** was observed with the use of a stoichiometric amount of TFA, the reaction only furnished 41% yield of the desired product **2a** (entry 9). Solvents like CHCl_3_, CH_3_OH, CH_3_CN, and THF (entries 10–13) were substantially less efficient in terms of the product yield. While replacing PIDA with PIFA, HTIB (Koser’s reagent), or PhIO failed to furnish better results (entries 14–16), at the switching of PIDA to other hypervalent iodine(V) oxidants^19^ such as IBX, DMP, and iodylbenzene (PhIO_2_), we were pleased to witness higher yields of **1a** into **2a** (entries 17–19). In particular, when PhIO_2_ as an uncommon iodine(V) reagent (λ^5^-iodane) was applied, the yield was improved to 82% (entry 19). A brief re-examination of the solvents still identified CH_2_Cl_2_ as optimal (entries 19–24). No improvement in yield was attained when the reaction was performed at 50 °C in DCE (entry 20) or when reducing the amount of PhIO_2_ to 1.5 equiv (entry 25).

**Table 1:**
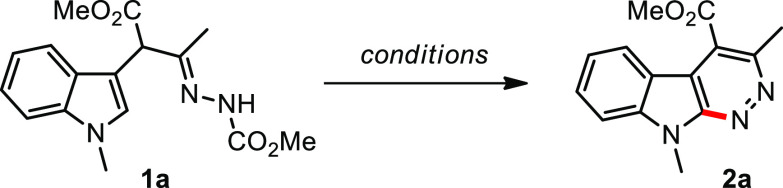
Optimization Studies^a^

entry	oxidant (equiv)	additive (equiv)	solvent (2 mL)	time (h)^b^	yield (%)^c^
1	PIDA (2.3)	TFA (0.3)	CH_2_Cl_2_	0.5	56
2^d^	PIDA (2.3)	TFA (0.3)	CH_2_Cl_2_	4	43
3	PIDA (2.3)	DPP (0.3)	CH_2_Cl_2_	3	44
4	PIDA (2.3)	I_2_ (1.5)	CH_2_Cl_2_	1	<5
5	PIDA (2.3)	Cu(OTf)_2_ (0.1)	CH_2_Cl_2_	>24	17
6	PIDA (2.3)	DBU (1.2)	CH_2_Cl_2_	12	25
7	PIDA (2.3)	K_2_CO_3_ (1.2)	CH_2_Cl_2_	12	35^e^
8^f^	PIDA (2.3)	TFA (0.3)	CH_2_Cl_2_	0.5	51
9	PIDA (2.3)	TFA (1.0)	CH_2_Cl_2_	0.2	41
10	PIDA (2.3)	TFA (0.3)	CHCl_3_	0.5	55
11	PIDA (2.3)	TFA (0.3)	CH_3_OH	0.5	35
12	PIDA (2.3)	TFA (0.3)	CH_3_CN	0.5	40
13	PIDA (2.3)	TFA (0.3)	THF	1	43
14	PIFA (2.3)	TFA (0.3)	CH_2_Cl_2_	0.3	46
15	HTIB (2.3)	TFA (0.3)	CH_2_Cl_2_	5	<5
16	PhIO (2.3)	TFA (0.3)	CH_2_Cl_2_	3	37
17	IBX (2.3)	TFA (0.3)	CH_2_Cl_2_	4	79
18	DMP (2.3)	TFA (0.3)	CH_2_Cl_2_	12	64
19	PhIO_2_ (2.3)	TFA (0.3)	CH_2_Cl_2_	5	82
20^g^	PhIO_2_ (2.3)	TFA (0.3)	DCE	2.5	70
21	PhIO_2_ (2.3)	TFA (0.3)	THF	6	68
22	PhIO_2_ (2.3)	TFA (0.3)	CH_3_CN	6	65 (16)^h^
23	PhIO_2_ (2.3)	TFA (0.3)	HFIP	3	38
24	PhIO_2_ (2.3)	–	AcOH	1	47
25	PhIO_2_ (1.5)	TFA (0.3)	CH_2_Cl_2_	12	73 (9)^h^
26	–	TFA (0.3→1)	CH_2_Cl_2_	24^i^	0
27	PhIO_2_ (2.3)	–	CH_2_Cl_2_	24^i^	0 (5)^h^

a All reactions were performed on a 0.2 mmol scale.

b Denotes complete consumption of **1a** unless otherwise noted.

c Isolated yields.

d Performed at 0 °C.

e 1-Methyl-1*H*-indole-2,3-dione^17^ (12% yield) byproduct was also recovered.

f Cu(OTf)_2_ (5 mol %) was added.

g Performed at 50 °C.

h Five-membered cross-coupled product **C** was also observed.

i Denotes unreacted starting material. Abbreviations used: PIDA = phenyliodine diacetate, PIFA = phenyliodine bis(trifluoroacetate), HTIB = hydroxy(tosyloxy)iodobenzene, IBX = *o*-iodoxybenzoic acid [1-hydroxy-1,2-benziodoxol-3(1*H*)-one-1-oxide], DMP = Dess–Martin periodinate, DPP = diphenyl phosphoric acid, TFA = trifluoroacetic acid, AcOH = acetic acid, DBU = 1,8-diazabicyclo[5.4.0]undec-7-ene, DCE = 1,2-dichloroethane, THF = tetrahydrofuran, HFIP = hexafluoroisopropanol.
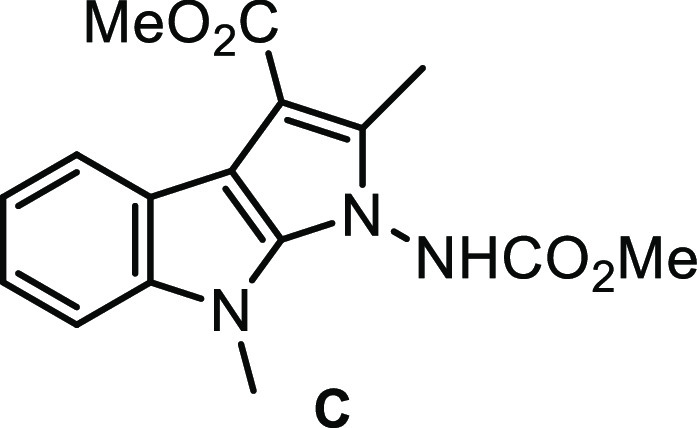

Control experiments also revealed that no product formation **2a** was detected in the absence of PhIO_2_ (entry 26) or TFA (entry 27). This indicated that both PhIO_2_ and TFA were essential for the reaction to proceed smoothly. Therefore, the optimal reaction conditions can be summarized as follows: 0.2 mmol of substrate in CH_2_Cl_2_ (2 mL) with PhIO_2_ oxidant (2.3 equiv) and TFA additive (30 mol %) at room temperature for 5 h (Table 1).

With the optimal conditions in hand, the substrate scope and the limitations of the oxidative intramolecular C–H amination with PhIO_2_ were investigated (Table 2). An array of α-indolylhydrazones **1a**–**y** were explored, resulting in the expected azacarbolines **2a**–**y** in good to excellent yields. As shown in Table 2, various substituents on the azacarboline skeleton were accommodated. Although the ester (R^3^ = CO_2_Me, CO_2_Et, CO_2_*i-*Pr, CO_2_*t-*But, and CO_2_Allyl) or phosphonate (R^3^ = PO(OMe)_2_) groups in substrates **1** were well supported, the tolerance of amide (R^3^ = CON(Me)_2_) as well as the phenyl (R^3^ = Ph) group was lower. It was pleasing to find that incorporation of a bisindole moiety into the substrate proved a success, furnishing intriguing polyazaheterocyclic architecture **2j**. The reaction conditions were also suitable for substrates bearing R^4^ alkyl groups, such as methyl, ethyl, and an *n*-propyl or ethyl acetate appendage. Various functional groups at the 4-, 5-, 6-, or 7-positions of the indole ring, regardless of electron-donating (Me, MeO, BnO) (**2q**–**2s**) and electron-withdrawing (Cl, Br, F, CO_2_Me) ones (**2t**–**2x**), were compatible with the optimized conditions. Furthermore, indole substrates with *N*-methyl, *N*-propyl, and *N*-benzyl (R^1^ = Me, *n*-Pr, Bn) substituents gave good yields of cyclized products. In contrast, the NH-free indole **1p** proceeded with poor conversion (21% yield), probably due to its attenuate intrinsic reactivity. Pleasantly, azacarboline **2y** incorporating a ring system between the N and C7 atoms of the indole ring was also prepared in good yield. It is important to note that this transformation allowed the installation of plural functionalities that are potentially well suited for future synthetic manipulations (for example, metal-catalyzed cross-coupling reactions, etc.). Interestingly, azacarboline with phosphorus substitution (**2h**) could serve as novel pharmaceuticals and agrochemicals.^20^

**Table 2:**
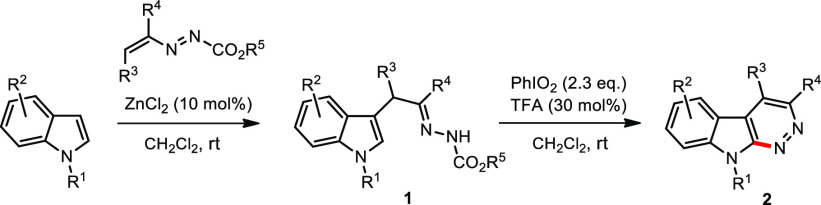

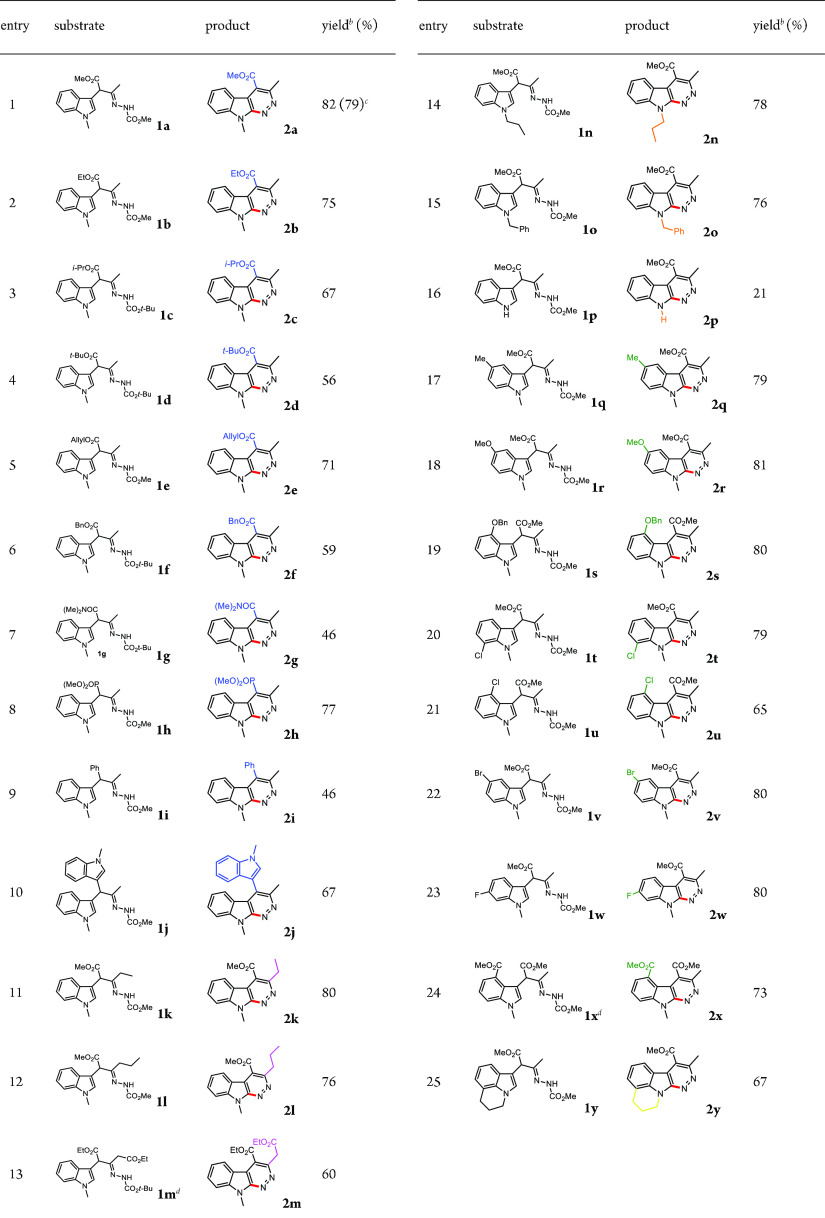
Synthesis of Azacarbolines via Intramolecular Oxidative Indole C–H Amination Mediated by PhIO_2_^a^

a Reactions were conducted on a 0.2 mmol scale in 2.0 mL of solvent.

b Isolated yields.

c 3.0 mmol scale reaction (0.605 g).

d Hydrazine tautomeric form.

The cycloamination reaction of **1a** was also conducted on a 3 mmol scale, thus demonstrating the scalability of the present method (79% yield).

**Scheme 1:**
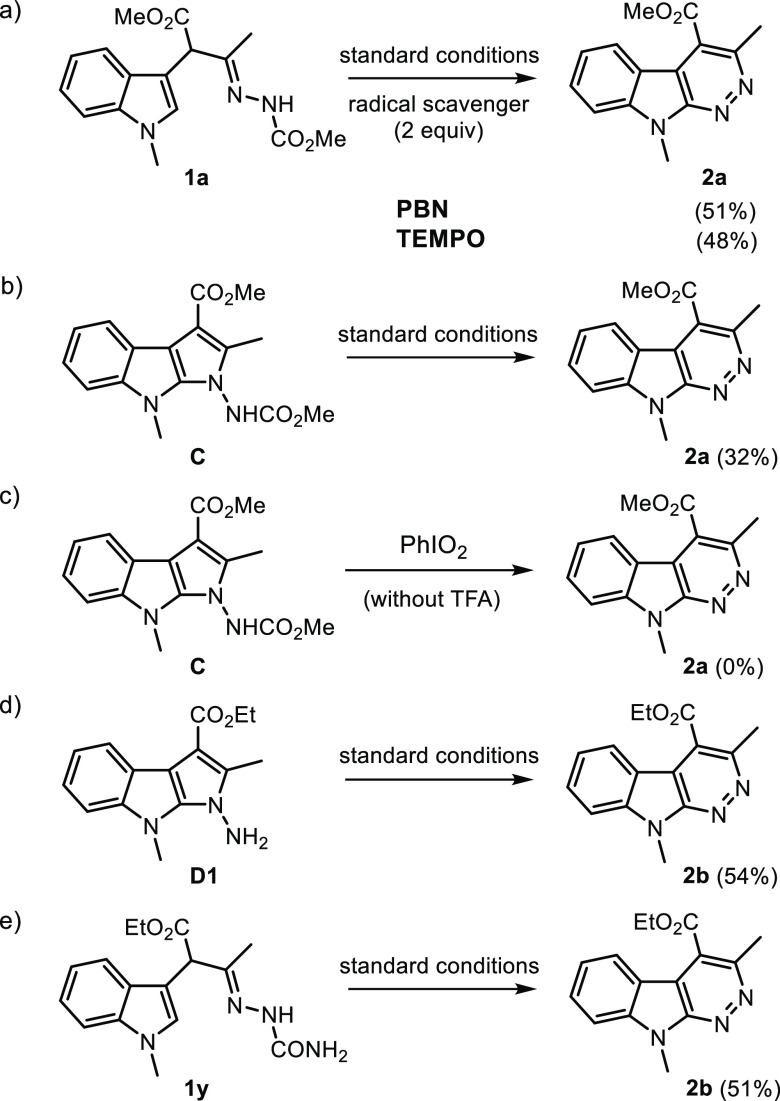
Control Experiments PBN = *N-tert*-butyl-α-phenylnitrone; TEMPO = (2,2,6,6-tetramethylpiperidin-1-yl)oxyl).

To gain insight into the reaction mechanism, we carried out further control experiments (Scheme 1).

First, the application of *N*-*tert*-butyl-α-phenylnitrone (PBN)^21^ or (2,2,6,6-tetramethylpiperidin-1-yl)oxyl (TEMPO)^9a^ as a radical scavenger evidenced that the transformation of **1a** to **2a** was not suppressed (51% and 48% yield, respectively, Scheme 1a). This fact suggests that radical intermediates were not involved in this process. Second, the treatment of isolated five-membered cross-coupled product **C** (entries 22, 25, and 27, Table 1) under the reaction conditions was found to give product **2a** (Scheme 1b), the result of which indicated its effective involvement in the reaction mechanism. The preliminary formation of a less polar spot which gradually disappeared in favor of the final product **2a** (TLC monitoring) also confirmed that **C** was the productive intermediate for this transformation. On the other hand, the same intermediate **C** did not work when subjected with PhIO_2_ alone (Scheme 1c). Third, when the prepared hydrolyzed pyrrolo[2,3-*b*]indole **D1** was subjected under standard conditions, the expected **2b** was successfully obtained (Scheme 1d). Lastly, substrate **1z** with an amide N-protective group (CONH_2_) also furnished the corresponding azacarboline **2b** in good yields (Scheme 1e).

**Scheme 2:**
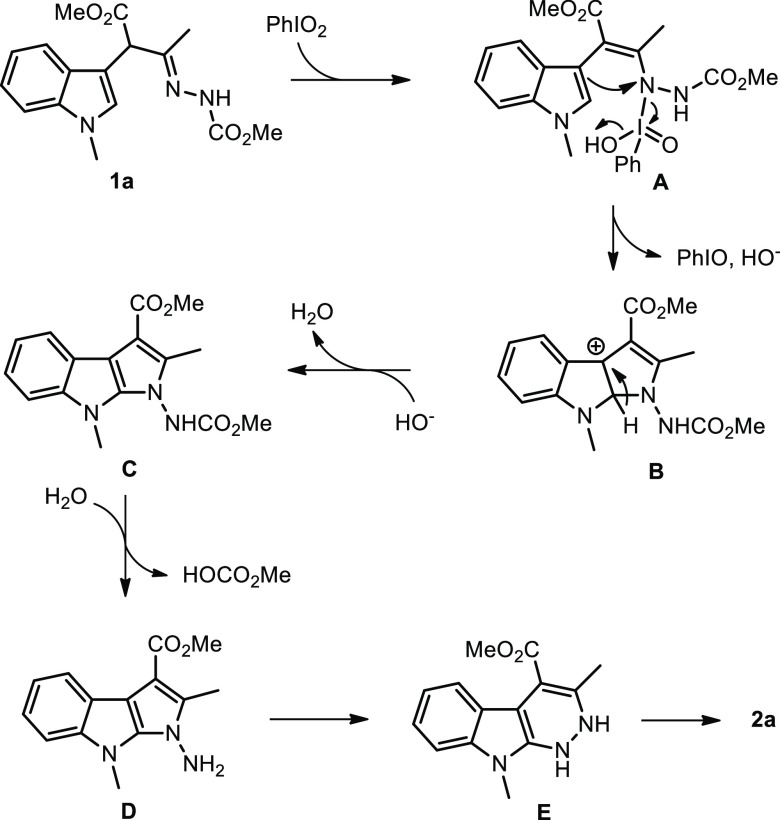
Tentative Mechanism for the Oxidative C–H Amination

Based on these results and in agreement with the previous references, a tentative mechanism for the oxidative C–H amination of α-indolylhydrazones is presented in Scheme 2.

Initially, PhIO_2_ reacts with **1a** to give an *N*-iodo intermediate **A** after a CH/NH tautomerization (1,3-H shift). The subsequent electrophilic cyclization (oxidative C–N bond formation) step takes place between C-2 of the indole and nitrogen activated by the electrophilic iodine species generating intermediate **B** with simultaneous loss of PhIO and HO^–^. This was then followed by the formation of key pyrrolo[2,3-*b*]indole intermediate **C** through successive deprotonation and aromatization. Finally, the hydrolysis of a carbamic residue (intermediate **D**), ring expansion reaction,^22^ and oxidative aromatization from **E** afford the desired azacarboline **2a**. The explanation for the role of TFA is not immediately intuited, but its beneficial effect is clearly demonstrated in these latter steps (see Scheme 1b, 1c, and 1d).^22^ However, considering that the transformation of intermediate **C** into **2a** under standard conditions is not straightforward (32% yield, Scheme 1b), an alternative reaction pathway resulting from six-membered electrophilic cyclization may also be operative. In this case, the oxidative C–N bond formation would occur at the other nitrogen atom of the hydrazone residue, which could afford the final product **2a** after undergoing the hydrolysis and oxidative aromatization steps.^23^

**Scheme 3:**
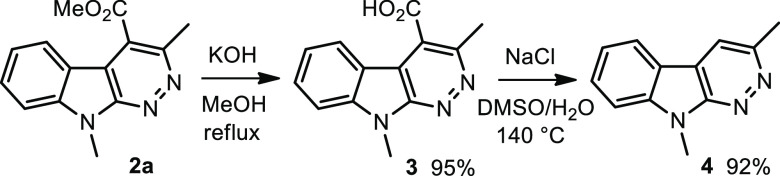
Transformation of Generated Azacarbolines

To further demonstrate the potential and synthetic usefulness of this method, the generated azacarbolines were transformed as shown in Scheme 3. The ester group at the 4-position of **2a** could be easily hydrolyzed by treatment with KOH in methanol at reflux.^24^ Decarboxylation was possible from **3** by heating at 140 °C in the presence of NaCl in DMSO/H_2_O.^25^

## Conclusion

In conclusion, we have developed a practical, environmentally friendly, and metal-free methodology for intramolecular oxidative cyclization of α-indolylhydrazones at room temperature. Complementary with existing methods, this approach allows direct access to scarcely represented azacarbolines^14^ via dehydrogenative C(sp^2^)–N bond formation using the less emblazoned PhIO_2_^26,27^ hypervalent iodine(V) reagent. We believe that obtaining of such fused N-heterocyclic scaffolds that incorporate both the privileged indole and pyridazine core with the aid of a “forgotten” PhIO_2_ through the not easy oxidative C–H/N–H cross coupling could open the way for further interesting novel applications.

## Experimental Section

### General Experimental Details

All the commercially available reagents and solvents were used without further purification. The following compounds were synthesized according to literature procedures: HTIB,^28^ PhIO,^29^ PhIO_2_,^30^ IBX,^31^ and DMP.^32^*CAUTION!* PhIO, PhIO_2_, and IBX are explosive under impact or heating to >200 °C, and appropriate precautions should be taken while handling these products. However, we have not experienced any explosions while working with these compounds at room temperature.

α-(Indol-3-yl)hydrazones **1a**–**i**,**k**–**z** were prepared according to our previously reported methods^16a,16b^ with a slight modification. Bis(indolyl)methane hydrazone **1j** was prepared following literature procedure.^16c^ Chromatographic purification of compounds was carried out on silica gel (60–200 μm). TLC analysis was performed on preloaded (0.25 mm) glass-supported silica gel plates (Kieselgel 60); compounds were visualized by exposure to UV light and by dipping the plates in 1% Ce(SO_4_)·4H_2_O and 2.5% (NH_4_)_6_Mo_7_O_24_·4H_2_O in 10% sulfuric acid followed by heating on a hot plate. All ^1^H NMR and ^13^C NMR spectra were recorded at 400 and 100 MHz using DMSO-*d*_6_ or CDCl_3_ as solvent on a Bruker Ultrashield 400 spectrometer (Bruker, Billerica, MA, USA). Chemical shifts (δ scale) are reported in parts per million (ppm) relative to the central peak of the solvent and are sorted in descending order within each group. The following abbreviations are used to describe peak patterns where appropriate: s = singlet, d = doublet, dd = doublet of doublets, dt = doublet of triplets, td = triplet of doublets, t = triplet, q = quartet, sex = sextet, sept = septet, m = multiplet, and br = broad signal. All coupling constants (*J* value) are given in Hertz [Hz]. High-resolution mass spectroscopy was performed on a Micromass Q-TOF Micro mass spectrometer (Micromass, Manchester, UK) using an ESI source. Melting points were determined in open capillary tubes and are uncorrected.

### General Procedure for the Preparation of α-(Indol-3-yl)hydrazones **1a**–**i**,**k**–**z**^16a,16b^

To a stirred mixture of indole (1.0 mmol) and azoalkene (1.5 mmol, 1.5 equiv) in dichloromethane (4 mL), zinc dichloride (13.6 mg, 0.1 mmol, 10 mol %) was added. (In order to obtain compound **1p**, the addition of DIPEA (174 μL, 1 mmol, 1 equiv) was required.) After the disappearance of indole (TLC check), the solvent was removed, and the crude mixture was purified by column chromatography on silica gel to afford, after crystallization, the α-(indol-3-yl)hydrazones **1**.

### Procedure for the Preparation of Bis(indolyl)Methane Hydrazone **1j**(16c)

1-Methylindole (0.75 mL, 6 mmol, 4 equiv) was added to a previously stirred solution of Na_2_CO_3_ (1.59 g, 15 mmol, 10 equiv) in water (5 mL). The dichloroacetone hydrazone (298.5 mg, 1.5 mmol) in dichloromethane (5 mL) was added, and the reaction mixture was stirred at room temperature. Upon completion of the reaction (1 h, TLC check), the mixture was diluted with water (10 mL) and extracted with dichloromethane (3 × 20 mL), and the collected organic phases were dried over anhydrous Na_2_SO_4_. After filtration, the reaction was concentrated *in vacuo*, and the obtained crude was purified by flash chromatography to afford the bis(indolyl)methane hydrazone **1j**.

The NMR spectra in DMSO-*d*_6_ showed that compounds **1** exist predominantly in the hydrazone structure; however, signals related to the hydrazine tautomeric form can be also observed.

### Methyl 2-(4-Methoxy-3-(1-methyl-1*H*-indol-3-yl)-4-oxobutan-2-lidene)hydrazinecarboxylate

Compound **1a** was isolated by column chromatography (ethyl acetate/cyclohexane 50:50) in 68% yield (216.8 mg) for 1 h; white solid; mp 122–124 °C. ^1^H NMR (400 MHz, DMSO-*d*_6_) δ 9.88 (s, 1H), 7.47–7.43 (m, 1H), 7.43–7.39 (m, 1H), 7.32 (s, 1H), 7.19–7.13 (m, 1H), 7.04–7.00 (m, 1H), 4.87 (s, 1H), 3.80 (s, 3H), 3.68 (s, 6H), 1.79 (s, 3H); ^13^C{^1^H} NMR (100 MHz, DMSO-*d*_6_) δ 171.1, 154.5, 151.0, 136.5, 128.5, 126.8, 121.3, 119.0, 118.7, 109.8, 107.5, 51.9, 51.7, 51.3, 32.4, 14.4; HRMS (ESI/Q-TOF) *m*/*z* [M + H]^+^ Calcd for C_16_H_20_N_3_O_4_ 318.1448; Found 318.1445.

### Methyl 2-(4-Ethoxy-3-(1-methyl-1*H*-indol-3-yl)-4-oxobutan-2-ylidene)hydrazinecarboxylate

Compound **1b** was isolated by column chromatography (ethyl acetate/cyclohexane 50:50) in 89% yield (294.0 mg) for 0.5 h; white solid; mp 119–121 °C. ^1^H NMR (400 MHz, DMSO-*d*_6_) δ 9.90 (s, 1H), 7.47–7.45 (m, 1H), 7.42–7.40 (m, 1H), 7.32 (s, 1H), 7.18–7.14 (m, 1H), 7.04–7.00 (m, 1H), 4.84 (s, 1H), 4.20–4.12 (m, 2H), 3.77 (s, 3H), 3.68 (s, 3H), 1.21 (t, *J* = 7.2 Hz, 3H), 1.79 (s, 3H); ^13^C{^1^H} NMR (100 MHz, DMSO-*d*_6_) δ 170.6, 154.6, 151.1, 136.5, 128.4, 126.8, 121.3, 119.0, 118.7, 109.8, 107.6, 60.6, 51.8, 51.4, 32.4, 14.4, 14.0; HRMS (ESI/Q-TOF) *m*/*z* [M + H]^+^ Calcd for C_17_H_22_N_3_O_4_ 332.1605; Found 332.1611.

### *tert-*Butyl 2-(4-Isopropoxy-3-(1-methyl-1*H*-indol-3-yl)-4-oxobutan-2-ylidene)hydrazinecarboxylate

Compound **1c** was isolated by column chromatography (ethyl acetate/cyclohexane 20:80) in 84% yield (325.7 mg) for 1 h; white solid; mp 108–110 °C. ^1^H NMR (400 MHz, DMSO-*d*_6_) δ 9.51 (s, 1H), 7.46 (d, *J* = 8.0 Hz, 1H), 7.43–7.39 (m, 1H), 7.30 (s, 1H), 7.17–7.12 (m, 1H), 7.05–7.00 (m, 1H), 4.98 (sept, *J* = 6.4 Hz, 1H), 4.76 (d, *J* = 0.4 Hz, 1H), 3.77 (s, 3H), 1.76 (s, 3H), 1.45 (s, 9H), 1.21 (t, *J* = 6.4 Hz, 6H); ^13^C{^1^H} NMR (100 MHz, DMSO-*d*_6_) δ 170.1, 153.1, 150.3, 136.5, 128.3, 126.9, 121.3, 118.9, 118.8, 109.8, 107.8, 73.1, 68.0, 51.6, 32.4, 28.1, 21.5, 14.4; HRMS (ESI/Q-TOF) *m*/*z* [M + H]^+^ Calcd for C_21_H_30_N_3_O_4_ 388.2231; Found 388.2226.

### *tert-*Butyl 2-(4-(*tert*-Butoxy)-3-(1-methyl-1*H*-indol-3-yl)-4-oxobutan-2-ylidene)hydrazinecarboxylate

Compound **1d** was isolated by column chromatography (ethyl acetate/cyclohexane 20:80) in 95% yield (380.1 mg) for 7 h; orange solid; mp 91–93 °C. ^1^H NMR (400 MHz, DMSO-*d*_6_) δ 9.48 (s, 1H), 7.47 (d, *J* = 8.0 Hz, 1H), 7.40 (d, *J* = 8.4 Hz, 1H), 7.28 (s, 1H), 7.17–7.12 (m, 1H), 7.04–7.00 (m, 1H), 4.68 (s, 1H), 3.77 (s, 3H), 1.76 (s, 3H), 1.46 (s, 9H), 1.44 (s, 9H); ^13^C{^1^H} NMR (100 MHz, DMSO-*d*_6_) δ 170.3, 153.6, 151.1, 137.0, 128.6, 127.4, 121.8, 119.4, 119.3, 110.2, 108.6, 81.2, 79.6, 52.9, 32.9, 28.6, 28.2, 14.8; HRMS (ESI/Q-TOF) *m*/*z* [M + H]^+^ Calcd for C_22_H_32_N_3_O_4_ 402.2387; Found 402.2400.

### Methyl 2-(4-(Allyloxy)-3-(1-methyl-1*H*-indol-3-yl)-4-oxobutan-2-ylidene)hydrazinecarboxylate

Compound **1e** was isolated by column chromatography (ethyl acetate/cyclohexane 50:50) in 59% yield (203.9 mg) for 3 h; orange solid; mp 178–180 °C. ^1^H NMR (400 MHz, DMSO-*d*_6_) δ 9.91 (s, 1H), 7.46 (dt, *J* = 8.0, 0.8 Hz, 1H), 7.41 (dt, *J* = 8.0, 0.8 Hz, 1H), 7.33 (s, 1H), 7.16 (td, *J* = 8.0, 0.8 Hz, 1H), 7.02 (td, *J* = 8.0, 0.8 Hz, 1H), 5.99–5.89 (m, 1H), 5.32–5.27 (m, 1H), 5.21–5.18 (m, 1H), 4.90 (s, 1H), 4.65–4.62 (m, 2H), 3.77 (s, 3H), 3.68 (s, 3H), 1.79 (s, 3H); ^13^C{^1^H} NMR (100 MHz, DMSO-*d*_6_) δ 170.8, 155.1, 151.4, 137.0, 133.0, 129.0, 127.3, 121.8, 119.5, 119.3, 118.4, 110.3, 107.9, 65.5, 52.3, 51.8, 32.9, 15.0; HRMS (ESI/Q-TOF) *m*/*z* [M + H]^+^ Calcd for C_18_H_22_N_3_O_4_ 344.1605; Found 344.1621.

### *tert-*Butyl 2-(4-(Benzyloxy)-3-(1-methyl-1*H*-indol-3-yl)-4-oxobutan-2-ylidene)hydrazinecarboxylate

Compound **1f** was isolated by column chromatography (ethyl acetate/cyclohexane 20:80) in 75% yield (325.6 mg) for 3 h; white solid; mp 118–120 °C. ^1^H NMR (400 MHz, DMSO-*d*_6_) δ 9.59 (s, 1H), 7.48 (d, *J* = 8.0 Hz, 1H), 7.41–7.30 (m, 7H), 7.17–7.11 (m, 1H), 7.00 (t, *J* = 7.4 Hz, 1H), 5.21 (d, *J* = 12.4 Hz, 1H), 5.15 (d, *J* = 12.4 Hz, 1H), 4.91 (s, 1H), 3.75 (s, 3H), 1.78 (s, 3H), 1.47 (s, 9H); ^13^C{^1^H} NMR (100 MHz, DMSO-*d*_6_) δ 170.6, 163.6, 153.0, 136.5, 136.0, 128.6, 128.3, 128.1, 128.0, 127.9, 126.9, 121.3, 118.9, 109.7, 107.6, 79.1, 66.0, 51.4, 32.4, 28.1, 14.6; HRMS (ESI/Q-TOF) *m*/*z* [M + H]^+^ Calcd for C_25_H_30_N_3_O_4_ 436.2231; Found 436.2234.

### *tert-*Butyl 2-(4-(Dimethylamino)-3-(1-methyl-1*H*-indol-3-yl)-4-oxobutan-2-ylidene)hydrazinecarboxylate

Compound **1g** was isolated by column chromatography (ethyl acetate/cyclohexane 30:70) in 82% yield (303.8 mg) for 24 h; white solid; mp 108–110 °C. ^1^H NMR (400 MHz, DMSO-*d*_6_) δ 9.42 (s, 1H), 7.45 (d, *J* = 8.0 Hz, 1H), 7.40 (d, *J* = 8.0 Hz, 1H), 7.24 (s, 1H), 7.17–7.13 (m, 1H), 7.03–7.00 (m, 1H), 5.02 (s, 1H), 3.76 (s, 3H), 2.88 (s, 3H), 2.87 (s, 3H), 1.73 (s, 3H), 1.45 (s, 9H); ^13^C{^1^H} NMR (100 MHz, DMSO-*d*_6_) δ 170.4, 153.1, 136.6, 128.4, 126.8, 121.3, 118.9, 118.5, 109.8, 108.2, 79.0, 48.9, 37.0, 35.1, 32.4, 28.1, 14.9; HRMS (ESI/Q-TOF) *m*/*z* [M + H]^+^ Calcd for C_20_H_29_N_4_O_3_ 373.2234; Found 373.2238.

### Methyl 2-(1-(Dimethoxyphosphoryl)-1-(1-methyl-1*H*-indol-3-yl)propan-2-ylidene)hydrazinecarboxylate

Compound **1h** was isolated by column chromatography (ethyl acetate/methanol 95:5) in 69% yield (261.9 mg) for 18 h; orange solid; mp 159–161 °C. ^1^H NMR (400 MHz, DMSO-*d*_6_) δ 9.92 (br, 1H), 7.57 (d, *J* = 8.0 Hz, 1H), 7.48 (d, *J* = 1.6 Hz, 1H), 7.41 (d, *J* = 8.0 Hz, 1H), 7.18–7.14 (m, 1H), 7.06–7.02 (m, 1H), 4.55 (d, ^2^*J*_HP_ = 24.0 Hz, 1H), 3.79 (s, 3H), 3.68 (s, 3H), 3.67 (d, ^3^*J*_HP_ = 10.4 Hz, 3H), 3.60 (d, ^3^*J*_HP_ = 10.4 Hz, 3H), 1.85 (d, ^4^*J*_HP_ = 1.2 Hz, 3H); ^13^C{^1^H} NMR (100 MHz, DMSO-*d*_6_) δ 154.7, 150.0, 136.3, 129.0 (d, ^3^*J*_CP_ = 5.6 Hz), 127.3 (d, ^2^*J*_CP_ = 10.5 Hz), 121.5, 119.0, 118.7, 109.8, 105.0 (d, ^3^*J*_CP_ = 6.5 Hz), 53.0 (d, ^2^*J*_CP_ = 6.8 Hz), 52.9 (d, ^2^*J*_CP_ = 6.8 Hz), 51.9, 43.7 (d, ^1^*J*_CP_ = 138.0 Hz), 32.5, 15.0 (d, ^3^*J*_CP_ = 3.0 Hz); HRMS (ESI/Q-TOF) *m*/*z* [M + H]^+^ Calcd for C_17_H_23_N_3_O_5_P 368.1370; Found 368.1368.

### Methyl 2-(1-(1-Methyl-1*H*-indol-3-yl)-1-phenylpropan-2-ylidene)hydrazinecarboxylate

Compound **1i** was isolated by column chromatography (ethyl acetate/cyclohexane 30:70) in 73% yield (244.2 mg) for 1 h; white solid; mp 179–181 °C. ^1^H NMR (400 MHz, DMSO-*d*_6_) δ 9.79 (s, 1H), 7.42–7.38 (m, 1H), 7.35–7.20 (m, 6H), 7.16–7.11 (m, 2H), 6.95 (t, *J* = 7.4 Hz, 1H), 5.19 (s, 1H), 3.75 (s, 3H), 3.66 (s, 3H), 1.85 (s, 3H); ^13^C{^1^H} NMR (100 MHz, DMSO-*d*_6_) δ 155.2, 141.5, 137.2, 128.9, 128.9, 128.7, 128.6, 127.5, 127.0, 121.7, 119.4, 119.1, 113.6, 110.1, 52.2, 51.4, 32.8, 15.8; HRMS (ESI/Q-TOF) *m*/*z* [M + H]^+^ Calcd for C_20_H_22_N_3_O_2_ 336.1707; Found 336.1717.

### Methyl 2-(1,1-Bis(1-methyl-1*H*-indol-3-yl)propan-2-ylidene)hydrazinecarboxylate

Compound **1j** was isolated by column chromatography (ethyl acetate/cyclohexane 40:60) in 24% yield (142.0 mg) for 1 h; white solid; mp 188–190 °C. ^1^H NMR (400 MHz, DMSO-*d*_6_) δ 9.74 (s, 1H), 7.45 (d, *J* = 8.0 Hz, 2H), 7.40 (d, *J* = 8.4 Hz, 2H), 7.16 (s, 2H), 7.16–7.12 (m, 2H), 7.00–6.96 (m, 2H), 5.40 (s, 1H), 3.74 (s, 6H), 3.67 (s, 3H), 1.83 (s, 3H); ^13^C{^1^H} NMR (100 MHz, DMSO-*d*_6_) δ 155.6, 154.7, 136.7, 127.9, 127.1, 121.1, 119.0, 118.5, 113.1, 109.6, 51.7, 42.8, 32.3, 14.3; HRMS (ESI/Q-TOF) *m*/*z* [M + H]^+^ Calcd for C_23_H_25_N_4_O_2_ 389.1972; Found 389.1979.

### Methyl 2-(1-Methoxy-2-(1-methyl-1*H*-indol-3-yl)-1-oxopentan-3-ylidene)hydrazinecarboxylate

Compound **1k** was isolated by column chromatography (ethyl acetate/cyclohexane 40:60) in 80% yield (264.1 mg) for 1 h; white solid; mp 124–126 °C. ^1^H NMR (400 MHz, DMSO-*d*_6_) δ 10.00 (s, 1H), 7.51–7.49 (m, 1H), 7.41–7.39 (m, 1H), 7.33 (s, 1H), 7.17–7.13 (m, 1H), 7.03–7.00 (m, 1H), 4.88 (s, 1H), 3.77 (s, 3H), 3.68 (s, 3H), 3.65 (s, 3H), 2.45–2.35 (m, 1H), 2.21–2.12 (m, 1H), 0.74 (t, *J* = 7.6 Hz, 3H). ^13^C{^1^H} NMR (100 MHz, DMSO-*d*_6_) δ 171.2, 154.5, 154.5, 136.5, 128.8, 127.0, 121.3, 119.0, 119.0, 109.7, 107.6, 51.8, 51.8, 49.9, 32.4, 21.0, 9.7; HRMS (ESI/Q-TOF) *m*/*z* [M + H]^+^ Calcd for C_17_H_22_N_3_O_4_ 332.1605; Found 332.1598.

### Methyl 2-(1-Methoxy-2-(1-methyl-1*H*-indol-3-yl)-1-oxohexan-3-ylidene)hydrazinecarboxylate

Compound **1l** was isolated by column chromatography (ethyl acetate/cyclohexane 40:60) in 73% yield (251.8 mg) for 2 h; white solid; mp 124–126 °C. ^1^H NMR (400 MHz, DMSO-*d*_6_) δ 10.04 (s, 1H), 7.51–7.49 (m, 1H), 7.41–7.39 (m, 1H), 7.33 (s, 1H), 7.16–7.12 (m, 1H), 7.03–6.99 (m, 1H), 4.86 (s, 1H), 3.76 (s, 3H), 3.68 (s, 3H), 3.64 (s, 3H), 2.42–2.35 (m, 1H), 2.13–2.06 (m, 1H), 1.31–1.07 (m, 2H), 0.74 (t, *J* = 7.4 Hz, 3H); ^13^C{^1^H} NMR (100 MHz, DMSO-*d*_6_) δ 171.1, 154.4, 153.4, 136.5, 128.8, 127.0, 121.2, 119.0, 118.8, 109.6, 107.6, 51.7, 51.7, 50.0, 32.3, 29.7, 18.2, 13.7. HRMS (ESI/Q-TOF) *m*/*z* [M + H]^+^ Calcd for C_18_H_24_N_3_O_4_ 346.1761; Found 346.1752.

### Diethyl 3-(2-(*tert*-Butoxycarbonyl)hydrazono)-2-(1-methyl-1*H*-indol-3-yl)pentanedioate

Compound **1m** was isolated as a hydrazine tautomeric form by column chromatography (ethyl acetate/cyclohexane 40:60) in 75% yield (333.1 mg) for 3 h; white solid; mp 150–152 °C. ^1^H NMR (400 MHz, DMSO-*d*_6_) δ 10.18 (s, 1H), 9.08 (br, 1H), 7.38 (d, *J* = 8.4 Hz, 1H), 7.24 (d, *J* = 7.6 Hz, 1H), 7.12 (t, *J* = 7.4 Hz, 1H), 7.03 (s, 1H), 6.98 (t, *J* = 7.2 Hz, 1H), 4.00–3.92 (m, 4H), 3.75 (s, 3H), 3.12 (s, 2H), 1.41 (s, 9H), 1.11 (t, *J* = 7.2 Hz, 3H), 1.00 (t, *J* = 7.0 Hz, 3H); ^13^C{^1^H} NMR (100 MHz, DMSO-*d*_6_) δ 170.1, 168.7, 159.0, 156.9, 136.7, 130.1, 129.0, 121.3, 119.7, 119.0, 110.2, 110.0, 80.2, 60.8, 59.2, 35.9, 32.8, 28.5, 28.4, 14.8, 14.3; HRMS (ESI/Q-TOF) *m*/*z* [M + H]^+^ Calcd for C_23_H_32_N_3_O_6_ 446.2286; Found 446.2298.

### Methyl 2-(4-Methoxy-4-*oxo*-3-(1-propyl-1*H*-indol-3-yl)butan-2-ylidene)hydrazinecarboxylate

Compound **1n** was isolated by column chromatography (ethyl acetate/cyclohexane 40:60) in 70% yield (242.8 mg) for 1 h; white solid; mp 116–118 °C. ^1^H NMR (400 MHz, DMSO-*d*_6_) δ 9.89 (s, 1H), 7.44 (t, *J* = 8.0 Hz, 2H), 7.36 (s, 1H), 7.15–7.11 (m, 1H), 7.02–6.99 (m, 1H), 4.86 (s, 1H), 4.14–4.10 (m, 2H), 3.68 (s, 3H), 3.67 (s, 3H), 1.76 (s, 3H), 1.75 (sex, *J* = 7.2 Hz, 2H), 0.82 (t, *J* = 7.2 Hz, 3H); ^13^C{^1^H} NMR (100 MHz, DMSO-*d*_6_) δ 171.1, 154.6, 151.0, 135.8, 127.6, 126.9, 121.3, 118.9, 118.8, 110.0, 107.5, 51.9, 51.8, 51.3, 47.0, 23.1, 14.3, 11.1; HRMS (ESI/Q-TOF) *m*/*z* [M + H]^+^ Calcd for C_18_H_24_N_3_O_4_ 346.1761; Found 346.1767.

### Methyl 2-(3-(1-Benzyl-1*H*-indol-3-yl)-4-methoxy-4-oxobutan-2-ylidene)hydrazinecarboxylate

Compound **1o** was isolated by column chromatography (ethyl acetate/cyclohexane 50:50) in 44% yield (147.1 mg) for 3 h; white solid; mp 128–130 °C. ^1^H NMR (400 MHz, DMSO-*d*_6_) δ 9.93 (s, 1H), 7.53 (s, 1H), 7.46 (d, *J* = 8.0 Hz, 1H), 7.42 (d, *J* = 8.4 Hz, 1H), 7.32–7.28 (m, 2H), 7.25–7.23 (m, 1H), 7.21–7.17 (m, 2H), 7.12–7.08 (m, 1H), 7.03–6.99 (m, 1H), 5.42 (s, 2H), 4.91 (s, 1H), 3.68 (s, 3H), 3.68 (s, 3H), 1.79 (s, 3H); ^13^C{^1^H} NMR (100 MHz, DMSO-*d*_6_) δ 171.1, 154.6, 150.9, 138.1, 135.9, 128.5, 128.1, 127.3, 127.1, 126.9, 121.5, 119.2, 118.9, 110.3, 108.2, 52.0, 51.8, 51.3, 49.0, 14.4; HRMS (ESI/Q-TOF) *m*/*z* [M + H]^+^ Calcd for C_22_H_24_N_3_O_4_ 394.1761; Found 394.1768.

### Methyl 2-(3-(1*H*-Indol-3-yl)-4-methoxy-4-oxobutan-2-ylidene)hydrazinecarboxylate

Compound **1p** was isolated by column chromatography (ethyl acetate/cyclohexane 30:70) in 23% yield (69.0 mg) for 6 h; whitish solid; mp 112–114 °C. ^1^H NMR (400 MHz, DMSO-*d*_6_) δ 11.11 (s, 1H), 9.90 (s, 1H), 7.42 (d, *J* = 8.0 Hz, 1H), 7.37 (d, *J* = 8.0 Hz, 1H), 7.31 (d, *J* = 2.4 Hz, 1H), 7.10–7.06 (m, 1H), 7.00–6.96 (m, 1H), 4.86 (s, 1H), 3.67 (s, 6H), 1.77 (s, 3H); ^13^C{^1^H} NMR (100 MHz, DMSO-*d*_6_) δ 171.3, 154.6, 151.2, 136.1, 126.5, 124.3, 121.3, 118.9, 118.5, 111.6, 108.3, 51.9, 51.8, 51.4, 14.4; HRMS (ESI/Q-TOF) *m*/*z* [M + H]^+^ Calcd for C_15_H_18_N_3_O_4_ 304.1292; Found 318.1297.

### Methyl 2-(3-(1,5-Dimethyl-1*H*-indol-3-yl)-4-methoxy-4-oxobutan-2-ylidene)hydrazinecarboxylate

Compound **1q** was isolated by column chromatography (ethyl acetate/cyclohexane 40:60) in 76% yield (252.5 mg) for 0.25 h; white solid; mp 120–122 °C. ^1^H NMR (400 MHz, DMSO-*d*_6_) δ 9.90 (s, 1H), 7.29 (d, *J* = 8.4 Hz, 1H), 7.25 (s, 1H), 7.24–7.23 (m, 1H), 6.97 (dd, *J* = 8.4, 1.6 Hz, 1H), 4.82 (s, 1H), 3.73 (s, 3H), 3.67 (s, 3H), 3.67 (s, 3H), 2.36 (s, 3H), 1.79 (s, 3H); ^13^C{^1^H} NMR (100 MHz, DMSO-*d*_6_) δ 171.2, 154.6, 151.2, 135.0, 128.5, 127.5, 127.0, 123.0, 118.2, 109.6, 106.9, 52.0, 51.8, 51.2, 32.4, 21.3, 14.5; HRMS (ESI/Q-TOF) *m*/*z* [M + H]^+^ Calcd for C_17_H_22_N_3_O_4_ 332.1605; Found 332.1593.

### Methyl 2-(4-Methoxy-3-(5-methoxy-1-methyl-1*H*-indol-3-yl)-4-oxobutan-2-ylidene)hydrazinecarboxylate

Compound **1r** was isolated by column chromatography (ethyl acetate/cyclohexane 40:60) in 62% yield (215.1 mg) for 0.5 h; white solid; mp 108–110 °C. ^1^H NMR (400 MHz, DMSO-*d*_6_) δ 9.92 (s, 1H), 7.30 (d, *J* = 9.2 Hz, 1H), 7.28 (s, 1H), 6.97 (d, *J* = 2.4 Hz, 1H), 6.80 (dd, *J* = 9.2, 2.4 Hz, 1H), 4.84 (s, 1H), 3.73 (s, 3H), 3.72 (s, 3H), 3.68 (s, 3H), 3.67 (s, 3H), 1.77 (s, 3H); ^13^C{^1^H} NMR (100 MHz, DMSO-*d*_6_) δ 117.2, 154.6, 153.4, 151.1, 131.8, 128.9, 127.2, 111.3, 110.6, 106.9, 100.8, 55.2, 52.0, 51.8, 51.3, 32.6, 14.4; HRMS (ESI/Q-TOF) *m*/*z* [M + H]^+^ Calcd for C_17_H_22_N_3_O_5_ 348.1554; Found 348.1543.

### Methyl 2-(3-(4-(Benzyloxy)-1-methyl-1*H*-indol-3-yl)-4-methoxy-4-oxobutan-2-ylidene)hydrazinecarboxylate

Compound **1s** was isolated by column chromatography (ethyl acetate/cyclohexane 50:50) in 32% yield (133.6 mg) for 1 h; white solid; mp 129–130 °C. ^1^H NMR (400 MHz, DMSO-*d*_6_) δ 9.87 (s, 1H), 7.50–7.48 (m, 2H), 7.40–7.37 (m, 2H), 7.33–7.29 (m, 1H), 7.05–6.98 (m, 3H), 6.59 (d, *J* = 7.2 Hz, 1H), 5.22 (s, 1H), 5.19 (d, *J* = 12.4 Hz, 1H), 5.13 (d, *J* = 12.4 Hz, 1H), 3.72 (s, 3H), 3.64 (s, 3H), 3.48 (s, 3H), 1.88 (s, 3H); ^13^C{^1^H} NMR (100 MHz, DMSO-*d*_6_) δ 171.4, 154.5, 152.7, 151.1, 138.0, 137.2, 128.3, 127.5, 127.4, 126.9, 122.2, 116.9, 108.2, 103.2, 100.8, 69.1, 51.9, 51.7, 51.6, 32.6, 15.5; HRMS (ESI/Q-TOF) *m*/*z* [M + H]^+^ Calcd for C_23_H_26_N_3_O_5_ 424.1867; Found 424.1872.

### Methyl 2-(3-(7-Chloro-1-methyl-1*H*-indol-3-yl)-4-methoxy-4-oxobutan-2-ylidene)hydrazinecarboxylate

Compound **1t** was isolated by column chromatography (ethyl acetate/cyclohexane 50:50) in 50% yield (174.6 mg) for 3 h; white solid; mp 130–132 °C. ^1^H NMR (400 MHz, DMSO-*d*_6_) δ 9.94 (s, 1H), 7.42 (d, *J* = 8.0 Hz, 1H), 7.39 (s, 1H), 7.14 (d, *J* = 7.6 Hz, 1H), 7.00–6.96 (m, 1H), 4.88 (s, 1H), 4.08 (s, 3H), 3.67 (s, 3H), 3.67 (s, 3H), 1.78 (s, 3H); ^13^C{^1^H} NMR (100 MHz, DMSO-*d*_6_) δ 170.9, 154.6, 150.6, 131.7, 131.4, 130.2, 122.8, 120.1, 118.2, 116.0, 108.0, 52.1, 51.8, 51.0, 36.2, 14.5; HRMS (ESI/Q-TOF) *m*/*z* [M + H]^+^ Calcd for C_16_H_19_ClN_3_O_4_ 352.1059; Found 352.1054.

### Methyl 2-(3-(4-Chloro-1-methyl-1*H*-indol-3-yl)-4-methoxy-4-oxobutan-2-ylidene)hydrazinecarboxylate

Compound **1u** was isolated by column chromatography (ethyl acetate/cyclohexane 50:50) in 32% yield (113.6 mg) for 2 h; white solid; mp 148–150 °C. ^1^H NMR (400 MHz, DMSO-*d*_6_) δ 9.95 (s, 1H), 7.42 (dd, *J* = 8.0, 0.8 Hz, 1H), 7.22 (s, 1H), 7.16–7.12 (m, 1H), 7.05 (dd, *J* = 7.6 Hz, 0.8 Hz, 1H), 5.31 (s, 1H), 3.78 (s, 3H), 3.65 (s, 3H), 3.64 (s, 3H), 1.92 (s, 3H). Interconversion to the hydrazine tautomeric form occurred during the carbon spectrum acquisition, and as a result, two distinct sets of signals of both hydrazone and hydrazine tautomers (ca. 50:50) were observed in DMSO-*d*_6_ solution at 20 °C. ^13^C{^1^H} NMR (100 MHz, DMSO-*d*_6_) δ 171.3, 170.0, 162.2, 156.9, 154.5, 138.0, 137.7, 131.2, 130.1, 125.1, 124.7, 124.3, 123.4, 122.1, 121.6, 119.9, 119.4, 110.0, 109.4, 108.9, 108.1, 88.8, 52.2, 52.0, 51.8, 51.5, 50.4, 32.8, 32.6, 15.9, 15.9; HRMS (ESI/Q-TOF) *m*/*z* [M + H]^+^ Calcd for C_16_H_19_ClN_3_O_4_ 352.1059; Found 352.1051.

### Methyl 2-(3-(5-Bromo-1-methyl-1*H*-indol-3-yl)-4-methoxy-4-oxobutan-2-ylidene)hydrazinecarboxylate

Compound **1v** was isolated by column chromatography (ethyl acetate/cyclohexane 50:50) in 39% yield (156.3 mg) for 1 h; white solid; mp 155–157 °C. ^1^H NMR (400 MHz, DMSO-*d*_6_) δ 9.94 (s, 1H), 7.64 (d, *J* = 1.6 Hz, 1H), 7.42–7.40 (m, 2H), 7.26 (dd, *J* = 8.8, 2.0 Hz, 1H), 4.89 (s, 1H), 3.77 (s, 3H), 3.67 (s, 3H), 3.67 (s, 3H), 1.78 (s, 3H); ^13^C{^1^H} NMR (100 MHz, DMSO-*d*_6_) δ 170.9, 154.6, 150.8, 130.3, 130.2, 128.6, 123.8, 121.3, 112.0, 111.7, 107.3, 52.0, 51.8, 51.0, 32.6, 14.7; HRMS (ESI/Q-TOF) *m*/*z* [M + H]^+^ Calcd for C_16_H_19_BrN_3_O_4_ 396.0553; Found 396.0545.

### Methyl 2-(3-(6-Fluoro-1-methyl-1*H*-indol-3-yl)-4-methoxy-4-oxobutan-2-ylidene)hydrazinecarboxylate

Compound **1w** was isolated by column chromatography (ethyl acetate/cyclohexane 50:50) in 56% yield (187.2 mg) for 1 h; white solid; mp 140–142 °C. ^1^H NMR (400 MHz, DMSO-*d*_6_) δ 9.91 (s, 1H), 7.42 (dd, *J* = 8.8 Hz, ^4^*J*_HF_ = 5.6 Hz, 1H), 7.33 (s, 1H), 7.29 (dd, ^3^*J*_HF_ = 10.4 Hz, *J* = 2.4 Hz, 1H), 6.91–6.86 (m, 1H), 4.86 (s, 1H), 3.73 (s, 3H), 3.67 (s, 3H), 3.34 (s, 3H), 1.77 (s, 3H); ^13^C{^1^H} NMR (100 MHz, DMSO-*d*_6_) δ 171.0, 159.0 (d, ^1^*J*_CF_ = 233.6 Hz), 154.5, 150.9, 136.6 (d, ^3^*J*_CF_ = 12.3 Hz), 129.1 (d, ^4^*J*_CF_ = 3.3 Hz), 123.5, 120.0 (d, ^3^*J*_CF_ = 10.2 Hz), 107.9, 107.4 (d, ^2^*J*_CF_ = 24.4 Hz), 96.2 (d, ^2^*J*_CF_ = 25.9 Hz), 52.0, 51.8, 51.2, 32.6, 14.5; HRMS (ESI/Q-TOF) *m*/*z* [M + H]^+^ Calcd for C_16_H_19_FN_3_O_4_ 336.1354; Found 336.1358.

### Methyl 3-(1-Methoxy-3-(2-(methoxycarbonyl)hydrazono)-1-oxobutan-2-yl)-1-methyl-1*H*-indole-4-carboxylate

Compound **1x** was isolated as the hydrazine tautomeric form by column chromatography (ethyl acetate/cyclohexane 50:50) in 64% yield (200.3 mg) for 1 h; white solid; mp 162–164 °C. ^1^H NMR (400 MHz, DMSO-*d*_6_) δ 10.22 (s, 1H), 9.45 (br, 1H), 7.62 (d, *J* = 8.4 Hz, 1H), 7.36 (d, *J* = 6.4 Hz, 1H), 7.21–7.17 (m, 2H), 3.80 (s, 3H), 3.71 (s, 3H), 3.64 (s, 3H), 3.36 (s, 3H), 1.67 (s, 3H); ^13^C{^1^H} NMR (100 MHz, DMSO-*d*_6_) δ 170.3, 169.2, 161.3, 157.4, 137.7, 133.0, 125.7, 124.8, 121.3, 120.3, 113.8, 110.8, 91.2, 52.6, 52.5, 50.6, 32.9, 16.1; HRMS (ESI/Q-TOF) *m*/*z* [M + H]^+^ Calcd for C_18_H_22_N_3_O_6_ 376.1503; Found 376.1499.

### Methyl 2-(3-(5,6-Dihydro-4*H*-pyrrolo[3,2,1-*ij*]quinolin-1-yl)-4-methoxy-4-oxobutan-2-ylidene)hydrazinecarboxylate

Compound **1y** was isolated by column chromatography (ethyl acetate/cyclohexane 50:50) in 44% yield (152.5 mg) for 1 h; white solid; mp 138–140 °C. ^1^H NMR (400 MHz, DMSO-*d*_6_) δ 9.90 (s, 1H), 7.31 (s, 1H), 7.22 (d, *J* = 8.0 Hz, 1H), 6.90 (t, *J* = 7.2 Hz, 1H), 6.94 (d, *J* = 7.2 Hz, 1H), 4.85 (s, 1H), 4.13 (t, *J* = 5.6 Hz, 2H), 3.68 (s, 3H), 3.67 (s, 3H), 2.90 (t, *J* = 6.0 Hz, 2H), 2.14–2.08 (m, 2H), 1.80 (s, 3H); ^13^C{^1^H} NMR (100 MHz, DMSO-*d*_6_) δ 171.3, 154.6, 151.3, 133.8, 125.8, 124.4, 121.9, 119.4, 118.3, 116.3, 107.5, 51.9, 51.8, 51.6, 43.4, 24.0, 22.3, 14.5; HRMS (ESI/Q-TOF) *m*/*z* [M + H]^+^ Calcd for C_18_H_22_N_3_O_4_ 344.1605; Found 344.1604.

### Ethyl 3-(2-Carbamoylhydrazono)-2-(1-methyl-1*H*-indol-3-yl)butanoate

The chemical–physical data of compound **1z** are in agreement with those previously reported.^16b^

### General Procedure for the Synthesis of Azacarbolines **2** via PhIO_2_-Mediated Intramolecular Oxidative Cyclization of α-Indolylhydrazones **1**

To a stirred mixture of α-indolylhydrazone **1** (0.2 mmol) in dichloromethane (2 mL) were added PhIO_2_ (108.6 mg, 0.46 mmol, 2.3 equiv) and TFA (5 μL, 0.06 mmol, 30 mol %). After that, the solution was stirred overnight at room temperature. The crude product was directly purified by flash chromatography on silica gel (cyclohexane/ethyl acetate) to give the corresponding product **2**.

### Methyl 3,9-Dimethyl-9*H*-pyridazino[3,4-*b*]indole-4-carboxylate

Compound **2a** was isolated by column chromatography (ethyl acetate/cyclohexane 50:50) in 82% yield (41.7 mg); yellow solid; mp 112–114 °C. ^1^H NMR (400 MHz, DMSO-*d*_6_) δ 8.12 (d, *J* = 8.0 Hz, 1H), 7.81–7.77 (m, 2H), 7.38–7.34 (m, 1H), 4.13 (s, 3H), 4.05 (s, 3H), 2.82 (s, 3H); ^13^C{^1^H} NMR (100 MHz, DMSO-*d*_6_) δ 166.6, 152.8, 146.8, 142.7, 131.2, 124.9, 121.7, 120.9, 115.9, 114.9, 110.5, 53.2, 28.1, 20.2; HRMS (ESI/Q-TOF) *m*/*z* [M + H]^+^ Calcd for C_14_H_14_N_3_O_2_ 256.1081; Found 256.1078.

### Ethyl 3,9-Dimethyl-9*H*-pyridazino[3,4-*b*]indole-4-carboxylate

Compound **2b** was isolated by column chromatography (ethyl acetate/cyclohexane 50:50) in 75% yield (40.2 mg); yellow solid; mp 127–129 °C. ^1^H NMR (400 MHz, DMSO-*d*_6_) δ 8.13 (d, *J* = 8.0 Hz, 1H), 7.79–7.77 (m, 2H), 7.38–7.33 (m, 1H), 4.61 (q, *J* = 7.2 Hz, 2H), 4.04 (s, 3H), 2.82 (s, 3H), 1.42 (t, *J* = 7.2 Hz, 3H); ^13^C{^1^H} NMR (100 MHz, DMSO-*d*_6_) δ 166.1, 152.8, 146.7, 142.6, 131.1, 124.9, 122.1, 120.8, 115.9, 114.8, 110.5, 62.4, 28.1, 20.2, 13.9; HRMS (ESI/Q-TOF) *m*/*z* [M + H]^+^ Calcd for C_15_H_16_N_3_O_2_: 270.1237; Found 270.1240.

### Isopropyl 3,9-Dimethyl-9*H*-pyridazino[3,4-*b*]indole-4-carboxylate

Compound **2c** was isolated by column chromatography (ethyl acetate/cyclohexane 50:50) in 67% yield (38.1 mg); yellow solid; mp 105–107 °C. ^1^H NMR (400 MHz, DMSO-*d*_6_) δ 8.13 (d, *J* = 8.0 Hz, 1H), 7.82–7.76 (m, 2H), 7.41–7.33 (m, 1H), 5.47 (sept, *J* = 6.4 Hz, 1 H), 4.04 (s, 3H), 2.82 (s, 3H), 1.44 (d, *J* = 6.4 Hz, 6H); ^13^C{^1^H} NMR (100 MHz, DMSO-*d*_6_) δ 165.6, 152.8, 146.4, 142.6, 131.1, 124.7, 122.5, 120.8, 115.9, 114.6, 110.6, 70.5, 28.1, 21.4, 20.0; HRMS (ESI/Q-TOF) *m*/*z* [M + H]^+^ Calcd for C_16_H_18_N_3_O_2_ 284.1394; Found 284.1390.

### *tert-*Butyl 3,9-Dimethyl-9*H*-pyridazino[3,4-*b*]indole-4-carboxylate

Compound **2d** was isolated by column chromatography (ethyl acetate/cyclohexane 40:60) in 56% yield (33.4 mg); yellow solid; mp 160–162 °C. ^1^H NMR (400 MHz, DMSO-*d*_6_) δ 8.11 (d, *J* = 8.0 Hz, 1H), 7.80–7.76 (m, 2H), 7.41–7.35 (m, 1H), 4.04 (s, 3H), 2.81 (s, 3H), 1.69 (s, 9H); ^13^C{^1^H} NMR (100 MHz, DMSO-*d*_6_) δ 165.4, 152.9, 146.2, 142.5, 131.0, 124.4, 123.4, 120.9, 115.9, 114.2, 110.6, 84.1, 28.1, 27.7, 19.9; HRMS (ESI/Q-TOF) *m*/*z* [M + H]^+^Calcd for C_17_H_20_N_3_O_2_ 298.1550; Found 298.1561.

### Allyl 3,9-Dimethyl-9*H*-pyridazino[3,4-*b*]indole-4-carboxylate

Compound **2e** was isolated by column chromatography (ethyl acetate/cyclohexane 40:60) in 71% yield (39.8 mg); yellow solid; mp 102–104 °C. ^1^H NMR (400 MHz, DMSO-*d*_6_) δ 8.15 (dt, *J* = 8.0, 0.8 Hz, 1H), 7.80–7.78 (m, 2H), 7.37–7.33 (m, 1H), 6.20–6.10 (m, 1H), 5.53–5.48 (m, 1H), 5.40–5.36 (m, 1H), 5.10 (dt, *J* = 6.0, 1.2 Hz, 2H), 4.05 (s, 3H), 2.84 (s, 3H); ^13^C{^1^H} NMR (100 MHz, DMSO-*d*_6_) δ 165.8, 152.8, 146.7, 142.7, 131.6, 131.1, 124.9, 121.7, 120.7, 119.7, 115.9, 114.8, 110.5, 66.6, 28.1, 20.1; HRMS (ESI/Q-TOF) *m*/*z*: [M + H]^+^ Calcd for C_16_H_16_N_3_O_2_ 282.1237; Found 282.1245.

### Benzyl 3,9-Dimethyl-9*H*-pyridazino[3,4-*b*]indole-4-carboxylate

Compound **2f** was isolated by column chromatography (ethyl acetate/cyclohexane 40:60) in 59% yield (39.0 mg); yellow solid; mp 132–134 °C. ^1^H NMR (400 MHz, DMSO-*d*_6_) δ 7.93 (d, *J* = 8.0 Hz, 1H), 7.78–7.41 (m, 2H), 7.47–7.38 (m, 3H), 7.58–7.54 (m, 2H), 7.24–7.19 (m, 1H), 5.64 (s, 2H), 4.03 (s, 3H), 2.79 (s, 3H); ^13^C{^1^H} NMR (100 MHz, DMSO-*d*_6_) δ 165.9, 152.8, 146.6, 142.6, 134.9, 131.1, 129.1, 128.7, 128.6, 124.9, 121.9, 120.7, 115.8, 114.8, 110.5, 67.9, 28.1, 20.1; HRMS (ESI/Q-TOF) *m*/*z* [M + H]^+^ Calcd for C_20_H_18_N_3_O_2_ 332.1394; Found 332.1387.

### *N*,*N*,3,9-Tetramethyl-9*H*-pyridazino[3,4-*b*]indole-4-carboxamide

Compound **2g** was isolated by column chromatography (ethyl acetate/cyclohexane 100:0) in 46% yield (24.7 mg); yellow solid; mp 154–156 °C. ^1^H NMR (400 MHz, DMSO-*d*_6_) δ 7.82–7.72 (m, 3H), 7.38–7.32 (m, 1H), 4.04 (s, 3H), 3.24 (s, 3H), 2.77 (s, 3H), 2.67 (s, 3H); ^13^C{^1^H} NMR (100 MHz, DMSO-*d*_6_) δ 165.6, 152.5, 145.9, 142.1, 130.6, 126.2, 123.3, 120.9, 116.3, 113.9, 110.4, 36.7, 33.9, 28.0, 18.9; HRMS (ESI/Q-TOF) *m*/*z* [M + H]^+^ Calcd for C_15_H_17_N_4_O 269.1397; Found 269.1404.

### Dimethyl (3,9-Dimethyl-9*H*-pyridazino[3,4-*b*]indol-4-yl)phosphonate

Compound **2h** was isolated by column chromatography (ethyl acetate/cyclohexane 100:0) in 77% yield (46.8 mg); yellow solid; mp 137–139 °C. ^1^H NMR (400 MHz, DMSO-*d*_6_) δ 8.93 (d, *J* = 8.0 Hz, 1H), 7.82–7.75 (m, 2H), 7.39–7.33 (m, 1H), 4.05 (s, 3H), 3.76 (s, 3H), 3.73 (s, 3H), 3.03 (d, *J* = 1.2 Hz, 3H); ^13^C{^1^H} NMR (100 MHz, DMSO-*d*_6_) δ 152.2 (d, ^2^*J*_CP_ = 11.0 Hz), 151.2 (d, ^2^*J*_CP_ = 10.2 Hz), 143.1, 131.3, 127.9, 120.6, 120.1 (d, ^3^*J*_CP_ = 8.8 Hz), 116.8, 116.7, 116.3 (d, ^1^*J*_CP_ = 178.0 Hz), 110.1, 52.7 (d, ^2^*J*_CP_ = 5.2 Hz), 28.1, 22.7; HRMS (ESI/Q-TOF) *m*/*z* [M + H]^+^ Calcd for C_14_H_17_N_3_O_3_P 306.1002; Found 306.1006.

### 3,9-Dimethyl-4-phenyl-9*H*-pyridazino[3,4-*b*]indole

Compound **2i** was isolated by column chromatography (ethyl acetate/cyclohexane 40:60) in 46% yield (24.9 mg); yellow solid; mp 166–168 °C. ^1^H NMR (400 MHz, DMSO-*d*_6_) δ 7.73–7.59 (m, 5H), 7.56–7.49 (m, 2H), 7.11–7.03 (m, 2H), 4.03 (s, 3H), 2.56 (s, 3H); ^13^C{^1^H} NMR (100 MHz, DMSO-*d*_6_) δ 152.9, 149.0, 142.1, 135.2, 132.7, 129.9, 129.2, 128.9, 128.3, 123.5, 120.1, 117.5, 116.6, 110.2, 28.0, 20.0; HRMS (ESI/Q-TOF) *m*/*z* [M + H]^+^ Calcd for C_18_H_16_N_3_ 274.1339; Found 274.1332.

### 3,9-Dimethyl-4-(1-methyl-1*H*-indol-3-yl)-9*H*-pyridazino[3,4-*b*]indole

Compound **2j** was isolated by column chromatography (ethyl acetate/cyclohexane 90:10) in 67% yield (43.7 mg); orange solid; mp 106–108 °C. ^1^H NMR (400 MHz, DMSO-*d*_6_) δ 7.81 (s, 1H), 7.69 (d, *J* = 8.0 Hz, 1H), 7.66 (d, *J* = 8.0 Hz, 1H), 7.60–7.56 (m, 1H), 7.30–7.26 (m, 1H), 7.09 (d, *J* = 8.0 Hz, 1H), 7.02–7.00 (m, 2H), 6.98–6.94 (m, 1H), 4.05 (s, 3H), 3.99 (s, 3H), 2.66 (s, 3H); ^13^C{^1^H} NMR (100 MHz, DMSO-*d*_6_) δ 152.8, 150.8, 142.0, 136.7, 129.6, 129.5, 126.6, 125.8, 124.2, 121.9, 119.8, 119.7, 119.4, 118.0, 117.6, 110.7, 109.8, 107.5, 32.9, 28.0, 20.5; HRMS (ESI/Q-TOF) *m*/*z* [M + H]^+^ Calcd for C_21_H_19_N_4_ 327.1604; Found 327.1593.

### Methyl 3-Ethyl-9-methyl-9*H*-pyridazino[3,4-*b*]indole-4-carboxylate

Compound **2k** was isolated by column chromatography (ethyl acetate/cyclohexane 50:50) in 80% yield (43.3 mg); yellow solid; mp 148–150 °C. ^1^H NMR (400 MHz, DMSO-*d*_6_) δ 8.05 (d, *J* = 8.0 Hz, 1H), 7.79–7.75 (m, 2H), 7.38–7.32 (m, 1H), 4.14 (s, 3H), 4.05 (s, 3H), 3.14 (q, *J* = 7.6 Hz, 2H), 1.34 (t, *J* = 7.6 Hz, 3H); ^13^C{^1^H} NMR (100 MHz, DMSO-*d*_6_) δ 166.6, 152.6, 151.2, 142.6, 131.0, 124.5, 121.5, 120.9, 115.9, 114.7, 110.5, 53.2, 28.1, 26.8, 14.7; HRMS (ESI/Q-TOF) *m*/*z* [M + H]^+^ Calcd for C_15_H_16_N_3_O_2_ 270.1237; Found 270.1254.

### Methyl 9-Methyl-3-propyl-9*H*-pyridazino[3,4-*b*]indole-4-carboxylate

Compound **2l** was isolated by column chromatography (ethyl acetate/cyclohexane 40:60) in 76% yield (43.1 mg); yellow oil. ^1^H NMR (400 MHz, DMSO-*d*_6_) δ 8.04 (d, *J* = 8.0 Hz, 1 H), 7.81–7.74 (m, 2 H), 7.38–7.32 (m, 1H), 4.13 (s, 3H), 4.05 (s, 3H), 3.10 (t, *J* = 7.2 Hz, 2H), 1.76 (sex, *J* = 7.2 Hz, 2H), 0.94 (t, *J* = 7.2 Hz, 3H); ^13^C{^1^H} NMR (100 MHz, DMSO-*d*_6_) δ 166.7, 152.6, 150.1, 142.5, 131.1, 124.5, 121.9, 120.9, 115.9, 114.7, 110.6, 53.3, 35.2, 28.1, 23.2, 13.7; HRMS (ESI/Q-TOF) *m*/*z* [M + H]^+^ Calcd for C_16_H_18_N_3_O_2_ 284.1394; Found 284.1408.

### Ethyl 3-(2-Ethoxy-2-oxoethyl)-9-methyl-9*H*-pyridazino[3,4-*b*]indole-4-carboxylate

Compound **2m** was isolated by column chromatography (ethyl acetate/cyclohexane 50:50) in 60% yield (41.3 mg); yellow solid; mp 102–104 °C. ^1^H NMR (400 MHz, DMSO-*d*_6_) δ 8.31 (dt, *J* = 8.0, 0.8 Hz, 1H), 7.81–7.77 (m, 2H), 7.39–7.33 (m, 1H), 4.54 (q, *J* = 7.2 Hz, 2H), 4.38 (s, 2H), 4.11 (q, *J* = 7.2 Hz, 2H), 4.05 (s, 3H), 1.39 (t, *J* = 7.2 Hz, 3H), 1.19 (t, *J* = 7.2 Hz, 3H); ^13^C{^1^H} NMR (100 MHz, DMSO-*d*_6_) δ 170.3, 165.5, 153.3, 145.1, 142.9, 131.4, 126.0, 122.2, 120.9, 116.2, 115.7, 110.5, 62.4, 60.7, 28.2, 14.0, 13.7; HRMS (ESI/Q-TOF) *m*/*z* [M + H]^+^ Calcd for C_18_H_20_N_3_O_4_ 342.1448; Found 342.1439.

### Methyl 3-Methyl-9-propyl-9*H*-pyridazino[3,4-*b*]indole-4-carboxylate

Compound **2n** was isolated by column chromatography (ethyl acetate/cyclohexane 40:60) in 78% yield (44.2 mg); yellow solid; mp 144–146 °C. ^1^H NMR (400 MHz, DMSO-*d*_6_) δ 8.11 (d, *J* = 8.0 Hz, 1H), 7.83 (d, *J* = 8.0 Hz, 1H), 7.76 (dt, *J* = 7.2, 1.2 Hz, 1H), 7.35 (dt, *J* = 7.2, 1.2 Hz, 1H), 4.58 (t, *J* = 7.6 Hz, 2H), 4.13 (s, 3H), 2.82 (s, 3H), 1.86 (sex, *J* = 7.6 Hz, 2H), 0.88 (t, *J* = 7.6 Hz, 3H); ^13^C{^1^H} NMR (100 MHz, DMSO-*d*_6_) δ 166.5, 152.6, 146.7, 142.1, 131.1, 124.9, 121.8, 120.8, 115.9, 114.7, 110.7, 53.1, 43.0, 21.4, 20.1, 11.1; HRMS (ESI/Q-TOF) *m*/*z* [M + H]^+^ Calcd for C_16_H_18_N_3_O_2_ 284.1394; Found 284.1399.

### Methyl 9-Benzyl-3-methyl-9*H*-pyridazino[3,4-*b*]indole-4-carboxylate

Compound **2o** was isolated by column chromatography (ethyl acetate/cyclohexane 40:60) in 76% yield (50.5 mg); yellow solid; mp 132–134 °C. ^1^H NMR (400 MHz, DMSO-*d*_6_) δ 8.13 (d, *J* = 8.0 Hz, 1H), 7.78 (d, *J* = 8.0 Hz, 1H), 7.73 (dt, *J* = 8.0, 1.2 Hz, 1H), 7.35 (dt, *J* = 8.0, 1.2 Hz, 1H), 7.31–7.21 (m, 5H), 5.87 (s, 2H), 4.13 (s, 3H), 2.83 (s, 3H); ^13^C{^1^H} NMR (100 MHz, DMSO-*d*_6_) δ 166.4, 152.7, 147.4, 141.9, 136.9, 131.3, 128.6, 127.5, 127.1, 125.1, 121.9, 121.2, 116.2, 115.1, 110.9, 53.3, 44.7, 20.2; HRMS (ESI/Q-TOF) *m*/*z* [M + H]^+^ Calcd for C_20_H_18_N_3_O_2_ 332.1394; Found 332.1387.

### Methyl 3-Methyl-9*H*-pyridazino[3,4-*b*]indole-4-carboxylate

Compound **2p** was isolated by column chromatography (ethyl acetate/cyclohexane 30:70) in 21% yield (10.0 mg); yellow solid; mp 200–202 °C. ^1^H NMR (400 MHz, DMSO-*d*_6_) δ 12.53 (br, 1H), 8.09 (dt, *J* = 8.4, 0.8 Hz, 1H), 7.72–7.68 (m, 1H), 7.62–7.60 (m, 1H), 7.33–7.29 (m, 1H), 4.13 (s, 3H), 2.81 (s, 3H); ^13^C{^1^H} NMR (100 MHz, DMSO-*d*_6_) δ 166.7, 154.0, 146.6, 142.0, 131.1, 124.9, 121.7, 120.6, 116.4, 114.9, 112.2, 53.1, 20.2; HRMS (ESI/Q-TOF) *m*/*z* [M + H]^+^ Calcd for C_13_H_12_N_3_O_2_ 242.0924; Found 242.0932.

### Methyl 3,6,9-Trimethyl-9*H*-pyridazino[3,4-*b*]indole-4-carboxylate

Compound **2q** was isolated by column chromatography (ethyl acetate/cyclohexane 50:50) in 79% yield (42.7 mg); yellow solid; mp 118–120 °C. ^1^H NMR (400 MHz, DMSO-*d*_6_) δ 7.84 (d, *J* = 1.2 Hz, 1H), 7.67 (d, *J* = 8.4 Hz, 1H), 7.60 (dd, *J* = 8.4, 1.2 Hz, 1H), 4.13 (s, 3H), 4.01 (s, 3H), 2.80 (s, 3H), 2.47 (s, 3H); ^13^C{^1^H} NMR (100 MHz, DMSO-*d*_6_) δ 166.7, 152.9, 146.6, 141.0, 132.6, 129.9, 124.4, 121.7, 115.9, 114.7, 110.4, 53.3, 28.2, 21.0, 20.2; HRMS (ESI/Q-TOF) *m*/*z* [M + H]^+^ Calcd for C_15_H_16_N_3_O_2_ 270.1237; Found 270.1255.

### Methyl 6-Methoxy-3,9-dimethyl-9*H*-pyridazino[3,4-*b*]indole-4-carboxylate

Compound **2r** was isolated by column chromatography (ethyl acetate/cyclohexane 50:50) in 81% yield (46.1 mg); yellow solid; mp 117–119 °C. ^1^H NMR (400 MHz, DMSO-*d*_6_) δ 7.71 (d, *J* = 9.2 Hz, 1H), 7.53 (d, *J* = 2.4 Hz, 1H), 7.43 (dd, *J* = 9.2, 2.4 Hz, 1H), 4.13 (s, 3H), 4.00 (s, 3H), 3.85 (s, 3H), 2.82 (s, 3H); ^13^C{^1^H} NMR (100 MHz, DMSO-*d*_6_) δ 166.5, 153.9, 153.0, 146.5, 137.7, 121.4, 120.8, 116.1, 114.6, 111.5, 106.7, 55.5, 53.1, 28.2, 20.3; HRMS (ESI/Q-TOF) *m*/*z* [M + H]^+^ Calcd for C_15_H_16_N_3_O_3_ 286.1186; Found 286.1183.

### Methyl 5-(Benzyloxy)-9-methyl-9*H*-pyridazino[3,4-*b*]indole-4-carboxylate

Compound **2s** was isolated by column chromatography (ethyl acetate/cyclohexane 50:50) in 80% yield (57.9 mg); yellow solid; mp 176–178 °C. ^1^H NMR (400 MHz, DMSO-*d*_6_) δ 7.60 (t, *J* = 8.4 Hz, 1H), 7.48–7.44 (m, 2H), 7.37–7.33 (m, 2H), 7.30–7.26 (m, 2H), 6.85 (d, *J* = 8.0 Hz, 1H), 5.46 (s, 2H), 3.99 (s, 3H), 3.79 (s, 3H), 2.69 (s, 3H); ^13^C{^1^H} NMR (100 MHz, DMSO-*d*_6_) δ 166.8, 155.9, 152.0, 145.8, 143.9, 136.5, 132.4, 128.5, 127.8, 127.4, 124.0, 112.6, 106.0, 103.7, 102.7, 69.5, 52.4, 28.3, 19.4; HRMS (ESI/Q-TOF) *m*/*z* [M + H]^+^ Calcd for C_21_H_20_N_3_O_3_ 362.1499; Found 362.1505.

### Methyl 8-Chloro-3,9-dimethyl-9*H*-pyridazino[3,4-*b*]indole-4-carboxylate

Compound **2t** was isolated by column chromatography (ethyl acetate/cyclohexane 50:50) in 79% yield (45.8 mg); orange solid; mp 140–142 °C. ^1^H NMR (400 MHz, DMSO-*d*_6_) δ 8.00 (d, *J* = 8.0 Hz, 1H), 7.75 (d, *J* = 8.0 Hz, 1H), 7.28 (t, *J* = 8.0 Hz, 1H), 4.35 (s, 3H), 4.12 (s, 3H), 2.80 (s, 3H); ^13^C{^1^H} NMR (100 MHz, DMSO-*d*_6_) δ 166.2, 153.2, 147.5, 137.8, 132.5, 123.9, 122.0, 121.8, 119.1, 116.3, 114.2, 53.4, 31.2, 20.1; HRMS (ESI/Q-TOF) *m*/*z* [M + H]^+^ Calcd for C_14_H_13_ClN_3_O_2_ 290.0691; Found 290.0697.

### Methyl 5-Chloro-3,9-dimethyl-9*H*-pyridazino[3,4-*b*]indole-4-carboxylate

Compound **2u** was isolated by column chromatography (ethyl acetate/cyclohexane 50:50) in 65% yield (37.4 mg); orange solid; mp 144–146 °C. ^1^H NMR (400 MHz, DMSO-*d*_6_) δ 7.77–7.72 (m, 2H), 7.39 (dd, *J* = 7.2, 1.6 Hz, 1H), 4.04 (s, 3H), 3.99 (s, 3H), 2.72 (s, 3H); ^13^C{^1^H} NMR (100 MHz, DMSO-*d*_6_) δ 167.1, 151.7, 146.2, 143.9, 131.7, 129.8, 124.3, 121.9, 114.1, 111.7, 109.6, 52.9, 28.6, 19.6; HRMS (ESI/Q-TOF) *m*/*z* [M + H]^+^ Calcd for C_14_H_13_ClN_3_O_2_ 290.0691; Found 290.0705.

### Methyl 6-Bromo-3,9-dimethyl-9*H*-pyridazino[3,4-*b*]indole-4-carboxylate

Compound **2v** was isolated by column chromatography (ethyl acetate/cyclohexane 50:50) in 80% yield (53.7 mg); yellow solid; mp 131–133 °C. ^1^H NMR (400 MHz, DMSO-*d*_6_) δ 8.28 (d, *J* = 2.0 Hz, 1H), 7.93 (dd, *J* = 8.8, 2.0 Hz, 1H), 7.80 (d, *J* = 8.8 Hz, 1H), 4.13 (s, 3H), 4.04 (s, 3H), 2.86 (s, 3H); ^13^C{^1^H} NMR (100 MHz, DMSO-*d*_6_) δ 166.3, 152.9, 147.7, 141.6, 133.7, 127.5, 121.8, 117.7, 114.2, 112.8, 112.5, 53.3, 28.3, 20.6; HRMS (ESI/Q-TOF) *m*/*z* [M + H]^+^ Calcd for C_14_H_13_BrN_3_O_2_ 334.0186; Found 334.0182.

### Methyl 7-Fluoro-3,9-dimethyl-9*H*-pyridazino[3,4-*b*]indole-4-carboxylate

Compound **2w** was isolated by column chromatograpy (ethyl acetate/cyclohexane 30:70) in 80% yield (43.5 mg); yellow solid; mp 188–190 °C. ^1^H NMR (400 MHz, DMSO-*d*_6_) δ 8.12 (dd, *J* = 8.8 Hz, ^4^*J*_HF_ = 5.2 Hz, 1H), 7.66 (dd, ^3^*J*_HF_*=* 10.4 Hz, *J* = 2.4 Hz, 1H), 7.17–7.12 (m, 1H), 4.11 (s, 3H), 3.98 (s, 3H), 2.80 (s, 3H); ^13^C{^1^H} NMR (100 MHz, CDCl_3_) δ 167.2, 164.9 (d, ^1^*J*_CF_ = 249.4 Hz), 154.1, 148.7, 144.7 (d, ^3^*J*_CF_ = 12.5 Hz), 128.0 (d, ^3^*J*_CF_ = 10.9 Hz), 121.8, 116.5, 113.6, 109.6 (d, ^2^*J*_CF_ = 24.1 Hz), 96.6 (d, ^2^*J*_CF_ = 26.8 Hz), 53.0, 28.5, 21.1; HRMS (ESI/Q-TOF) *m*/*z* [M + H]^+^ Calcd for C_14_H_13_FN_3_O_2_ 274.0986; Found 274.0993.

### Dimethyl 3,9-Dimethyl-9*H*-pyridazino[3,4-*b*]indole-4,5-dicarboxylate

Compound **2x** was isolated by column chromatography (ethyl acetate/cyclohexane 50:50) in 73% yield (45.8 mg); brown solid; mp 136–138 °C. ^1^H NMR (400 MHz, DMSO-*d*_6_) δ 8.07 (dd, *J* = 8.4, 0.8 Hz, 1H), 7.87 (dd, *J* = 8.4, 7.6 Hz, 1H), 7.79 (dd, *J* = 7.6, 0.8 Hz, 1H), 4.12 (s, 3H), 3.93 (s, 3H), 3.91 (s, 3H), 2.85 (s, 3H); ^13^C{^1^H} NMR (100 MHz, DMSO-*d*_6_) δ 167.6, 166.4, 152.5, 147.3, 143.1, 130.4, 129.6, 123.4, 122.4, 114.6, 113.7, 113.5, 52.5, 52.5, 28.4, 20.7; HRMS (ESI/Q-TOF) *m*/*z* [M + H]^+^ Calcd for C_16_H_16_N_3_O_4_ 314.1135; Found 314.1146.

### Methyl 10-Methyl-5,6-dihydro-4*H*-pyridazino[4′,3′:4,5]pyrrolo[3,2,1-*ij*]quinoline-11-carboxylate

Compound **2y** was isolated by column chromatography (ethyl acetate/cyclohexane 50:50) in 67% yield (37.5 mg); yellow solid; mp 130–132 °C. ^1^H NMR (400 MHz, DMSO-*d*_6_) δ 7.92 (d, *J* = 8.0 Hz, 1H), 7.50 (dd, *J* = 7.2, 0.8 Hz, 1H), 7.23 (dd, *J* = 8.0, 7.2 Hz, 1H), 4.46 (t, *J* = 6.0 Hz, 2H), 4.12 (s, 3H), 3.08 (t, *J* = 6.0 Hz, 2H), 2.83 (s, 3H), 2.24 (quint, *J* = 6.0 Hz, 2H); ^13^C{^1^H} NMR (100 MHz, DMSO-*d*_6_) δ 166.6, 152.0, 146.8, 139.5, 128.6, 122.6, 122.6, 121.8, 120.7, 115.5, 114.1, 53.1, 40.2, 24.2, 21.2, 20.4; HRMS (ESI/Q-TOF) *m*/*z* [M + H]^+^ Calcd for C_16_H_16_N_3_O_2_ 282.1237; Found 282.1239.

### Methyl 1-((Methoxycarbonyl)amino)-2,8-dimethyl-1,8-dihydropyrrolo[2,3-*b*]indole-3-carboxylate

Intermediate **C** (entries 22, 25, and 27, Table 1) was isolated as a byproduct by column chromatography (ethyl acetate/cyclohexane 40:60); mp 164–166 °C. ^1^H NMR (400 MHz, DMSO-*d*_6_) δ 10.99 (br, 1H), 7.93 (dd, *J* = 8.0, 0.8 Hz, 1H), 7.44 (d, *J* = 8.0 Hz, 1H), 7.19–7.14 (m, 1H), 7.12–7.08 (m, 1H), 3.89 (s, 3H), 3.79 (s, 6H), 2.48 (s, 3H); ^13^C{^1^H} NMR (100 MHz, DMSO-*d*_6_) δ 165.1, 156.2, 139.7, 136.5, 136.3, 120.6, 120.1, 119.7, 119.2, 109.5, 102.7, 102.1, 53.3, 50.9, 29.1, 10.2; HRMS (ESI/Q-TOF) *m*/*z* [M + H]^+^ Calcd for C_16_H_18_N_3_O_4_ 316.1292; Found 316.1288.

### Ethyl 1-Amino-2,8-dimethyl-1,8-dihydropyrrolo[2,3-*b*]indole-3-carboxylate

Compound **D1** was isolated by column chromatography (ethyl acetate/cyclohexane 30:70) in 30% yield (22.5 mg); red solid; mp 168–170 °C. ^1^H NMR (400 MHz, DMSO-*d*_6_) δ 7.93–7.91 (m, 1H), 7.39 (d, *J* = 8.0 Hz, 1H), 7.12–7.08 (m, 1H), 7.06–7.02 (m, 1H), 6.02 (s, 2H), 4.31 (q, *J* = 7.2 Hz, 2H), 4.00 (s, 3H), 2.62 (s, 3H), 1.40 (t, *J* = 7.2 Hz, 3H); ^13^C{^1^H} NMR (100 MHz, DMSO-*d*_6_) δ 165.0, 139.9, 137.5, 137.4, 120.4, 119.8, 119.5, 118.6, 109.1, 102.1, 100.2, 58.9, 29.7, 14.7, 10.7; HRMS (ESI/Q-TOF) *m*/*z* [M + H]^+^ Calcd for C_15_H_18_N_3_O_2_ 272.1394; Found 272.1388.

### Hydrolysis of **2a**

To a solution of **2a** (127.6 mg, 0.5 mmol) in MeOH (5 mL) was added KOH (280.0 mg, 5 mmol, 10 equiv). The mixture was refluxed (heating mantle) until the disappearance of **2a** (1.5 h, TLC check). The reaction mixture was cooled to r.t. and the solvent evaporated *in vacuo*. The residue was dissolved in water (2 mL) and acidified to pH 2 via the addition of 4 N aq HCl under stirring at 0 °C. The precipitate was filtered off, washed with diethyl ether, and dried to afford compound **3** as a yellow solid.

### 3,9-Dimethyl-9*H*-pyridazino[3,4-*b*]indole-4-carboxylic Acid

compound **3** was isolated in 95% yield (114.2 mg); yellow solid; mp 248–250 °C. ^1^H NMR (400 MHz, DMSO-*d*_6_) δ 14.57 (br, 1H), 8.22 (dt, *J* = 8.0, 0.8 Hz, 1H), 7.80–7.77 (m, 2H), 7.39–7.35 (m, 1H), 4.04 (s, 3H), 2.84 (s, 3H); ^13^C{^1^H} NMR (100 MHz, DMSO-*d*_6_) δ 167.2, 152.8, 146.4, 143.2, 131.6, 125.1, 124.5, 121.1, 116.1, 116.0, 110.7, 28.3, 19.5; HRMS (ESI/Q-TOF) *m*/*z* [M + H]^+^ Calcd for C_13_H_12_N_3_O_2_ 242.0924; Found 242.0916.

### Decarboxylation of **3**

To a solution of compound **3** (48.2 mg, 0.2 mmol) in DMSO/water (10:1, 2 mL) was added NaCl (81.8 mg, 1.4 mmol, 7 equiv). The solution was stirred at 140 °C (oil bath) until the disappearance of the starting material (24 h, TLC check). After cooling to room temperature, the mixture was diluted with water (5 mL) and extracted with ethyl acetate (3 × 10 mL), washed with brine (10 mL), and dried over anhydrous sodium sulfate. The residue was purified by column chromatography on silica gel to give the product **4**.

### 3,9-Dimethyl-9*H*-pyridazino[3,4-*b*]indole

Compound **4** was isolated by column chromatography (ethyl acetate/cyclohexane 20:80) in 92% yield (36.2 mg); light brown solid; mp 142–144 °C. ^1^H NMR (400 MHz, DMSO-*d*_6_) δ 8.31–8.30 (m, 1H), 8.28 (s, 1H), 7.74–7.69 (m, 2H), 7.36–7.32 (m, 1H), 3.98 (s, 3H), 2.78 (s, 3H); ^13^C{^1^H} NMR (100 MHz, DMSO-*d*_6_) δ 152.4, 150.7, 142.2, 130.4, 123.7, 120.4, 119.5, 118.0, 117.6, 110.2, 28.0, 21.5; HRMS (ESI/Q-TOF) *m*/*z* [M + H]^+^ Calcd for C_12_H_12_N_3_ 198.1026; Found 198. 1031.

## Supporting Information Available

Copies of NMR spectra for all products (PDF)
FAIR data, including the primary NMR FID files, for compounds **1a**–**z**, **2a**–**y**, **C**, **D1**, **3**, and **4** (ZIP)

## Dedication

This work is dedicated to Prof. Paolino Filippone, colleague, coauthor, and friend, who passed away on September 17, 2020.
