# Supplementary material for: Synthesis of Azacarbolines via PhIO2-Promoted Intramolecular Oxidative Cyclization of α-Indolylhydrazones
Source: J Org Chem. 2021 Dec 6;86(24):17918–29. doi: 10.1021/acs.joc.1c02217 (PMC8689645; doi:10.1021/acs.joc.1c02217)

Synthesis of Azacarbolines via $PhIO_2$-Promoted Intramolecular Oxidative Cyclization of α-Indolylhydrazones

Matteo Corrieri, Lucia De Crescentini, Fabio Mantellini, Giacomo Mari, Stefania Santeusanio, and Gianfranco Favi*

[a]*Department of Biomolecular Sciences, Section of Chemistry and Pharmaceutical Technologies, University of Urbino "Carlo Bo", Via I Maggetti 24, 61029 Urbino, Italy*

e-mail: gianfranco.favi@uniurb.it

SUPPORTING INFORMATION

Table of Contents

1.	**General remarks**	S2
2.	**Experimental procedures**	S3–S4
	General procedure for the preparation of α-(indol-3-yl)hydrazones **1a–i,k–z**	S3
	Procedure for the preparation of bis(indolyl)methane hydrazone **1j**	S3
	General procedure for the synthesis of azacarbolines **2a–y**	S5
	Hydrolysis of **2a**	S5
	Decarboxylation of **3**	S5
3.	**References and notes**	S5
4.	**^{1}H and ^{13}C NMR spectra of all products**	S6–S59

FAIR Data is available as Supporting Information for Publication and includes the primary NMR FID files for compounds: [**1a–z**, **2a–y**, **C**, **D1**, **3** and **4**].

1. General Remarks

All the commercially available reagents and solvents were used without further purification. α-(Indol-3-yl)hydrazones **1a–i,k–z** were prepared according to our previously reported method with a slight modification.[1a],[1b] Bis(indolyl)methane hydrazone **1j** was prepared following literature procedure.[1c] Chromatographic purification of compounds was carried out on silica gel (60–200 μm). TLC analysis was performed on pre-loaded (0.25 mm) glass supported silica gel plates (Kieselgel 60); compounds were visualized by exposure to UV light and by dipping the plates in 1% $Ce(SO_4)\cdot 4H_2O$, 2.5% $(NH_4)_6Mo_7O_{24}\cdot 4H_2O$ in 10% sulphuric acid followed by heating on a hot plate. All ^{1}H NMR and ^{13}C NMR spectra were recorded at 400 and 100 MHz using DMSO-*d*$_6$ or $CDCl_3$ as solvent on a Bruker Ultrashield 400 spectrometer (Bruker, Billerica, MA, USA). Chemical shift (δ scale) are reported in parts per million (ppm) relative to the central peak of the solvent and are sorted in descending order within each group. The following abbreviations are used to describe peak patterns where appropriate: s = singlet, d = doublet, dd = doublet of doublet, dt = doublet of triplet, t = triplet, q = quartet, sex = sextet, sept = septet, m = multiplet and br = broad signal. All coupling constants (J value) are given in Hertz [Hz]. High-resolution mass spectroscopy was performed on a Micromass Q-TOF Micro mass spectrometer (Micromass, Manchester, UK) using an ESI source. Melting points were determined in open capillary tubes and are uncorrected.

2. Experimental procedures and spectral data

General procedure for the preparation of α-(indol-3-yl)hydrazones 1a–i,k–z[1a],[1b]:

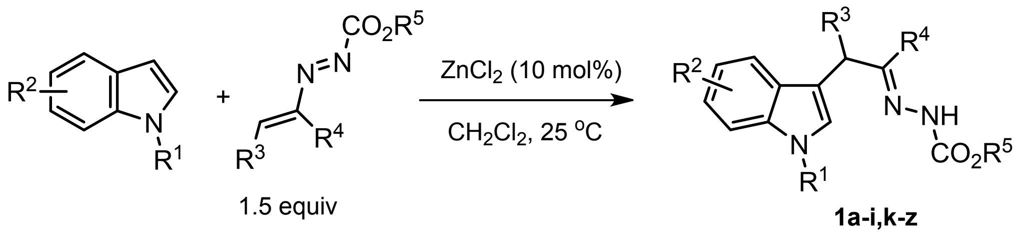

1a-i,k-z

To a stirred mixture of indole (1 mmol) and azoalkene (1.5 mmol, 1.5 equiv) in dichloromethane (4 mL), zinc dichloride (13.6 mg, 0.1 mmol, 10 mol %) was added. (In order to obtain the compound **1p**, the addition of DIPEA (174 μL, 1 mmol, 1 equiv) was required.) After the disappearance of indole (TLC check), the solvent was removed and the crude mixture was purified by column chromatography on silica gel to afford, after crystallization, the α-(indol-3-yl)hydrazones **1** (23–95% yields).

Procedure for the preparation of bis(indolyl)methane hydrazone 1j[1c]:

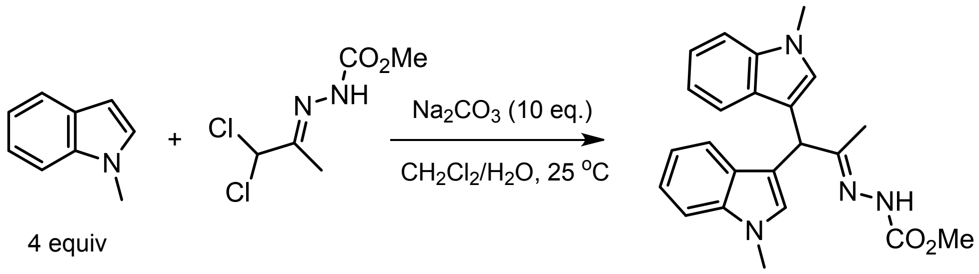

1j

1 Methylindole (0.75 mL, 6 mmol, 4 equiv) was added to a previously stirred solution of Na_2CO_3 (1.59 g, 15 mmol, 10 equiv) in water (5 mL). The dichloroacetone hydrazone (298.5 mg, 1.5 mmol) in dichloromethane (5 mL) were added and the reaction mixture was stirred at room temperature. Upon completion of the reaction (1 h, TLC check), the mixture was diluted with water (10 mL), extracted with di-chloromethane (3 x 20 mL), and the collected organic phases were dried over anhydrous Na_2SO_4. After filtration, the reaction was concentrated in vacuo, and the obtained crude was purified by flash chromatography to afford the bis(indolyl)methane hydrazone **1j** (23% yield).

List of substrates **1a–z** prepared according to the general procedures.[1]

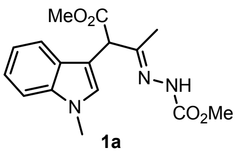
1a

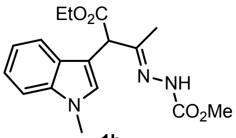
1b

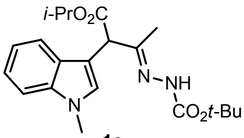
1c

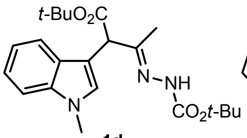
1d

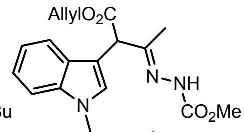
1e

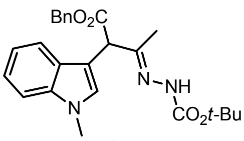
1f

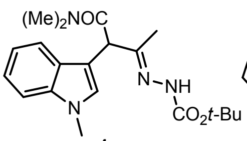
1g

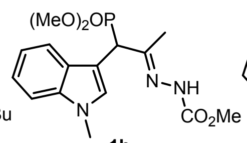
1h

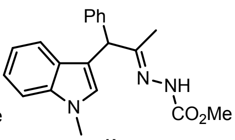
1i

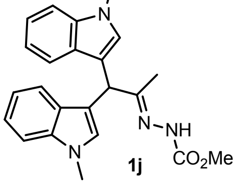
1j

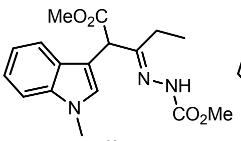
1k

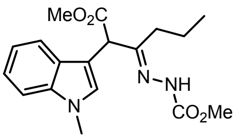
1l

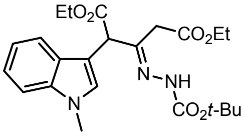
1m

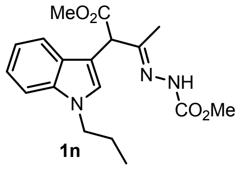
1n

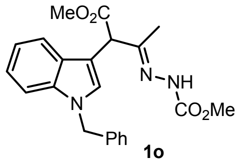
1o

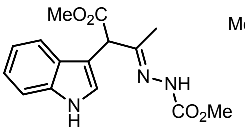
1p

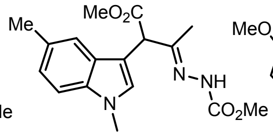
1q

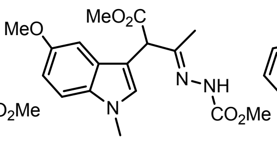
1r

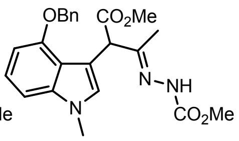
1s

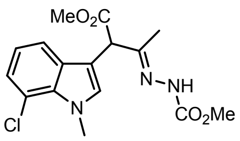
1t

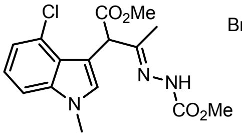
1u

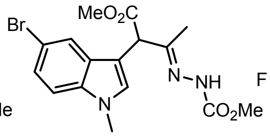
1v

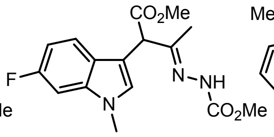
1w

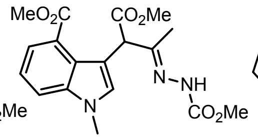
1x

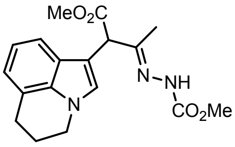
1y

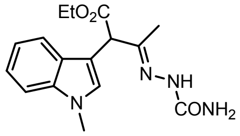
1z

General procedure for the synthesis of azacarbolines 2 via PhIO$_2$-mediated intramolecular oxidative cyclization of α-indolylhydrazones 1:

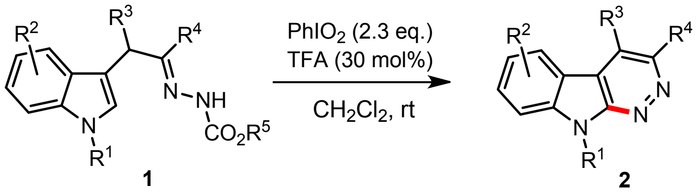

To a stirred mixture of α-indolylhydrazone **1** (0.2 mmol) in dichloromethane (2 mL), PhIO$_2$ (108.6 mg, 0.46 mmol, 2.3 equiv) and TFA (5 μL, 0.06 mmol, 30 mol%) were added. After that, the solution was stirred overnight at room temperature. The crude product was directly purified by flash chromatography on silica gel (cyclohexane/ethyl acetate) to give the corresponding product **2** (21–82% yields).

Hydrolysis of 2a: To a solution of **2a** (127.6 mg, 0.5 mmol) in MeOH (5 mL), KOH (280 mg, 5 mmol, 10 equiv) was added. The mixture was refluxed (heating mantle) until the disappearance of **2a** (1.5 h, TLC check). The reaction mixture was cooled to r.t. and the solvent evaporated in vacuo. The residue was dissolved in water (2 mL) and acidified to pH 2 via the addition of 4 N aq HCl under stirring at 0 °C. The precipitate was filtered off, then washed with diethyl ether and dried to afford the compound **3** (95% yield) as yellow solid.

Decarboxylation of 3: To a solution of compound **3** (48.2 mg, 0.2 mmol) in DMSO/water (10:1, 2 mL), NaCl (81.8 mg, 1.4 mmol, 7 equiv) was added. The solution was stirred at 140 °C (oil-bath) until the disappearance of the starting material (24 h, TLC check). After cooling to room temperature, the mixture was diluted with water (5 mL) and extracted with ethyl acetate (3 x 10 mL), washed with brine (10 mL) and dried over anhydrous sodium sulphate. The residue was purified by column chromatography on silica gel to give the product **4** (92% yield).

3. References

[1] (a) Ciccolini, C.; De Crescentini, L.; Mantellini, F.; Santeusanio, S.; Favi, G. *Org. Lett.* **2019**, *21*, 4388−4391. (b) Ciccolini, C.; Mari, G.; Gatti, G. F.; Gatti, G.; Giorgi, G.; Mantellini, F.; Favi, G. *J. Org. Chem.* **2020**, *85*, 11409–11425. (c) Grosso, C.; Cardoso, A. L.; Rodrigues, M. J.; Marques, C.; Barreira, L.; Lemos, A.; Pinho e Melo, T. M.D.V. *Bioorg. Med. Chem. Lett.* **2017**, *25*, 1122–1131.

3. ^{1}H and ^{13}C NMR spectra of all products

^{1}H NMR of 1a (400 MHz, DMSO-d_6)

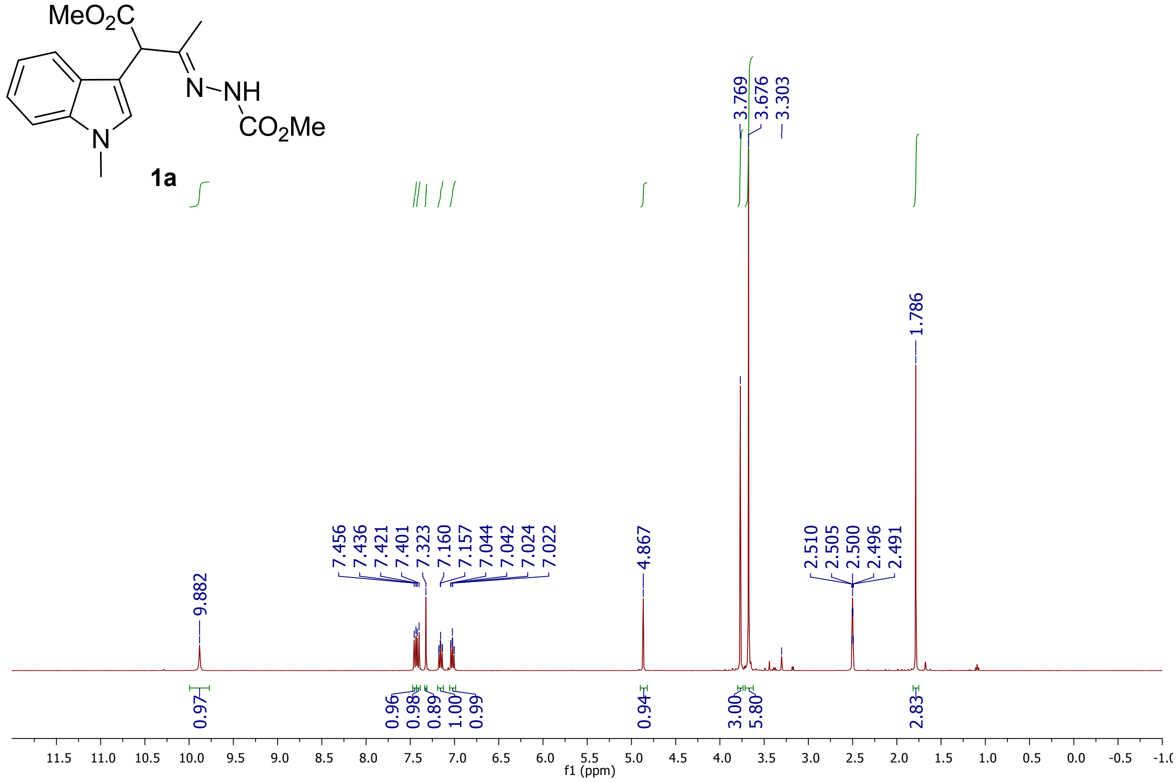

$^{13}C\{^{1}H\}$ NMR of 1a (100 MHz, DMSO-d_6)

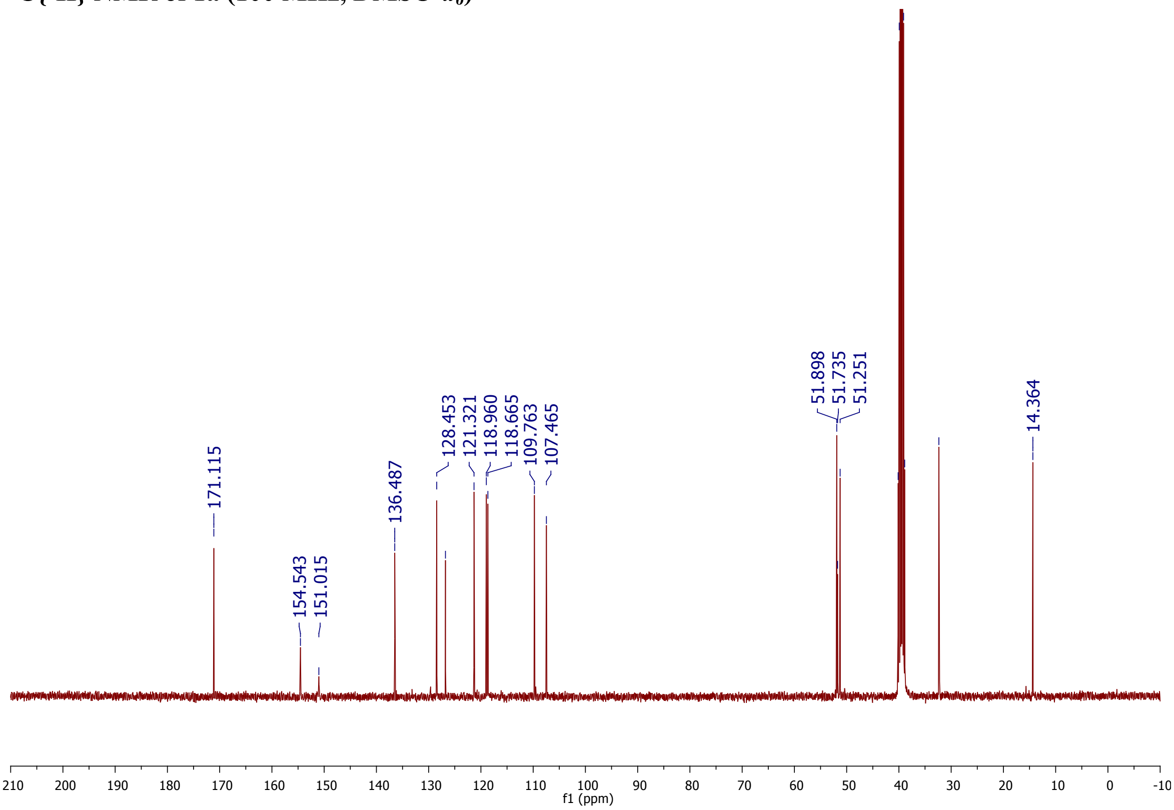

^{1}H NMR of 1b (400 MHz, DMSO-d_6)

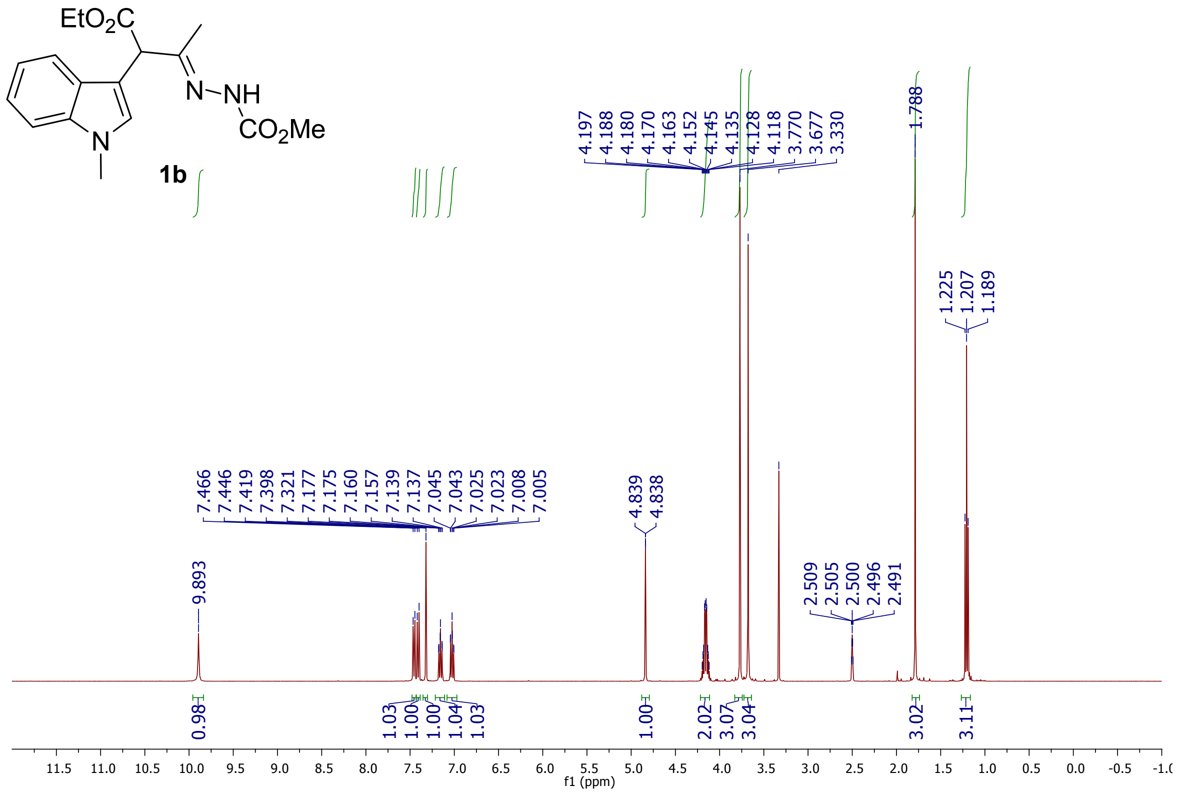

^{13}C{^{1}H} NMR of 1b (100 MHz, DMSO-d_6)

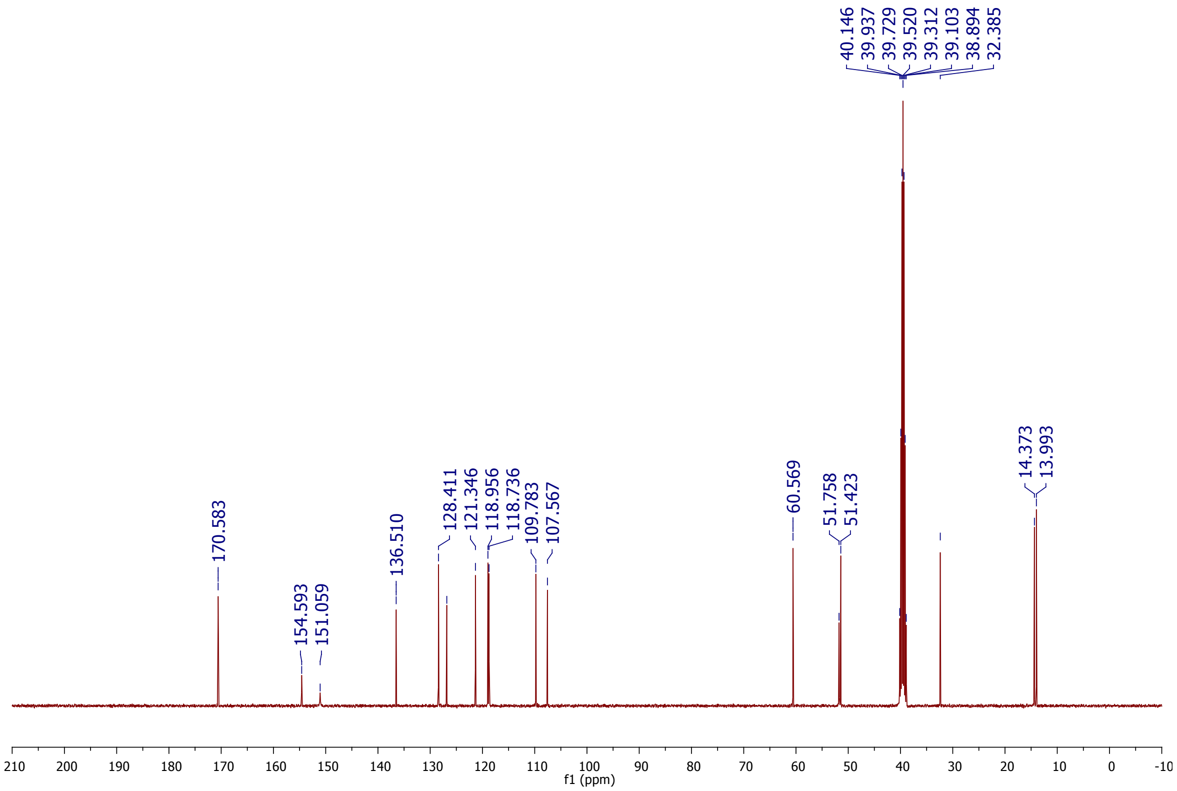

^{1}H NMR of 1c (400 MHz, DMSO-d_6)

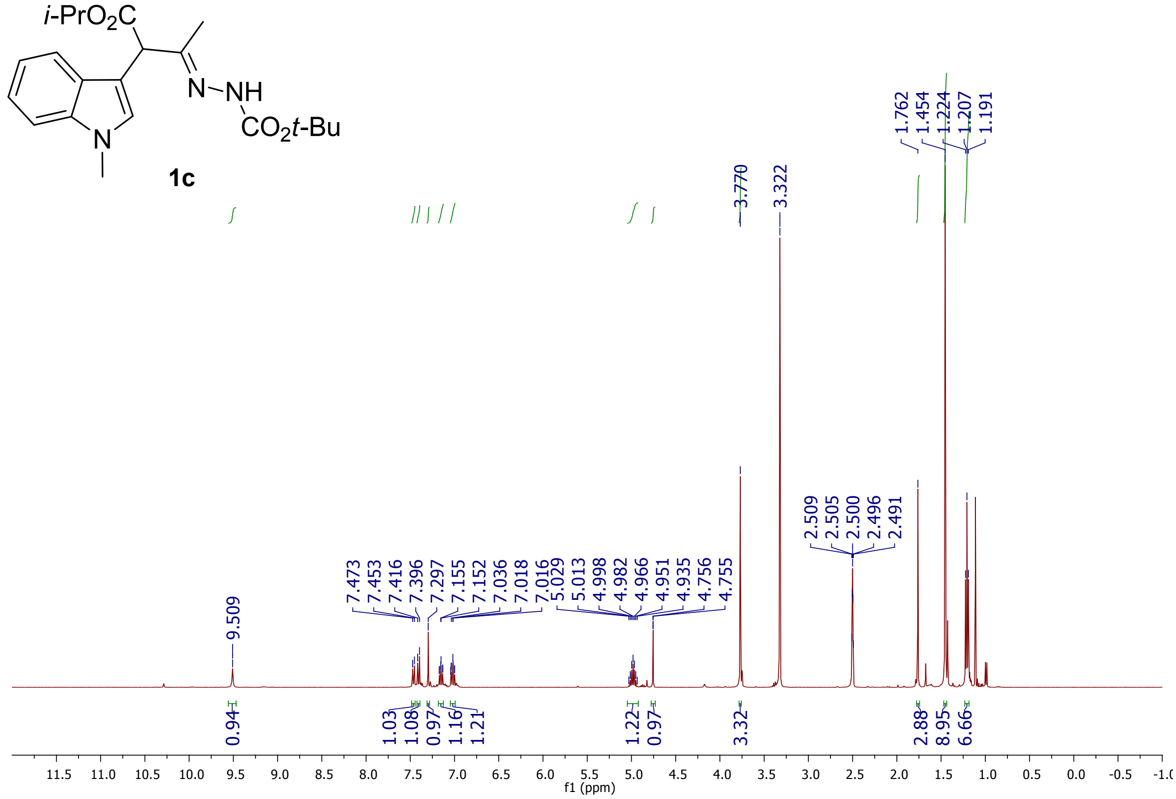

^{13}C{^{1}H} NMR of 1c (100 MHz, DMSO-d_6)

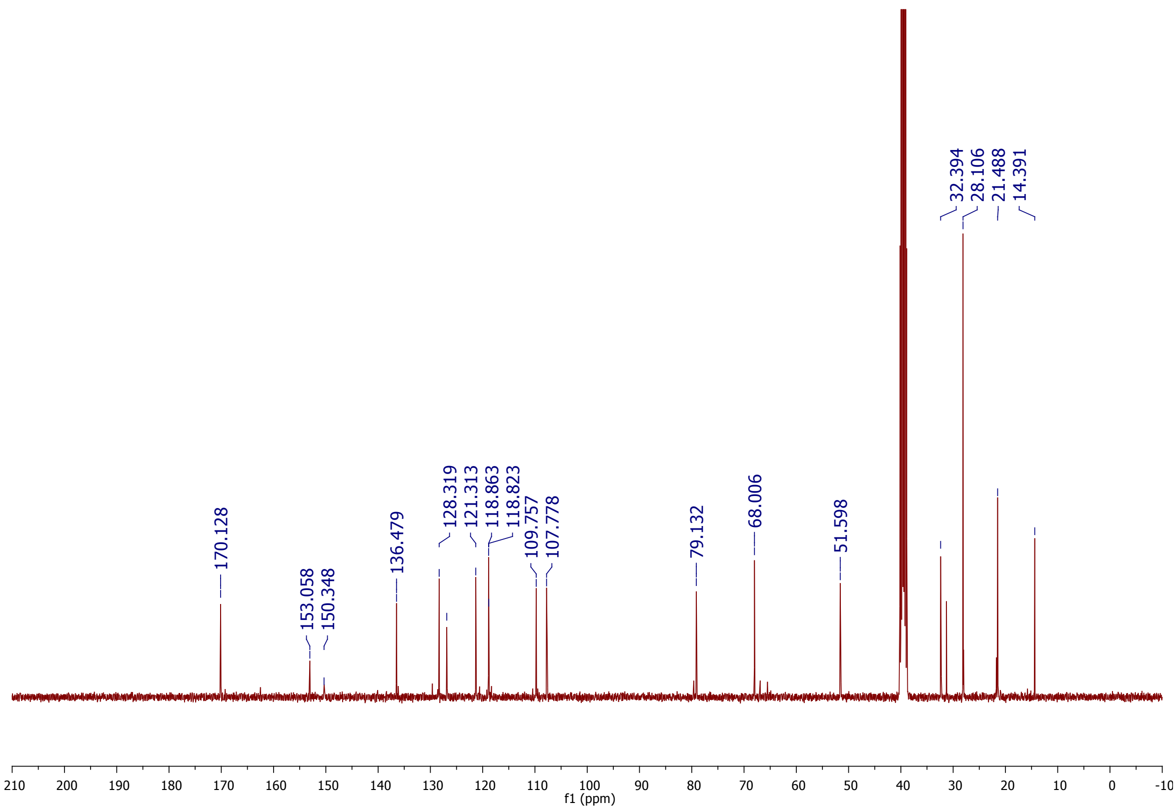

^{1}H NMR of 1d (400 MHz, DMSO-d_6)

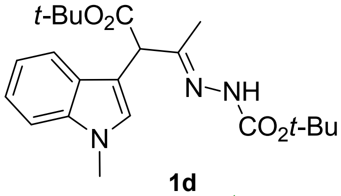

1d

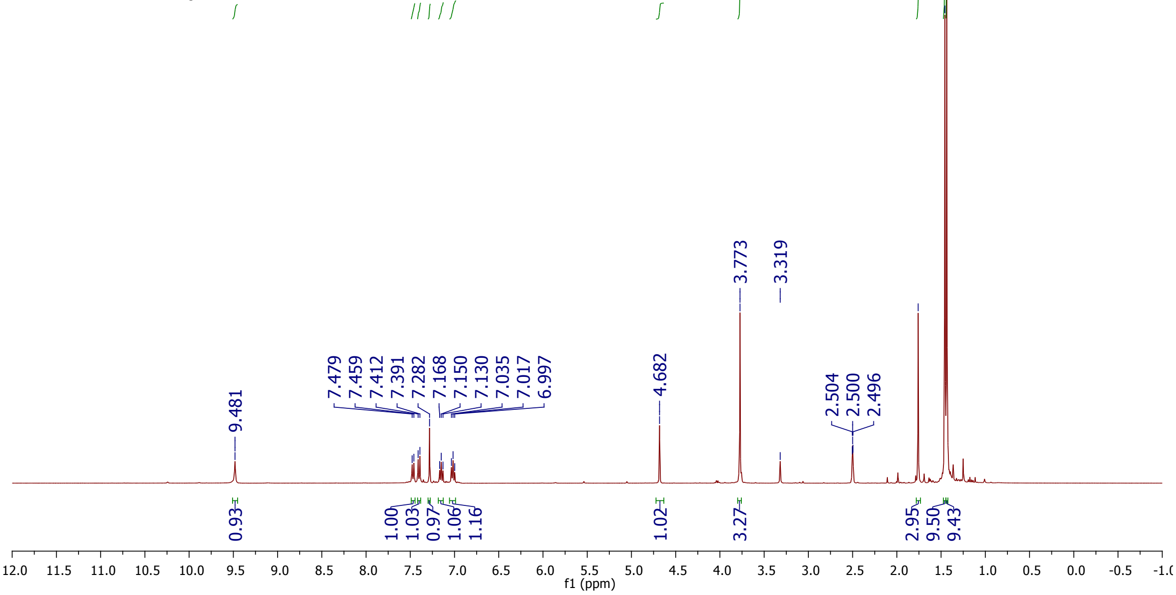

^{13}C{^{1}H} NMR of 1d (100 MHz, DMSO-d_6)

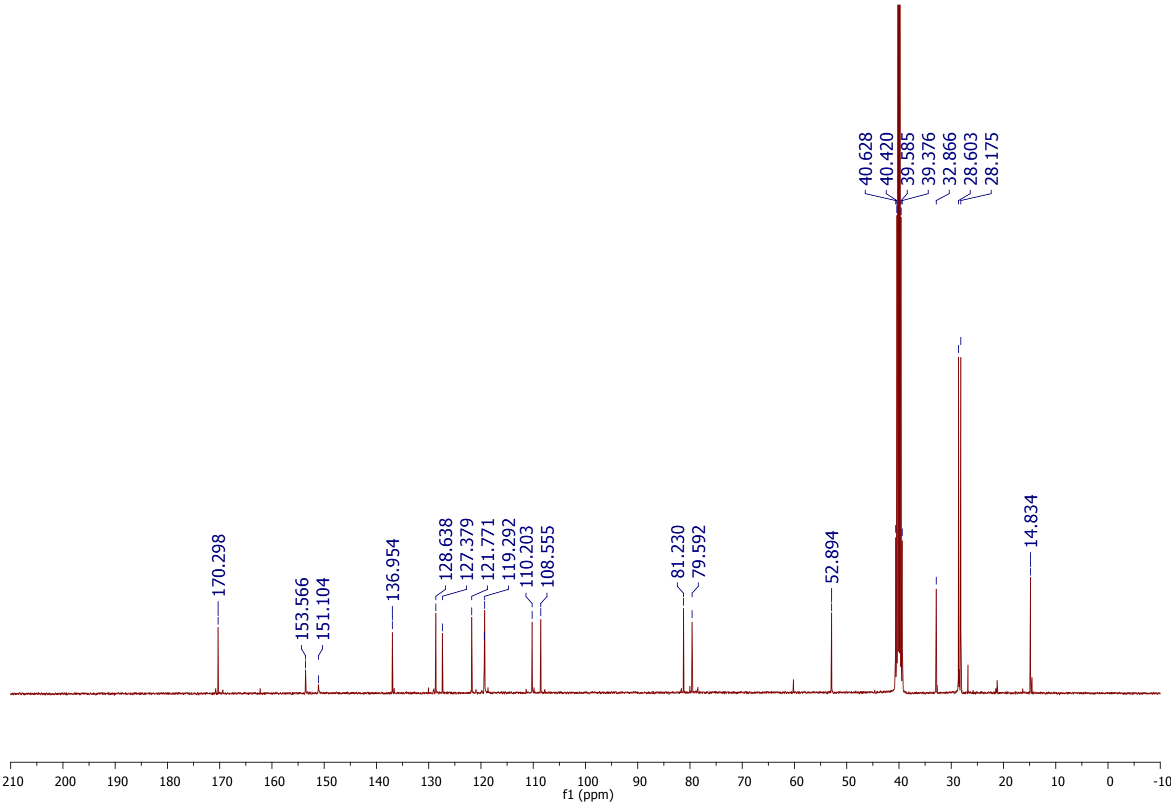

^{1}H NMR of 1e (400 MHz, DMSO-d_6)

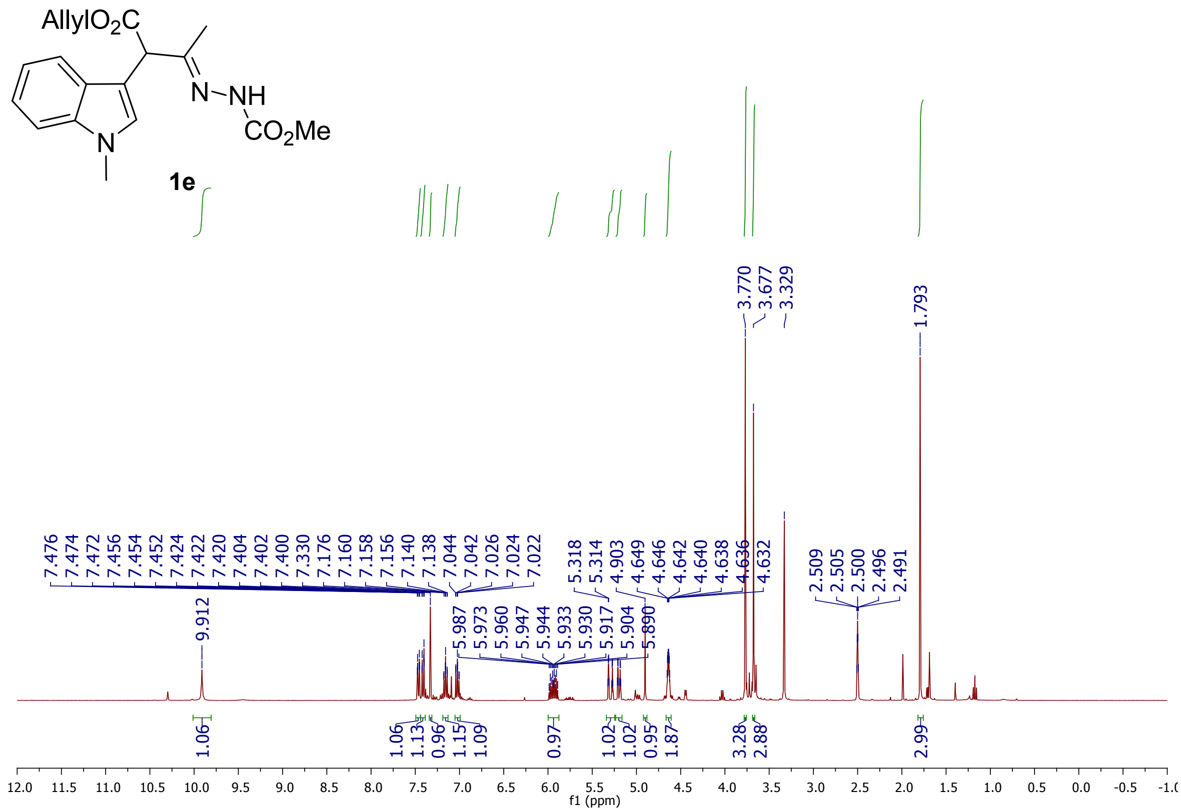

^{13}C{^{1}H} NMR of 1e (100 MHz, DMSO-d_6)

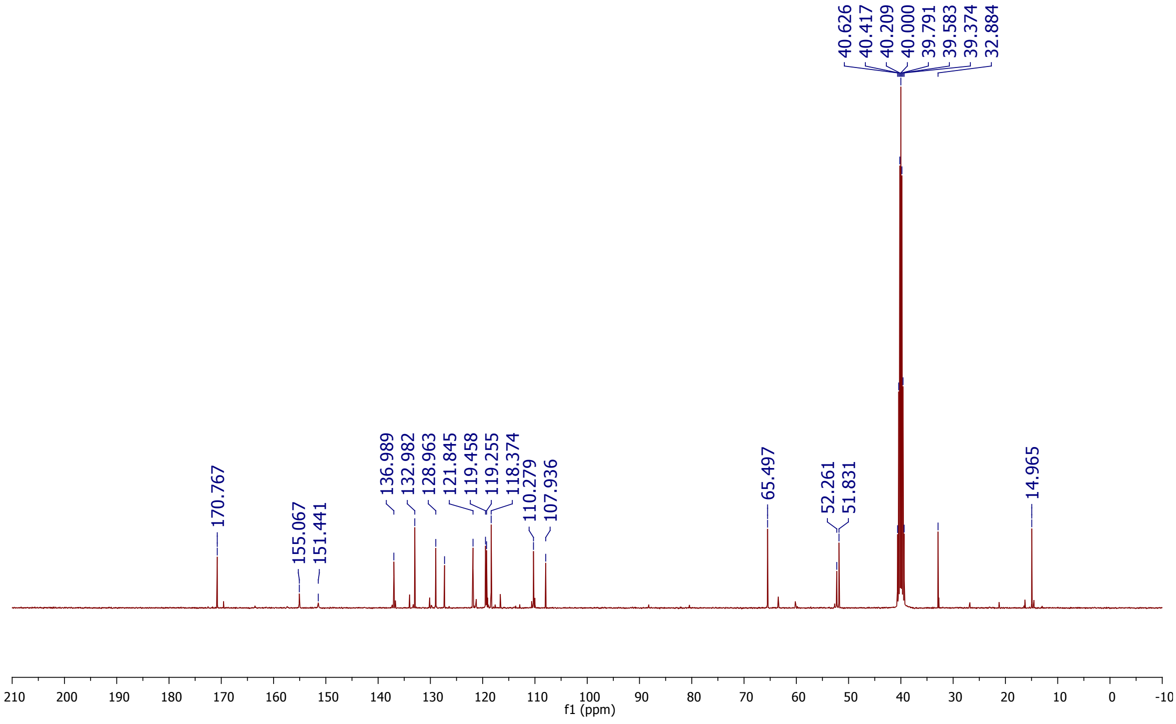

^{1}H NMR of 1f (400 MHz, DMSO-d_6)

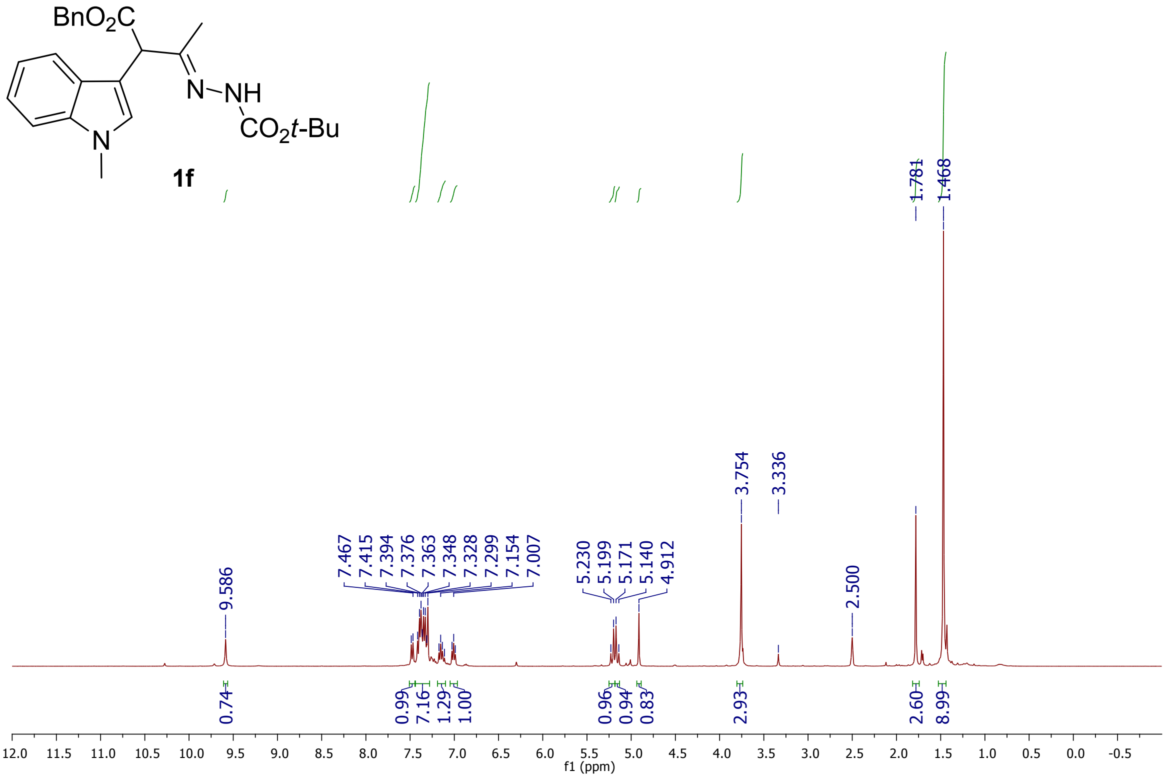

^{13}C{^{1}H} NMR of 1f (100 MHz, DMSO-d_6)

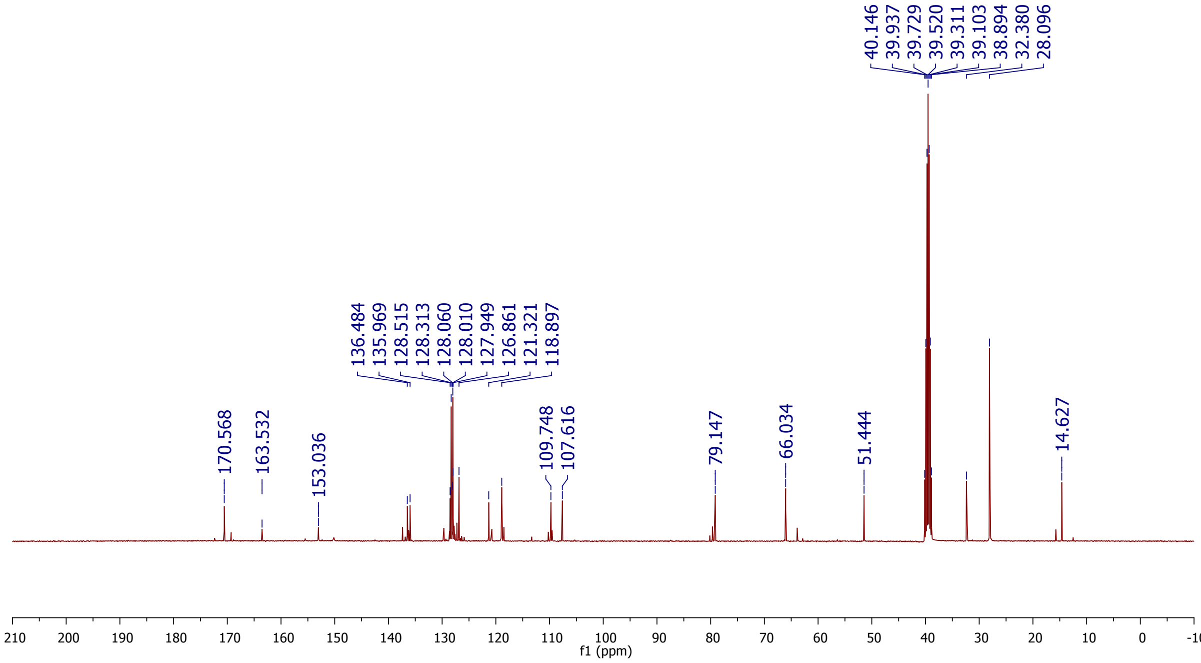

^{1}H NMR of 1g (400 MHz, DMSO-d_6)

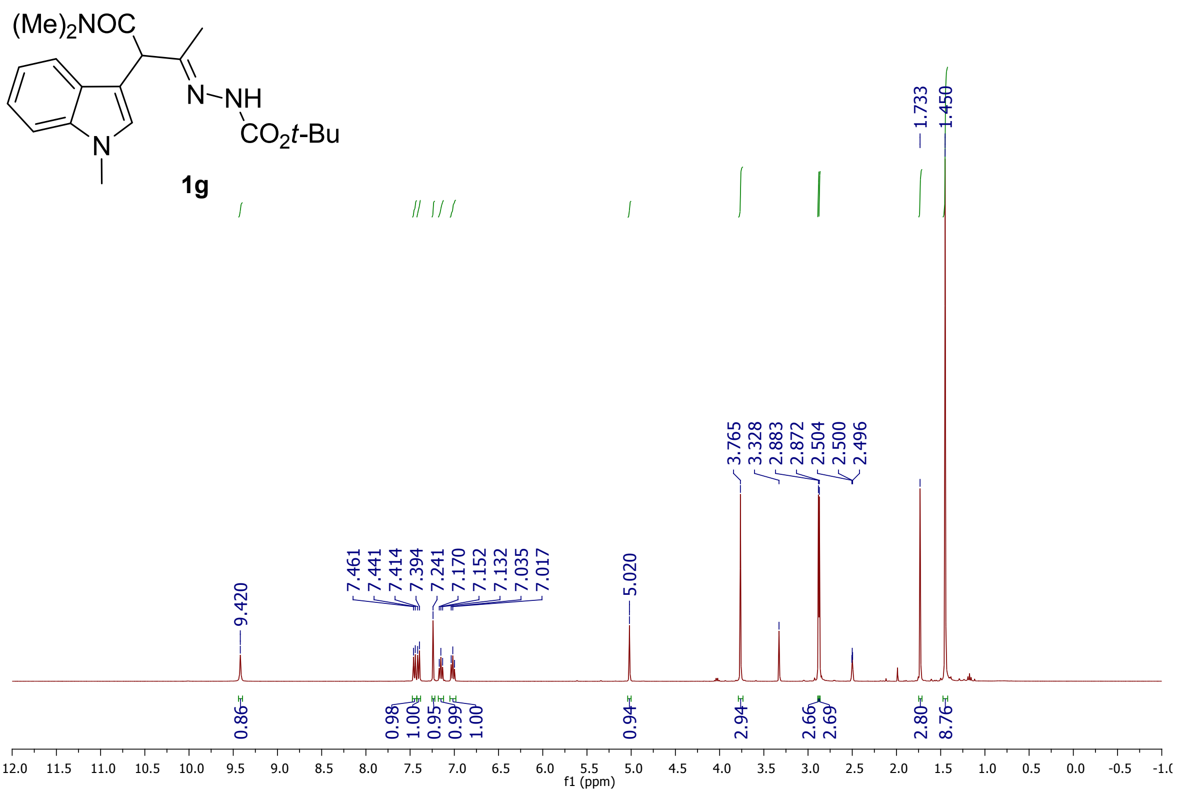

^{13}C{^{1}H} NMR of 1g (100 MHz, DMSO-d_6)

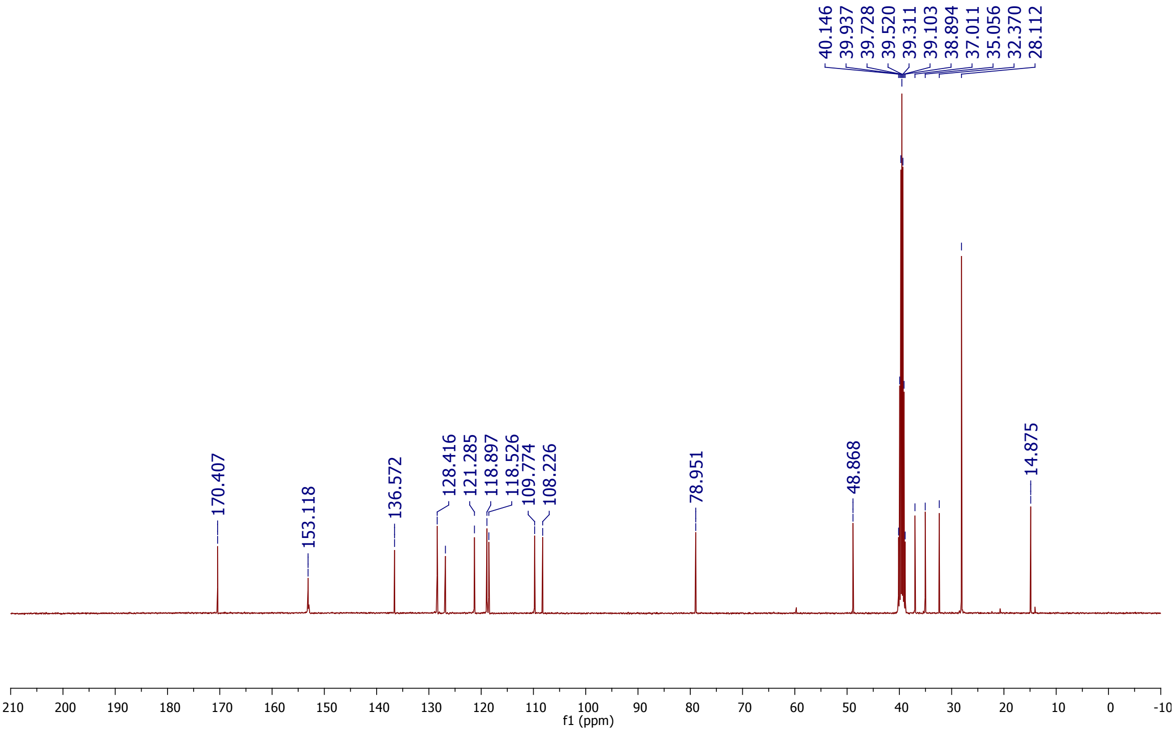

^{1}H NMR of 1h (400 MHz, DMSO-d_6)

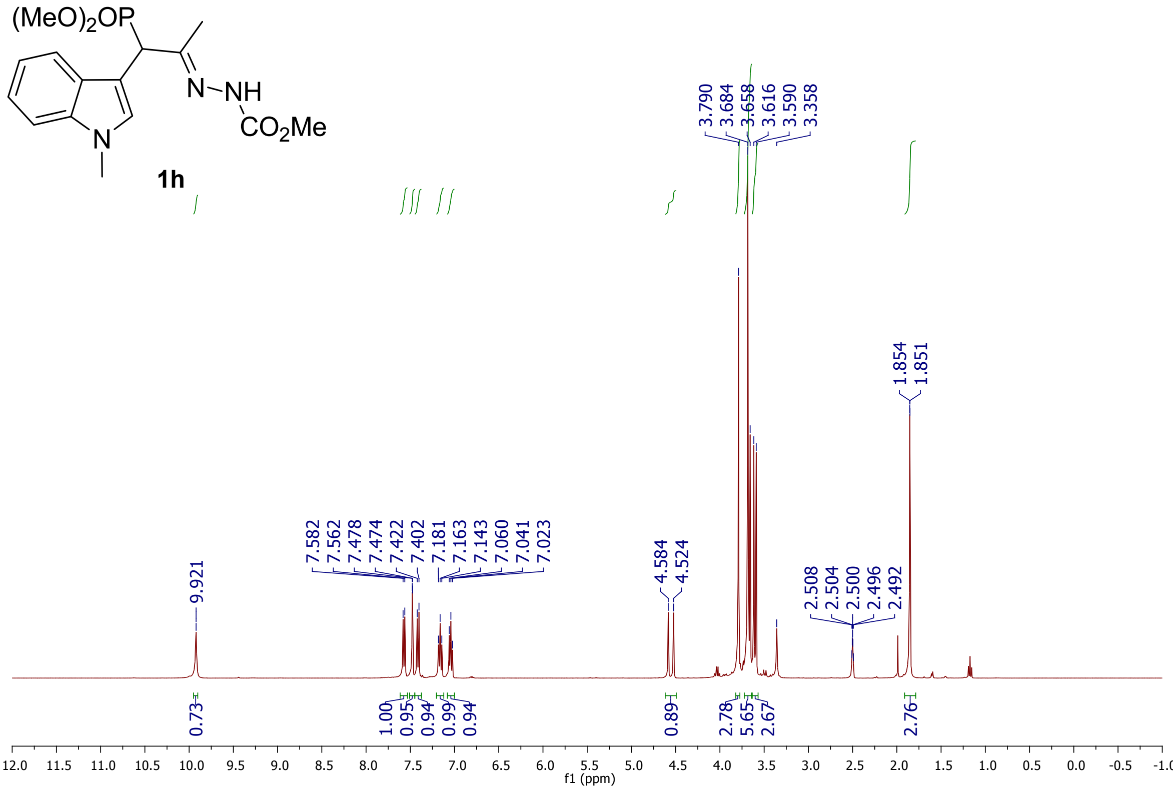

^{13}C{^{1}H} NMR of 1h (100 MHz, DMSO-d_6)

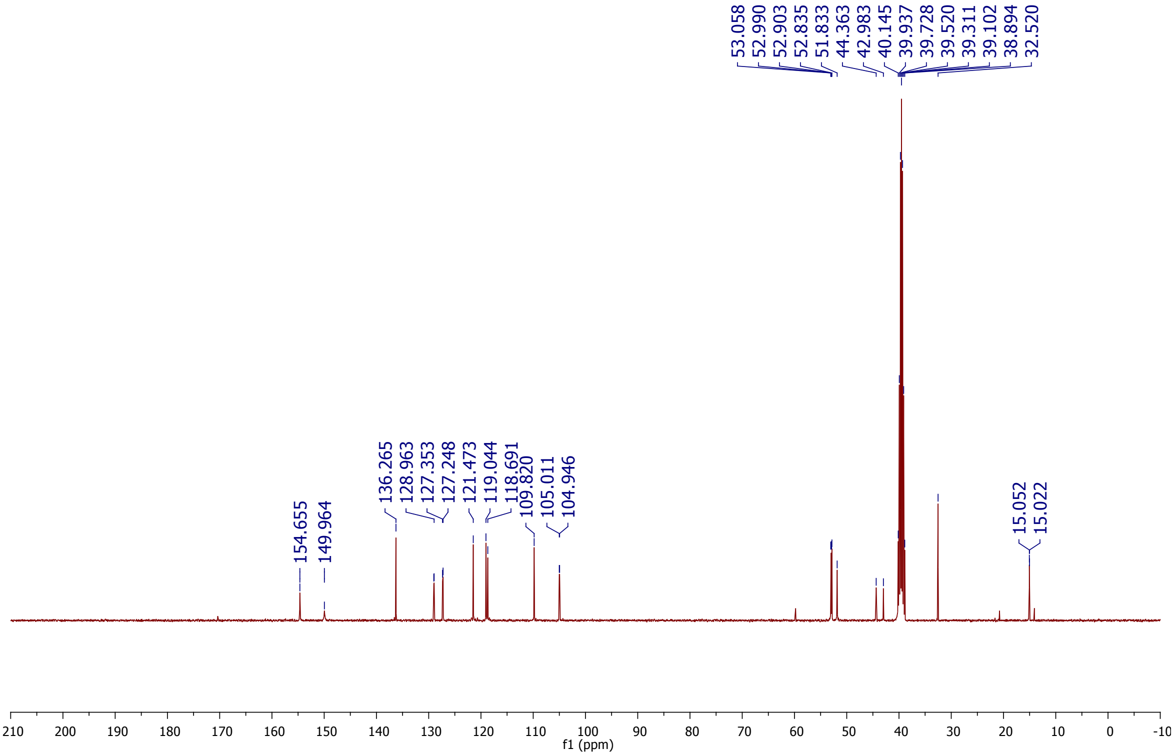

^{1}H NMR of 1i (400 MHz, DMSO-*d_6*)

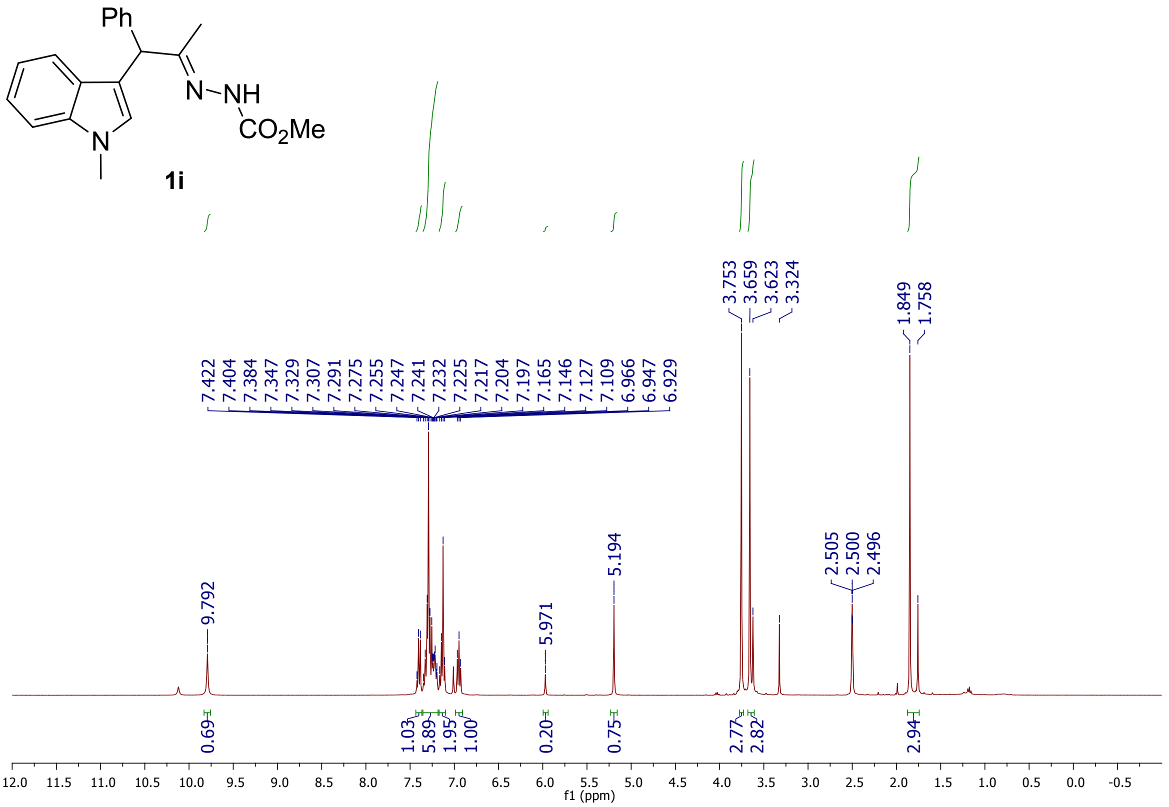

^{13}C{^{1}H} NMR of 1i (100 MHz, DMSO-*d_6*)

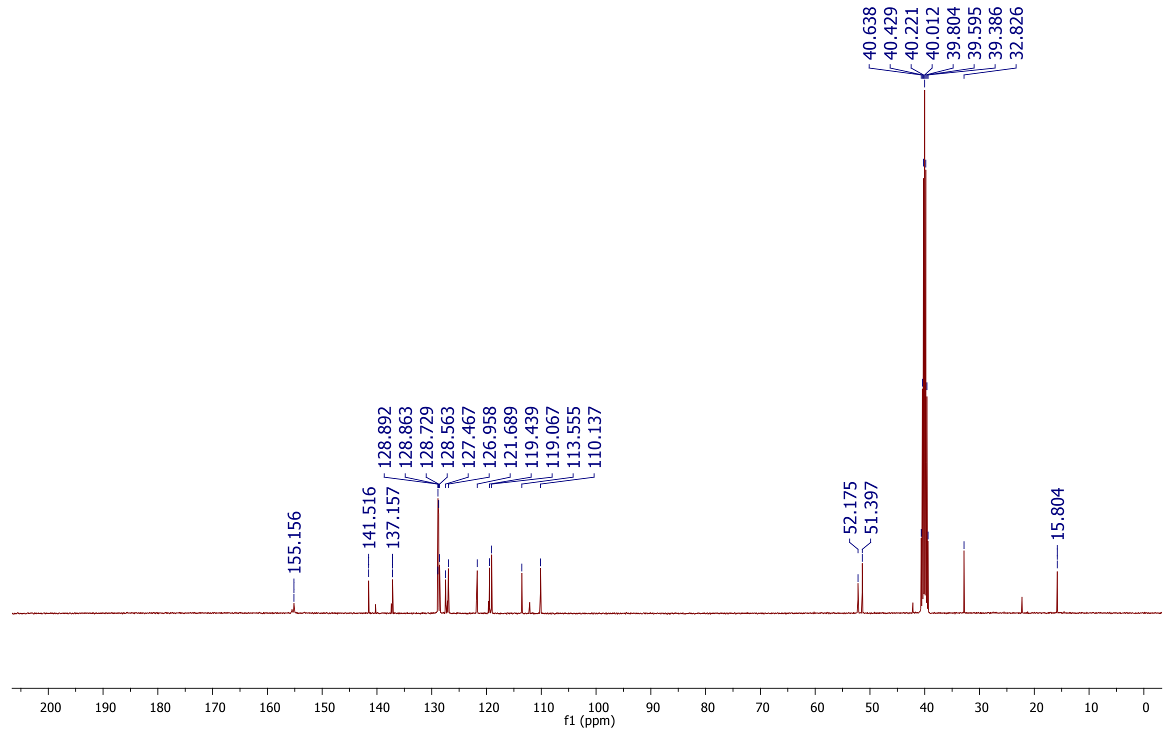

^{1}H NMR of 1j (400 MHz, DMSO-d_6)

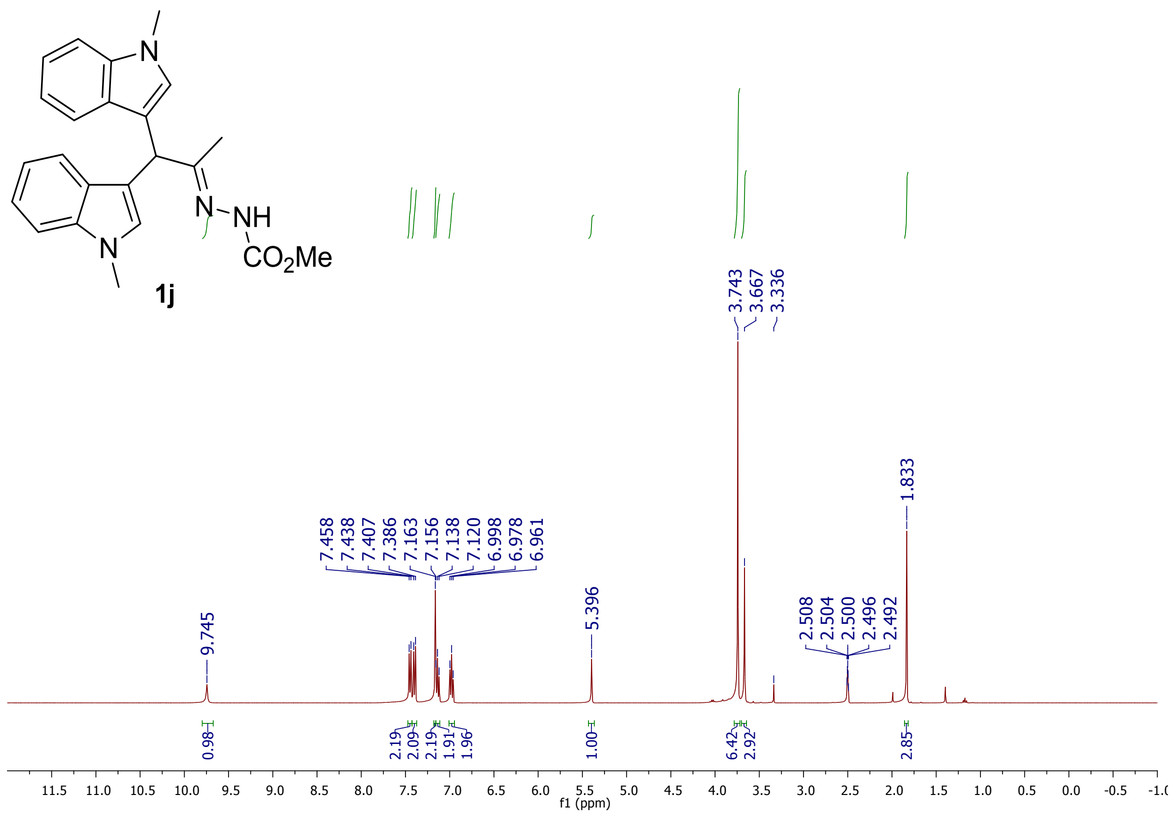

^{13}C{^{1}H} NMR of 1j (100 MHz, DMSO-d_6)

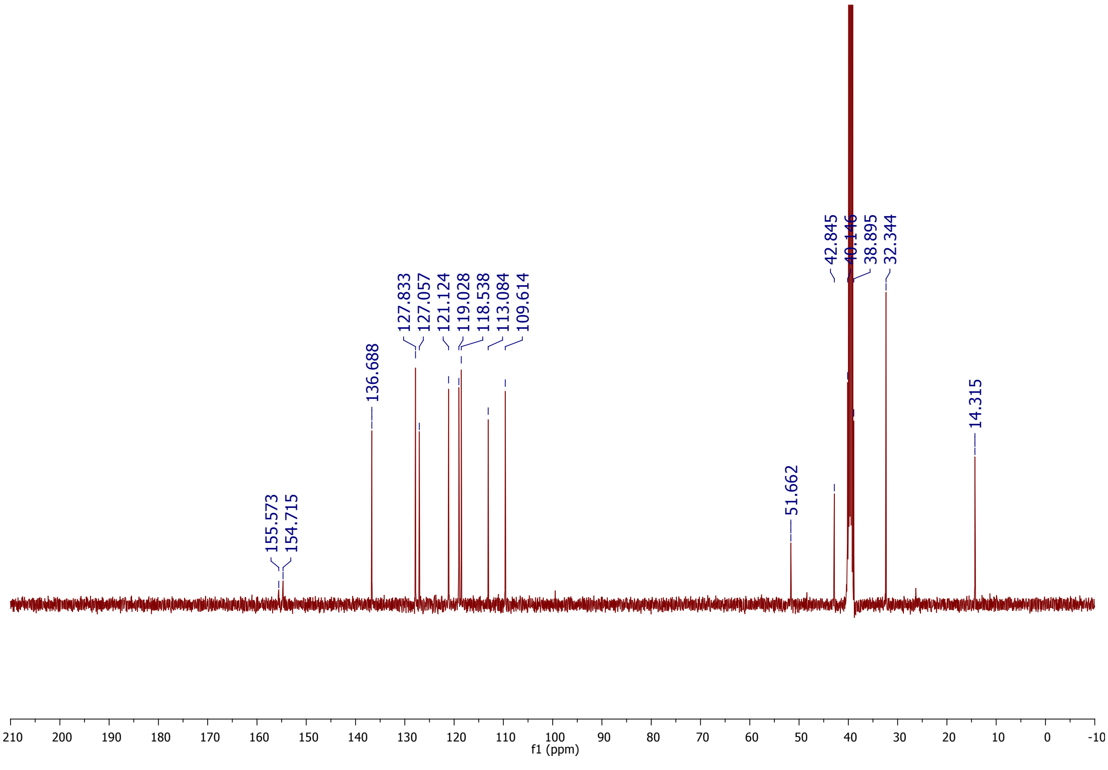

^{1}H NMR of 1k (400 MHz, DMSO-d_6)

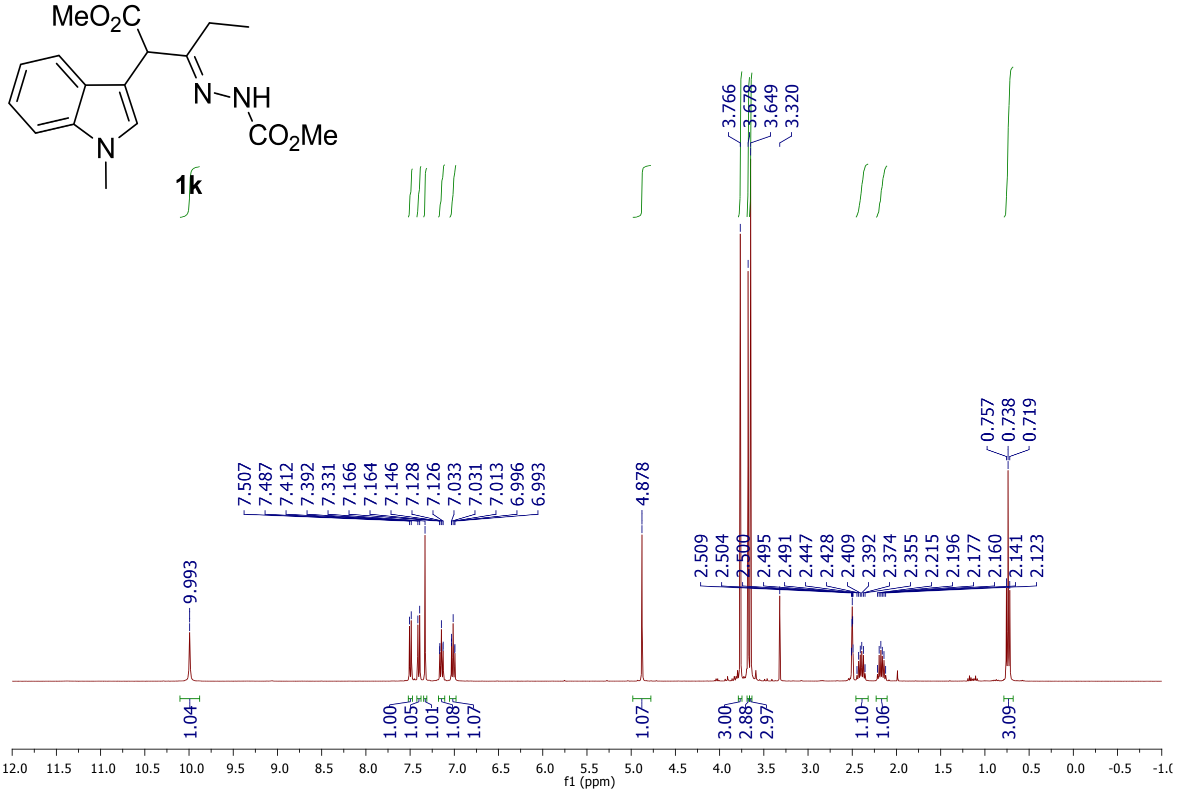

^{13}C{^{1}H} NMR of 1k (100 MHz, DMSO-d_6)

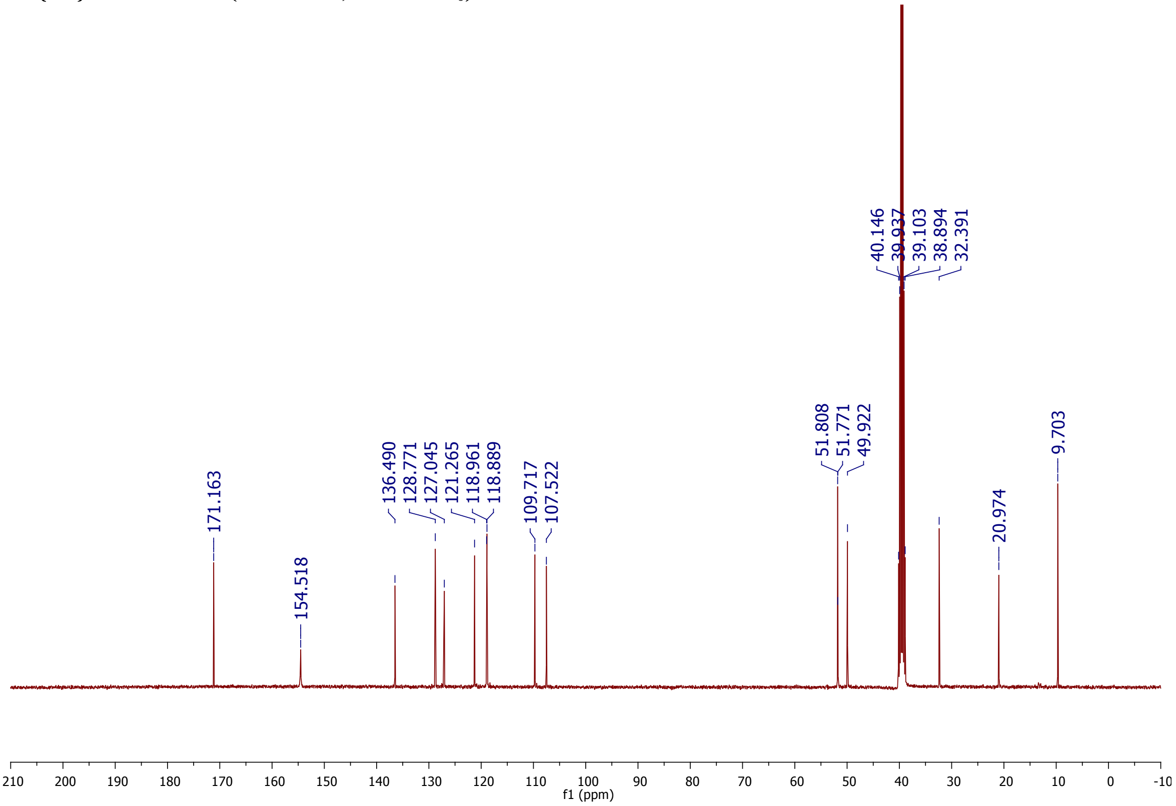

^{1}H NMR of 1l (400 MHz, DMSO-d_6)

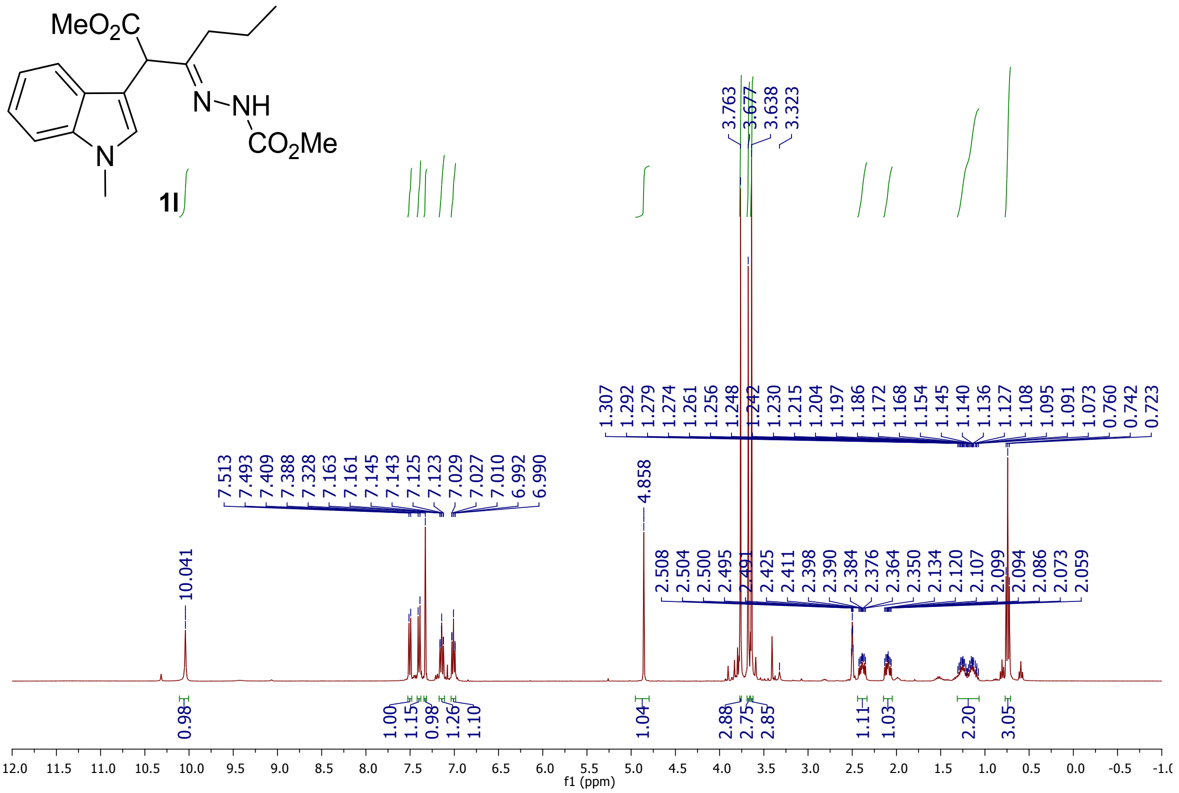

^{13}C{^{1}H} NMR of 1l (100 MHz, DMSO-d_6)

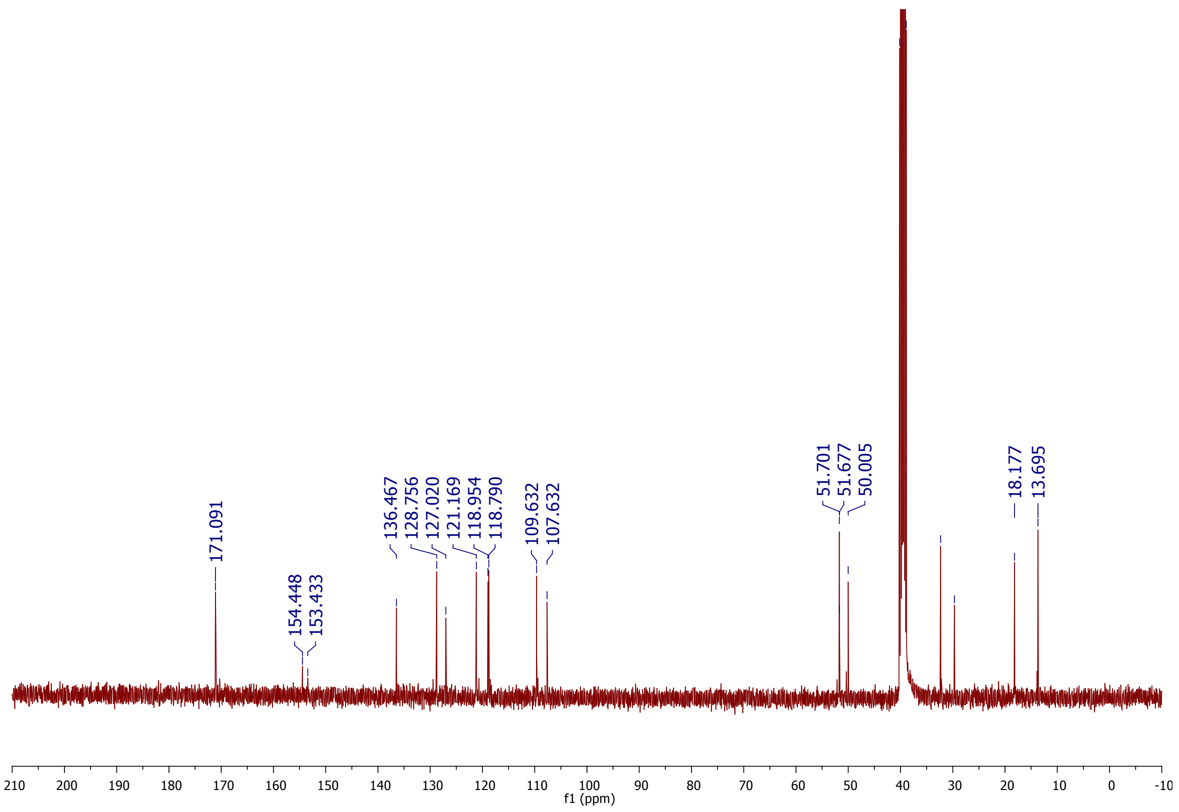

^{1}H NMR of 1m (400 MHz, DMSO-d_6)

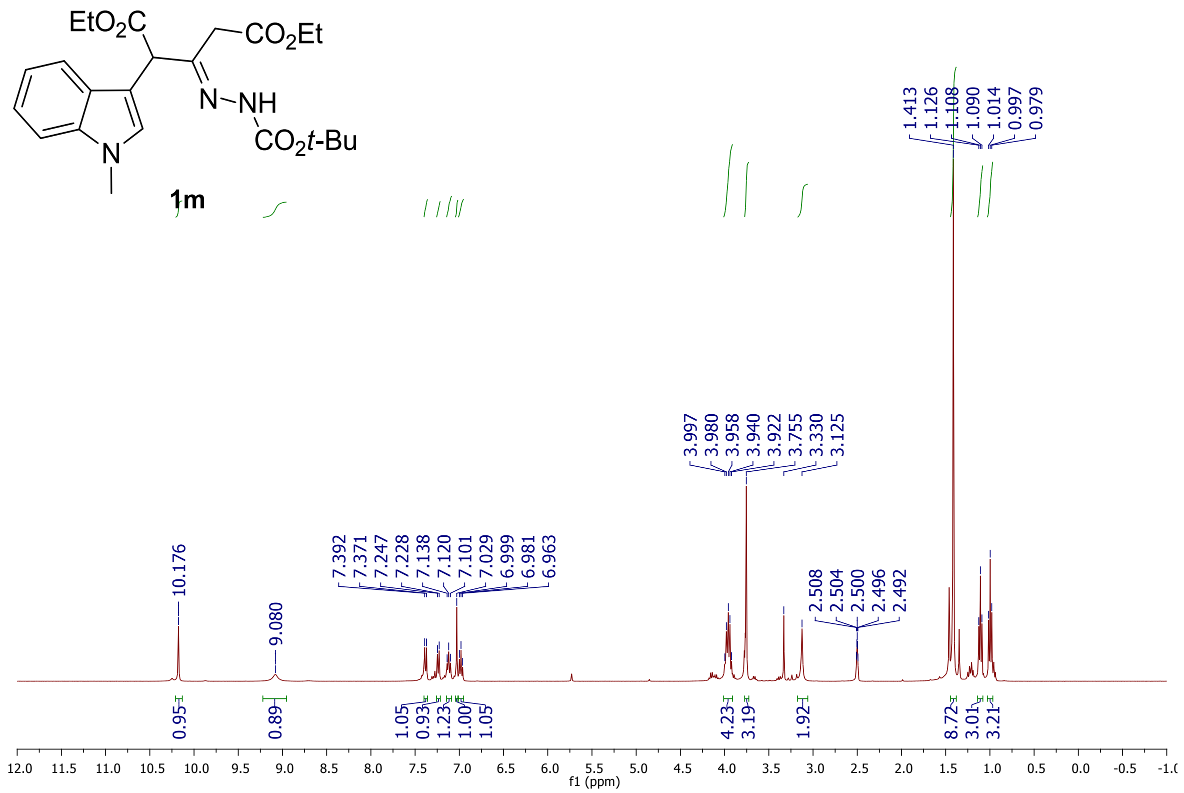

^{13}C{^{1}H} NMR of 1m (100 MHz, DMSO-d_6)

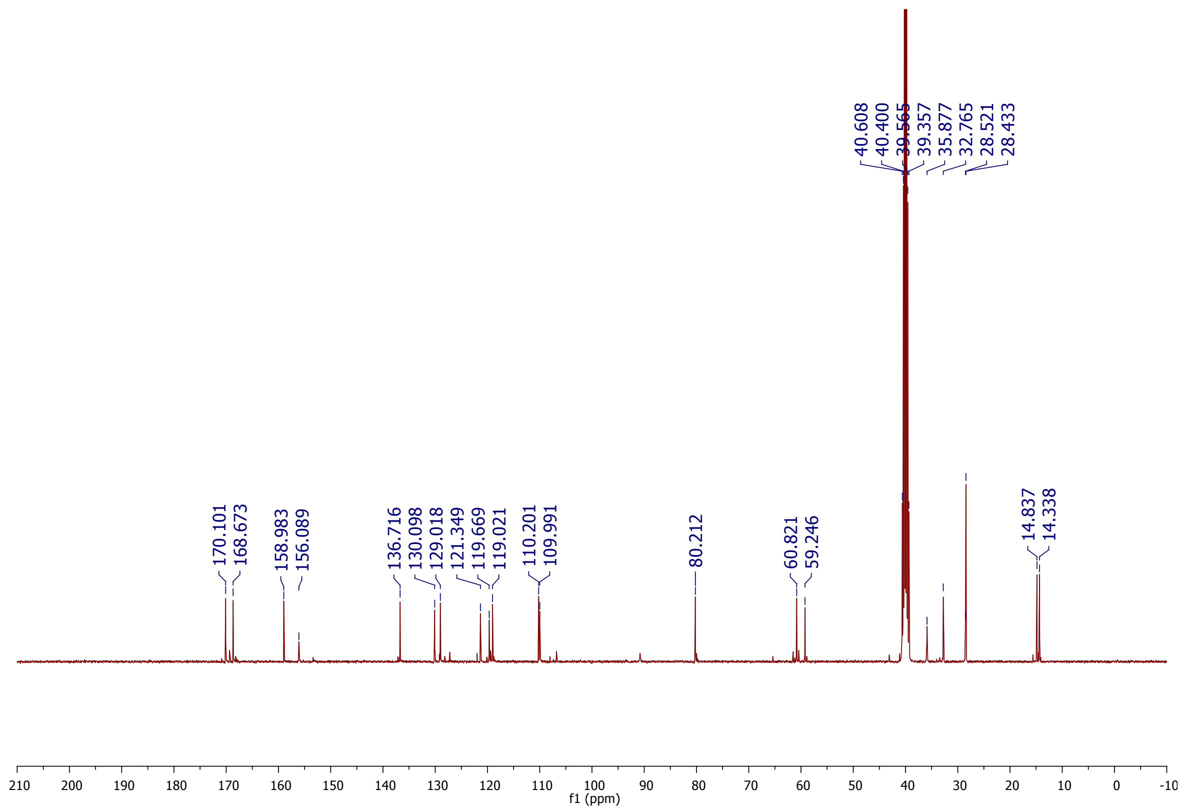

^{1}H NMR of 1n (400 MHz, DMSO-d_6)

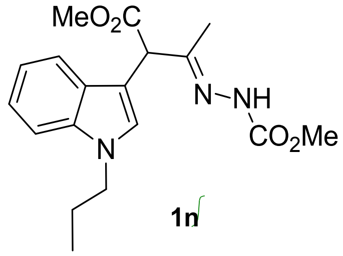

1n

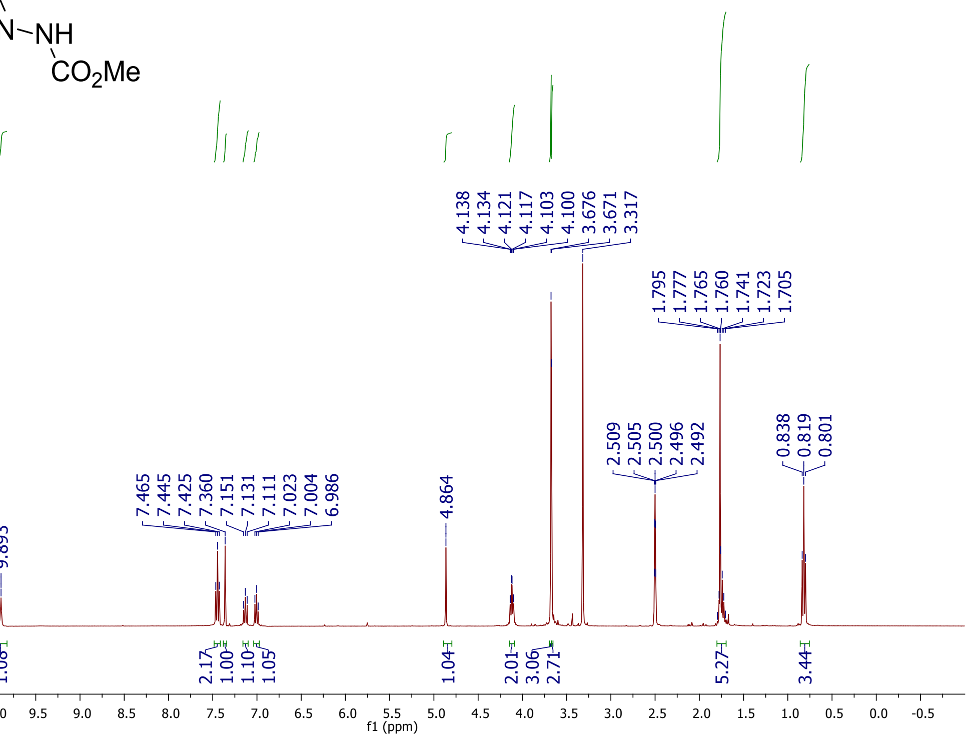

$^{13}C\{^{1}H\}$ NMR of 1n (100 MHz, DMSO-d_6)

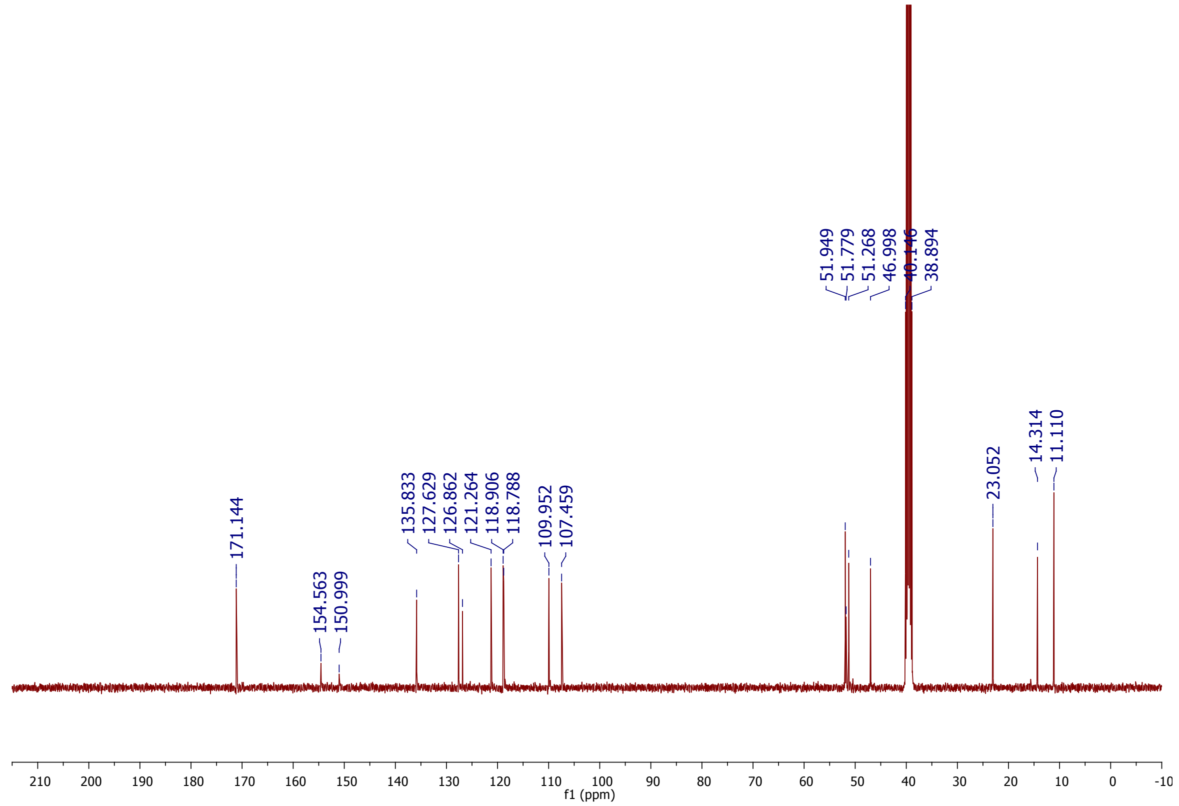

^{1}H NMR of 1o (400 MHz, DMSO-d_6)

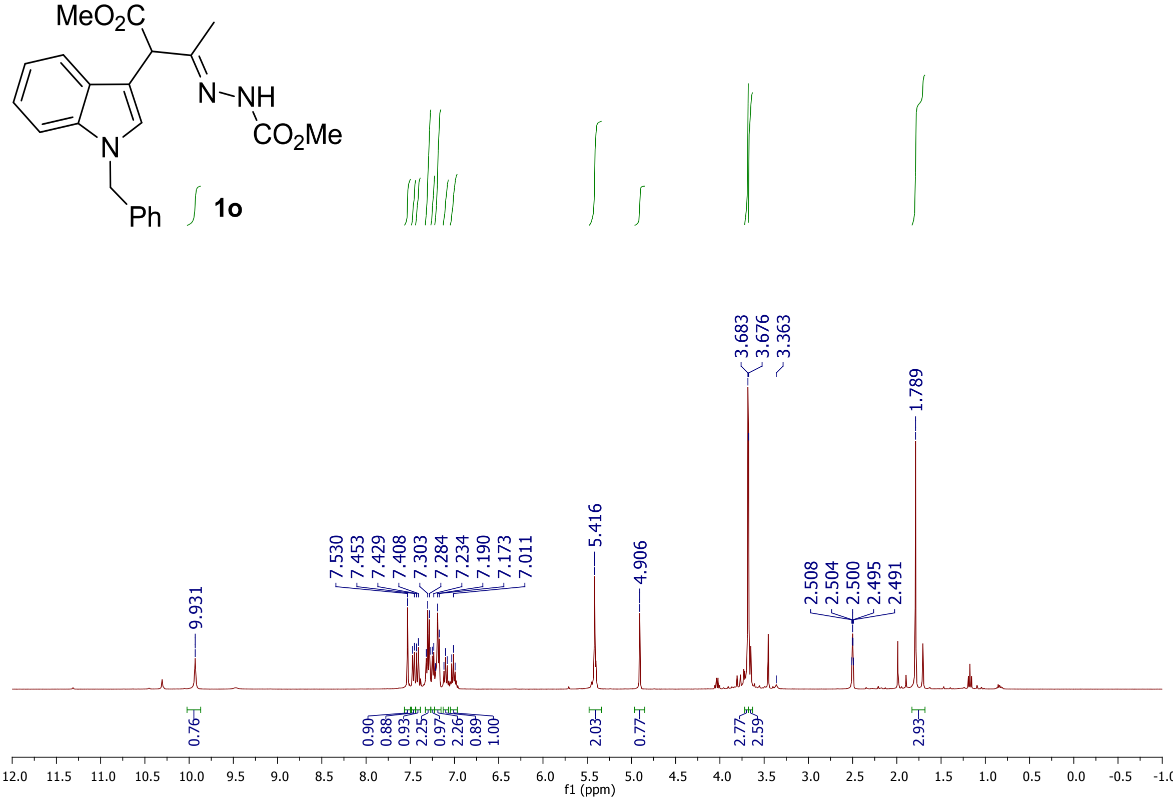

^{13}C{^{1}H} NMR of 1o (100 MHz, DMSO-d_6)

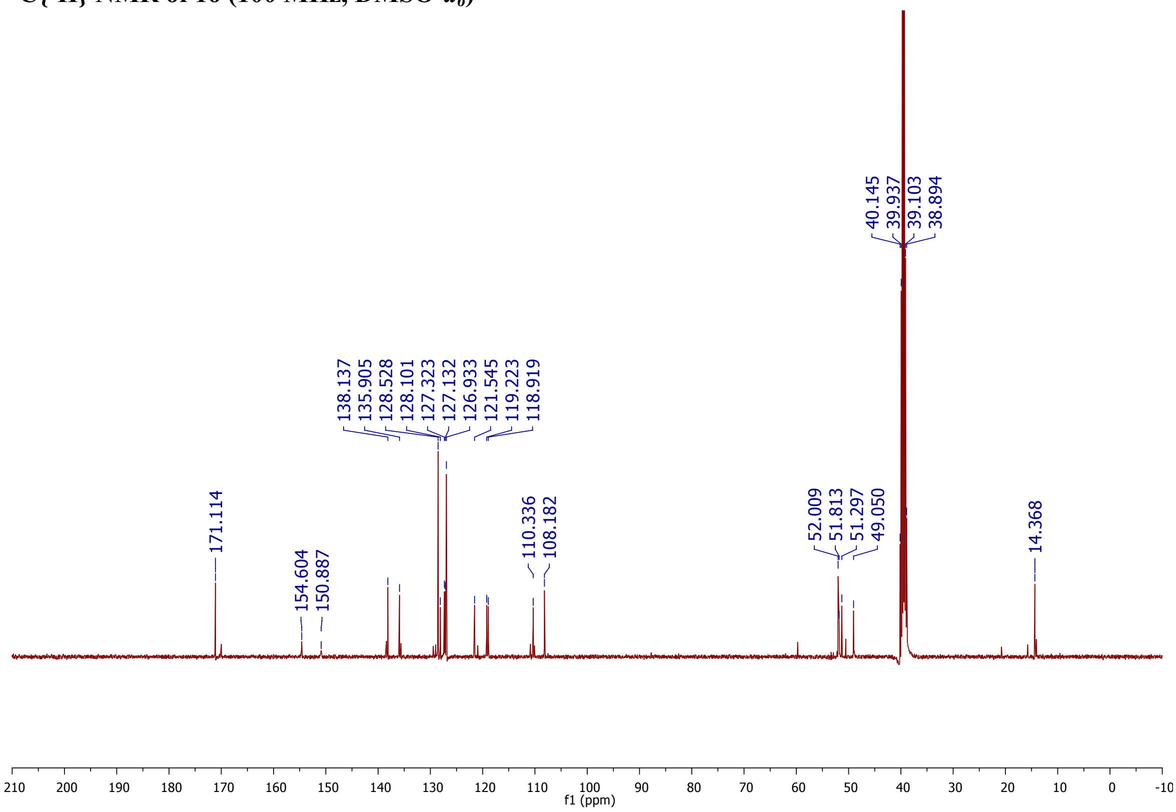

^{1}H NMR of 1p (400 MHz, DMSO-d_6)

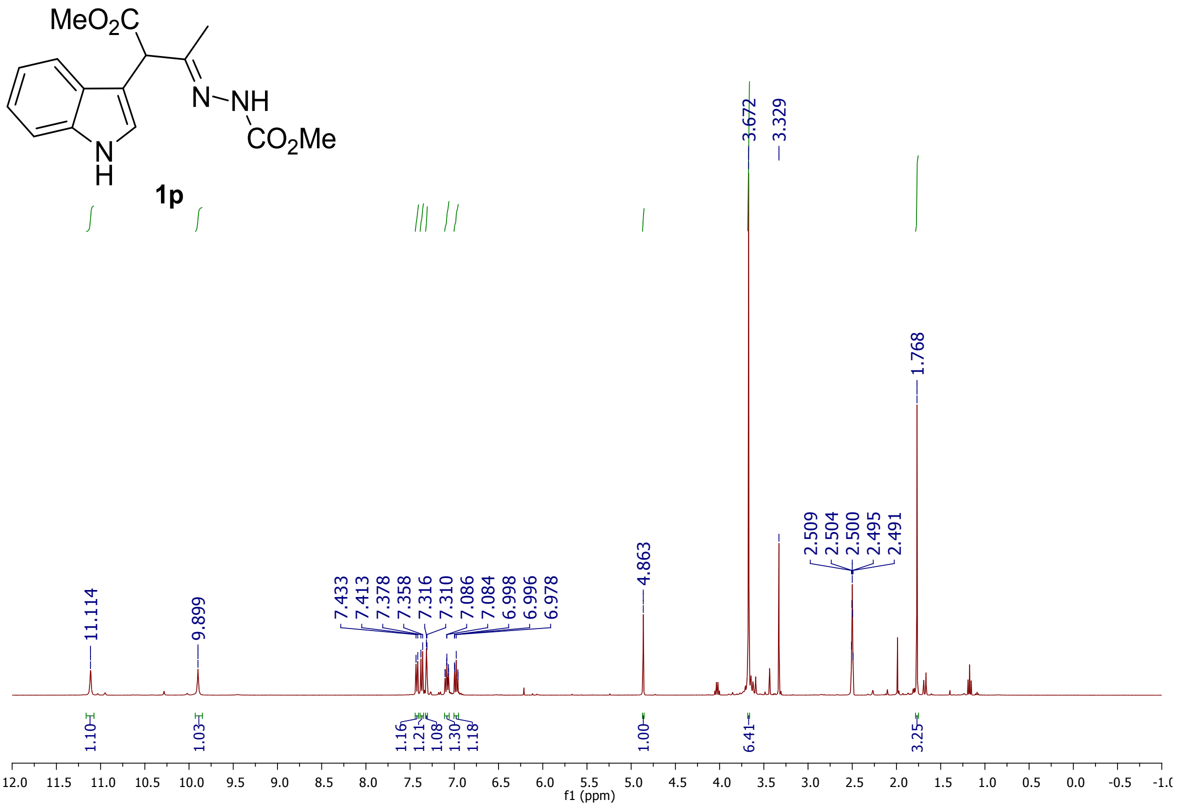

^{13}C{^{1}H} NMR of 1p (100 MHz, DMSO-d_6)

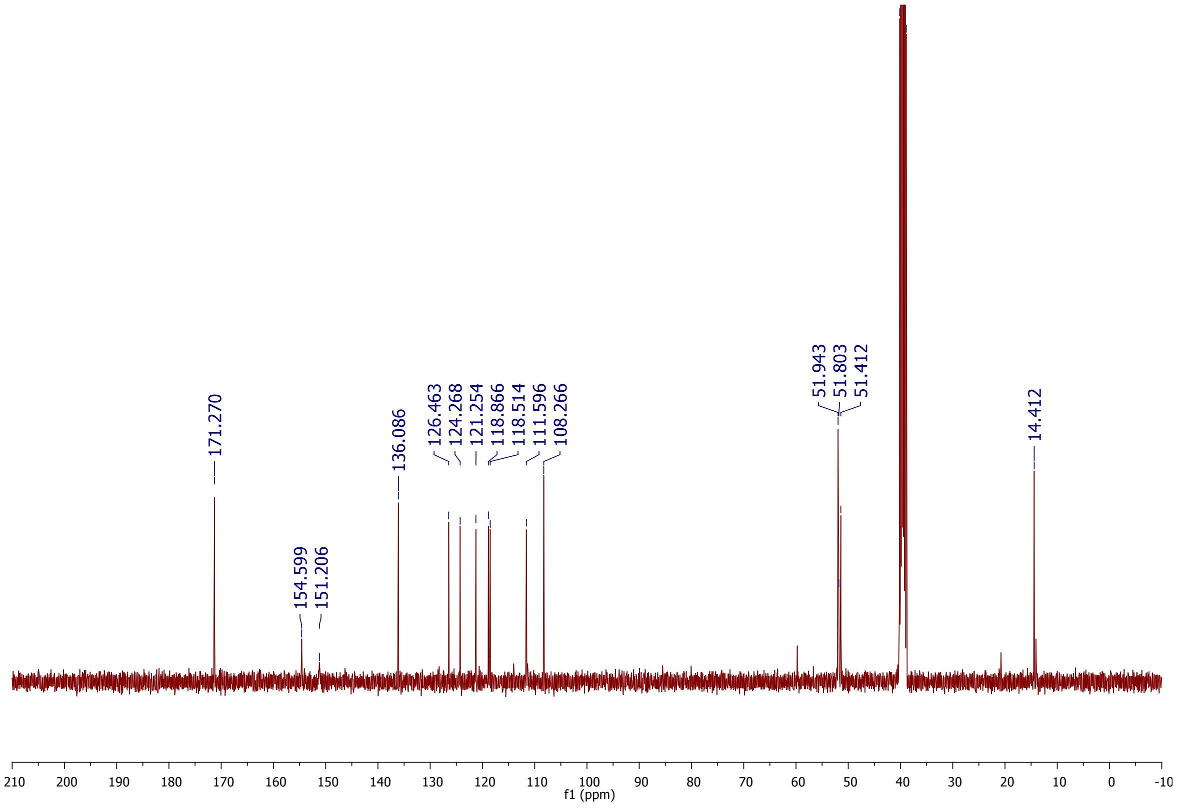

^{1}H NMR of 1q (400 MHz, DMSO-d_6)

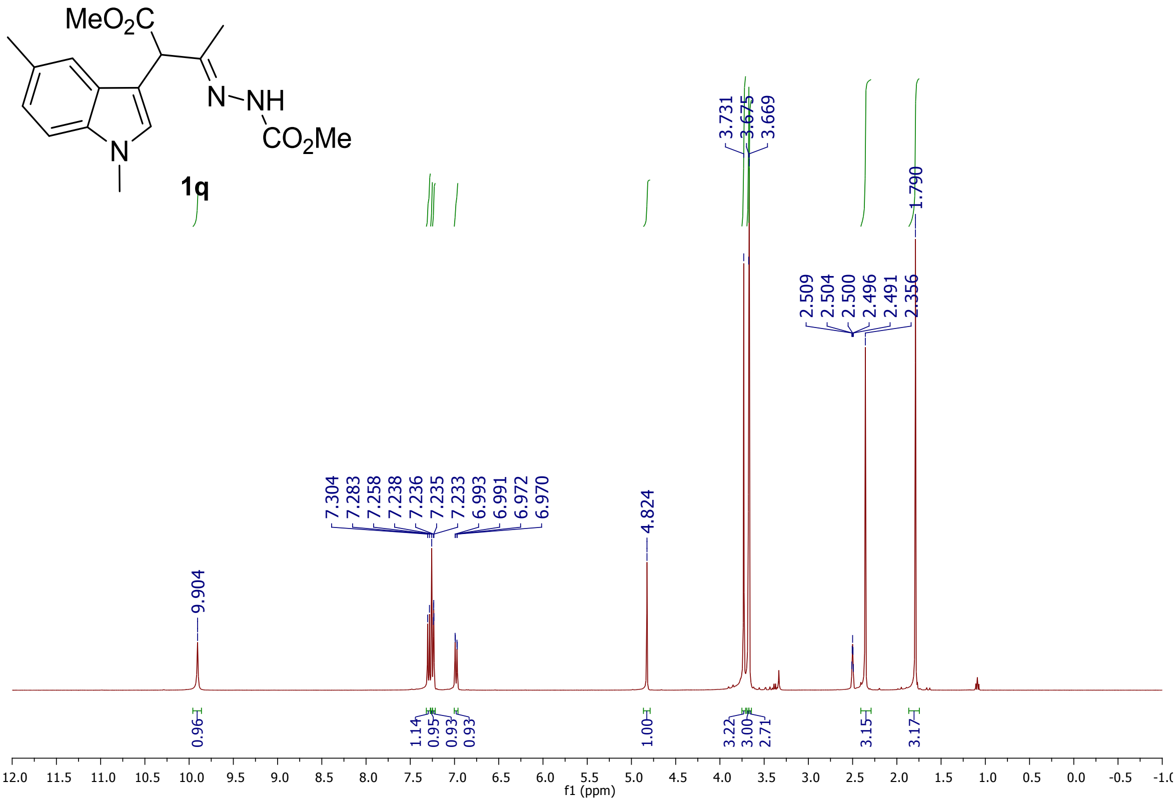

^{13}C{^{1}H} NMR of 1q (100 MHz, DMSO-d_6)

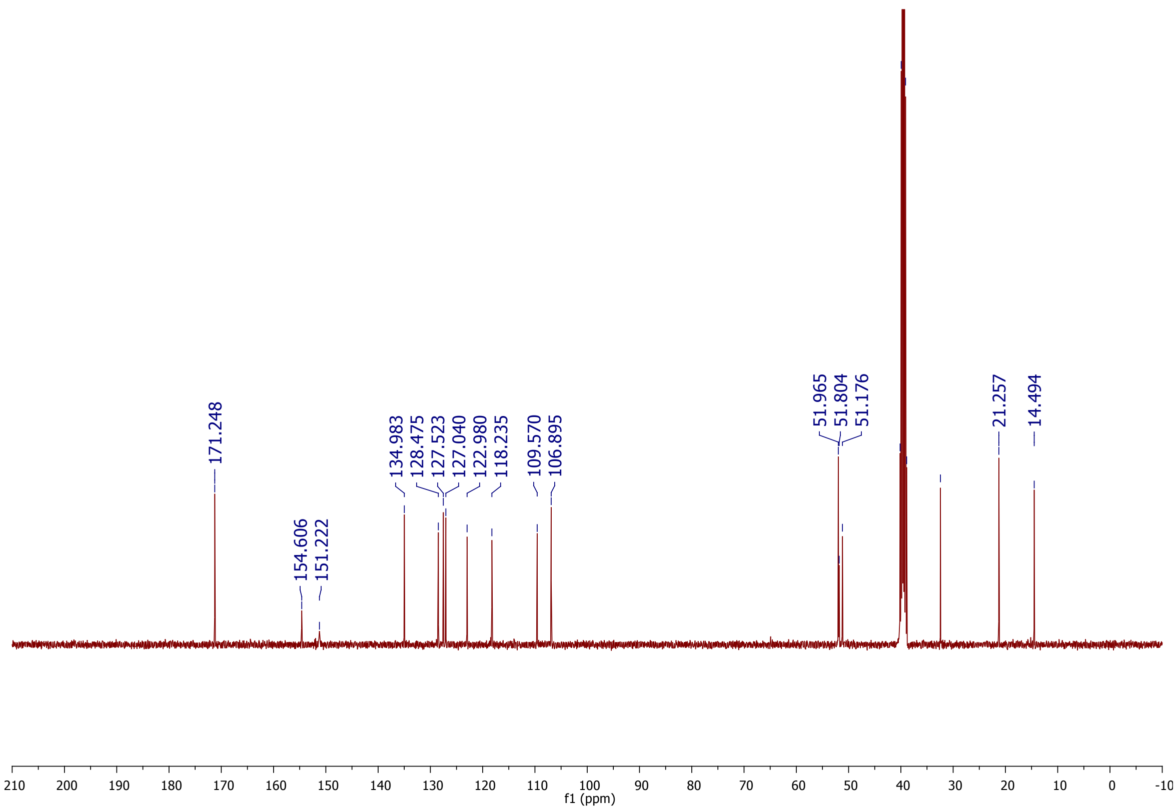

^{1}H NMR of 1r (400 MHz, DMSO-d_6)

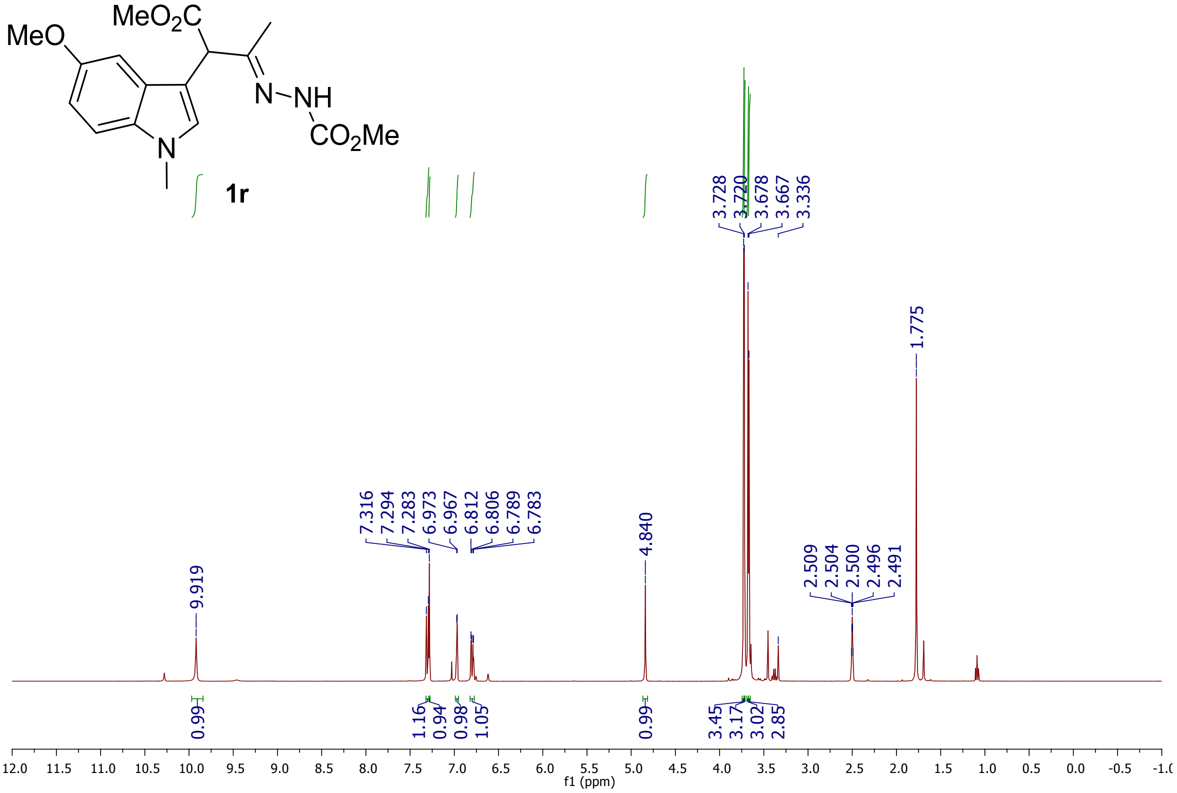

^{13}C{^{1}H} NMR of 1r (100 MHz, DMSO-d_6)

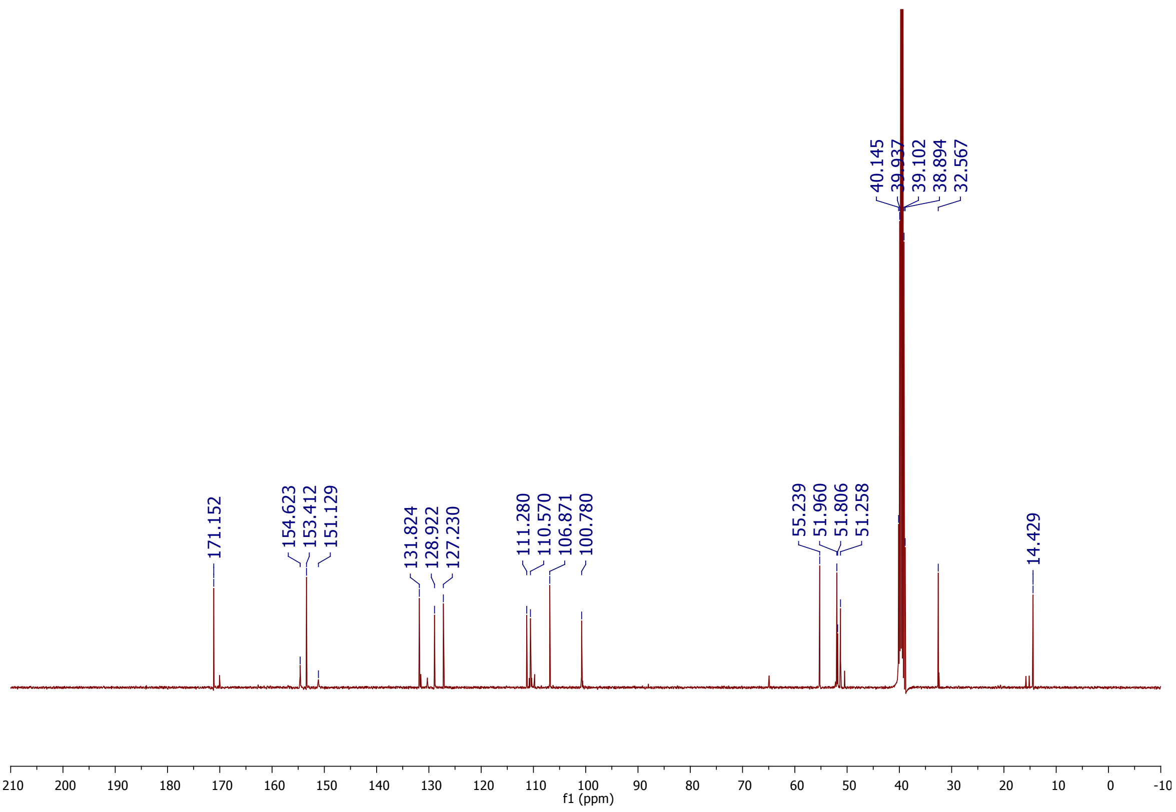

^{1}H NMR of 1s (400 MHz, DMSO-d_6)

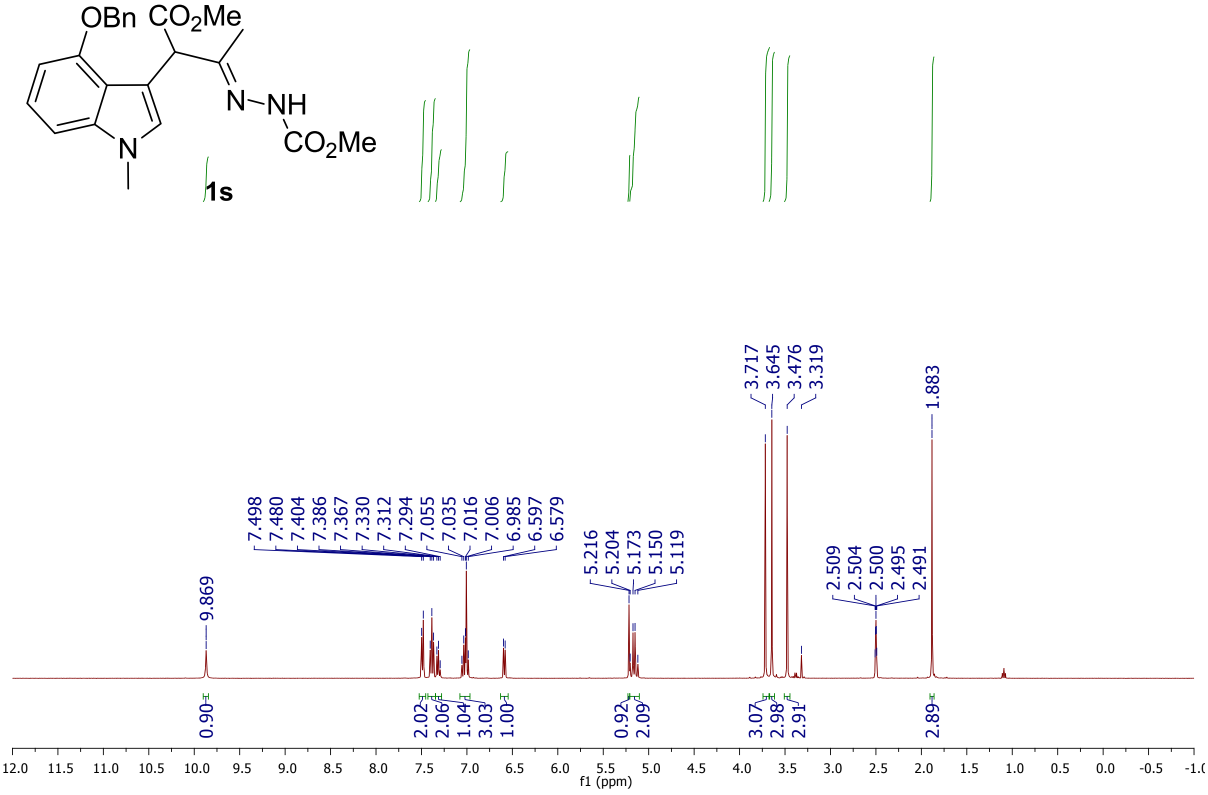

^{13}C{^{1}H} NMR of 1s (100 MHz, DMSO-d_6)

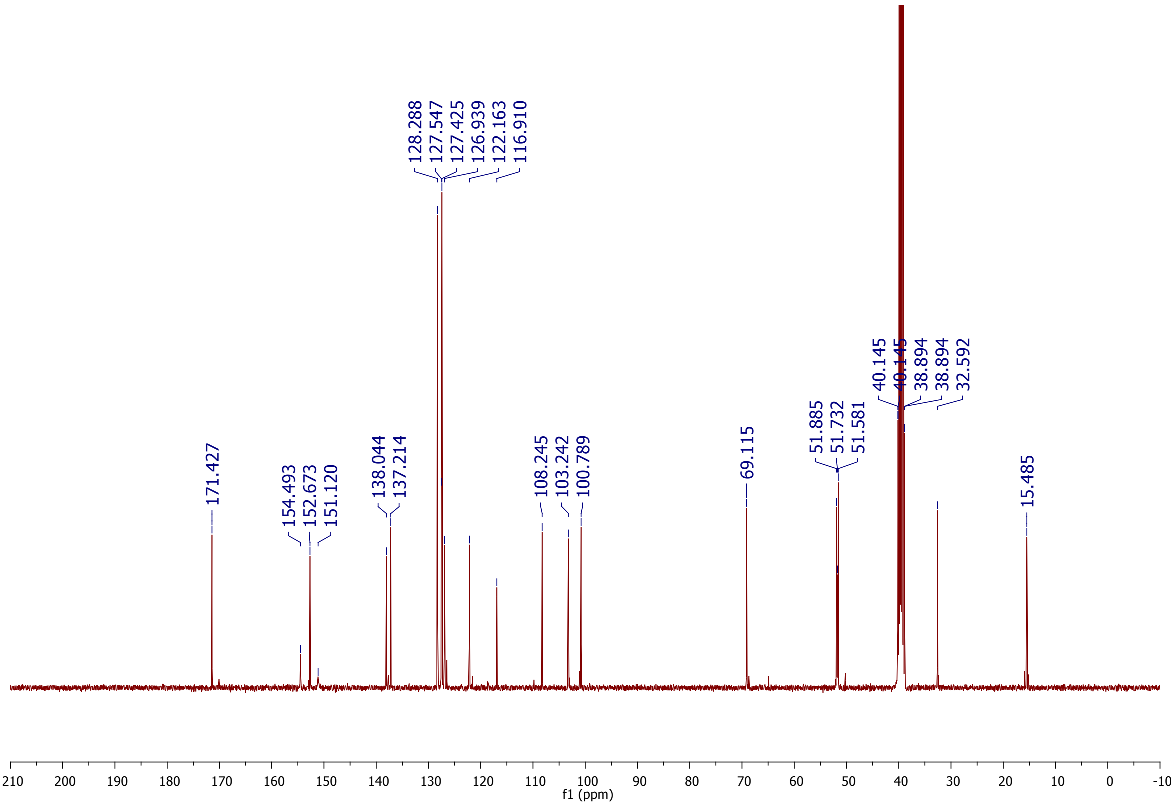

^{1}H NMR of 1t (400 MHz, DMSO-d_6)

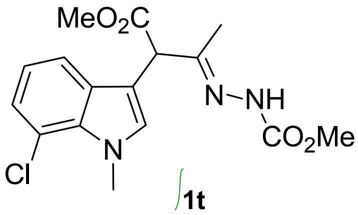

1t

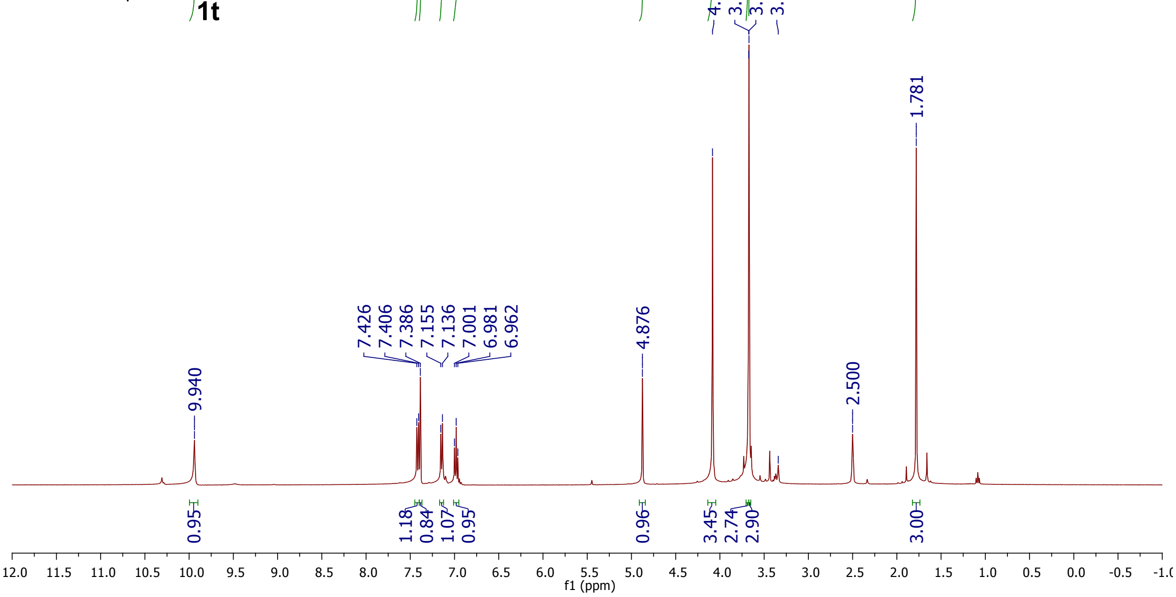

^{13}C{^{1}H} NMR of 1t (100 MHz, DMSO-d_6)

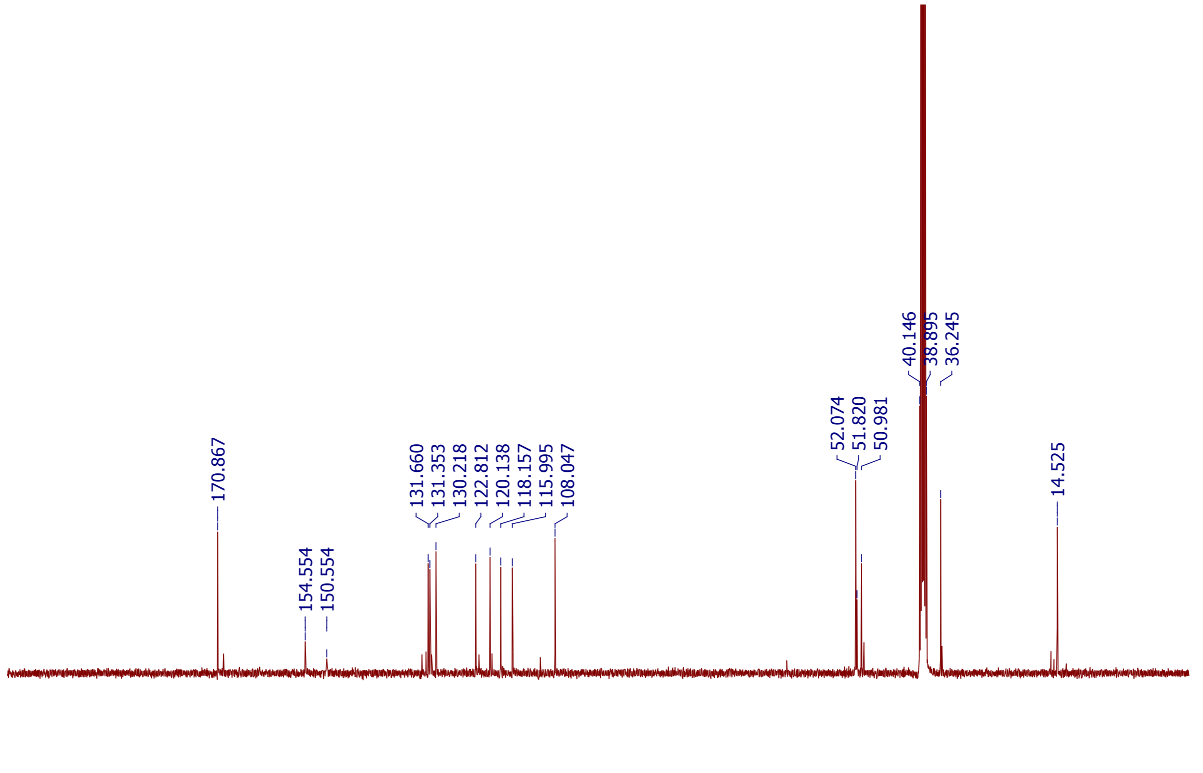

^{1}H NMR of 1u (400 MHz, DMSO-d_6)

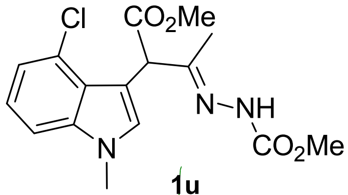

1u

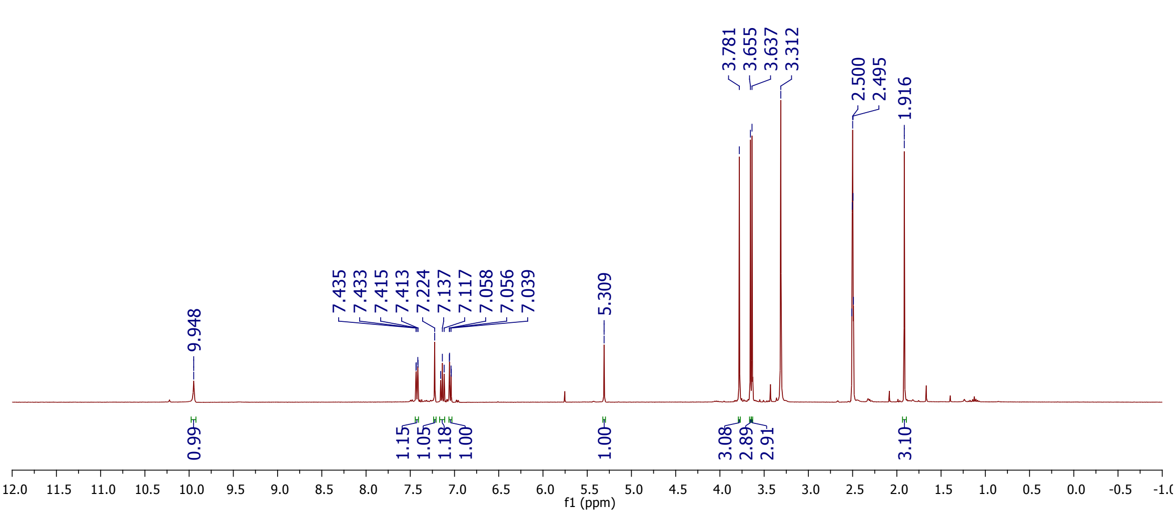

^{13}C{^{1}H} NMR of 1u (100 MHz, DMSO-d_6)

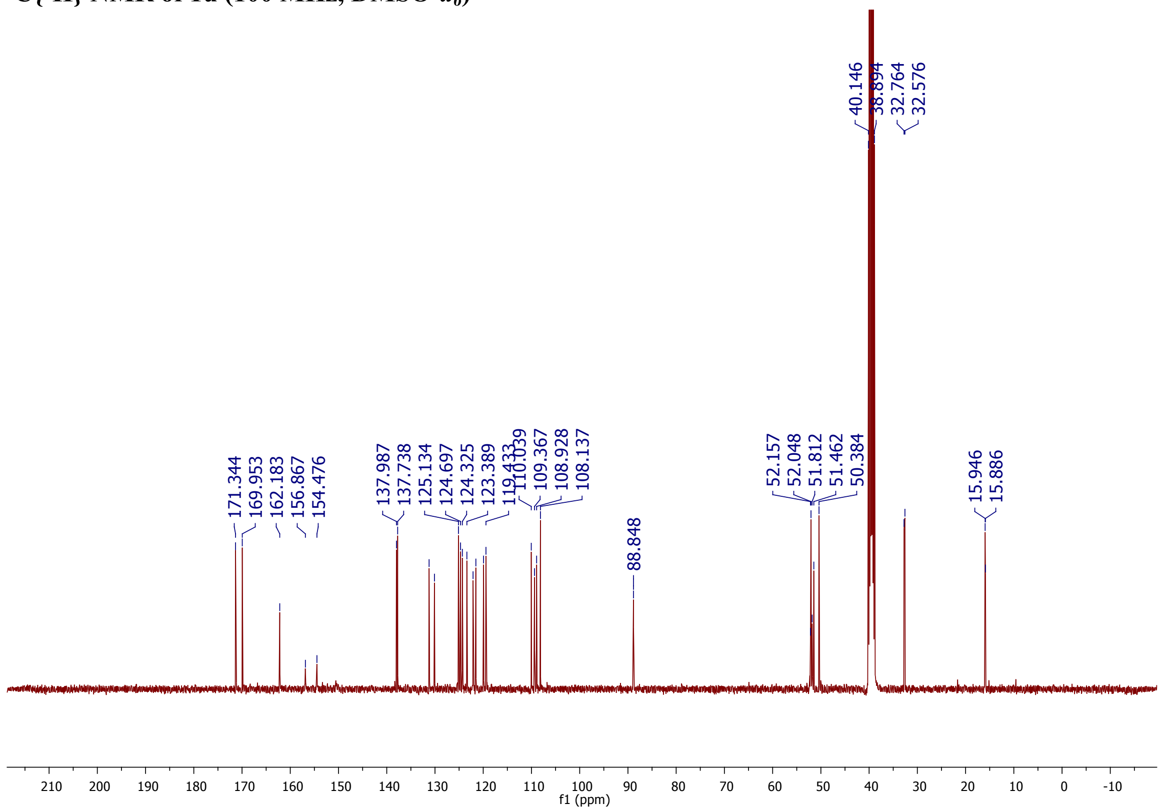

^{1}H NMR of 1v (400 MHz, DMSO-d_6)

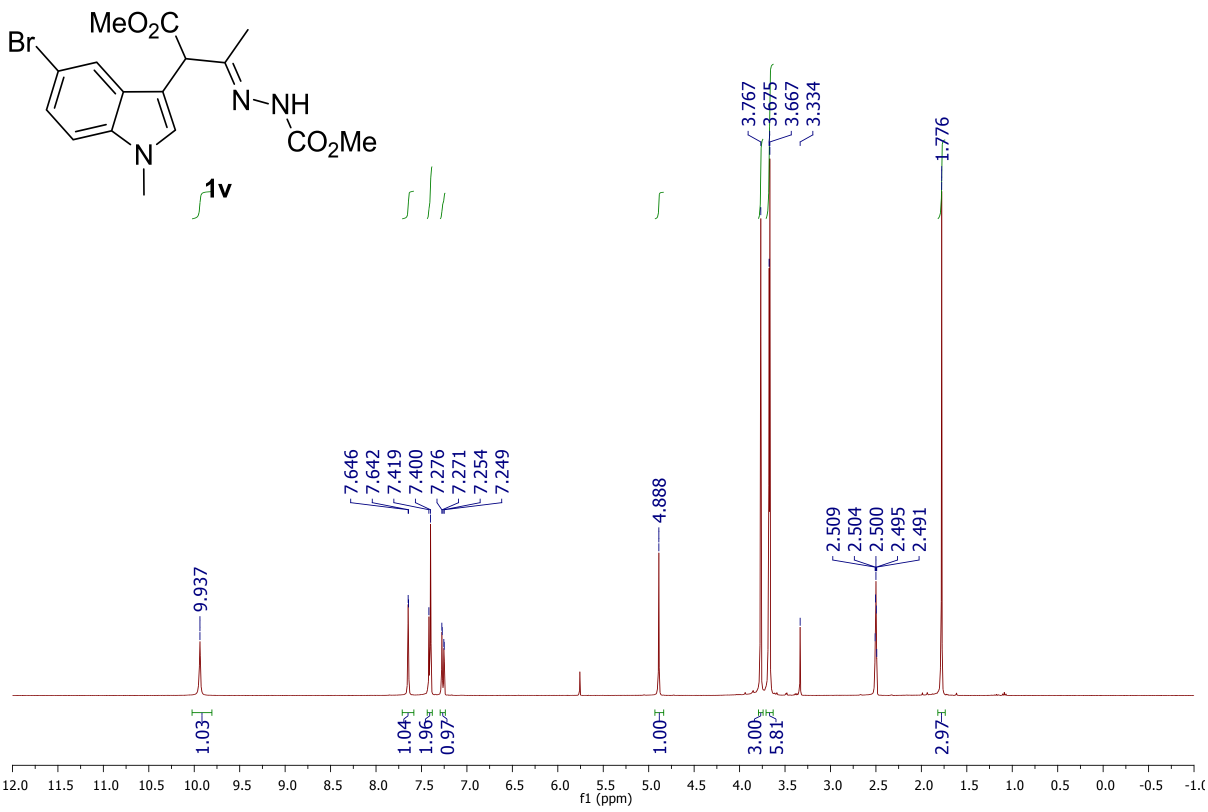

^{13}C{^{1}H} NMR of 1v (100 MHz, DMSO-d_6)

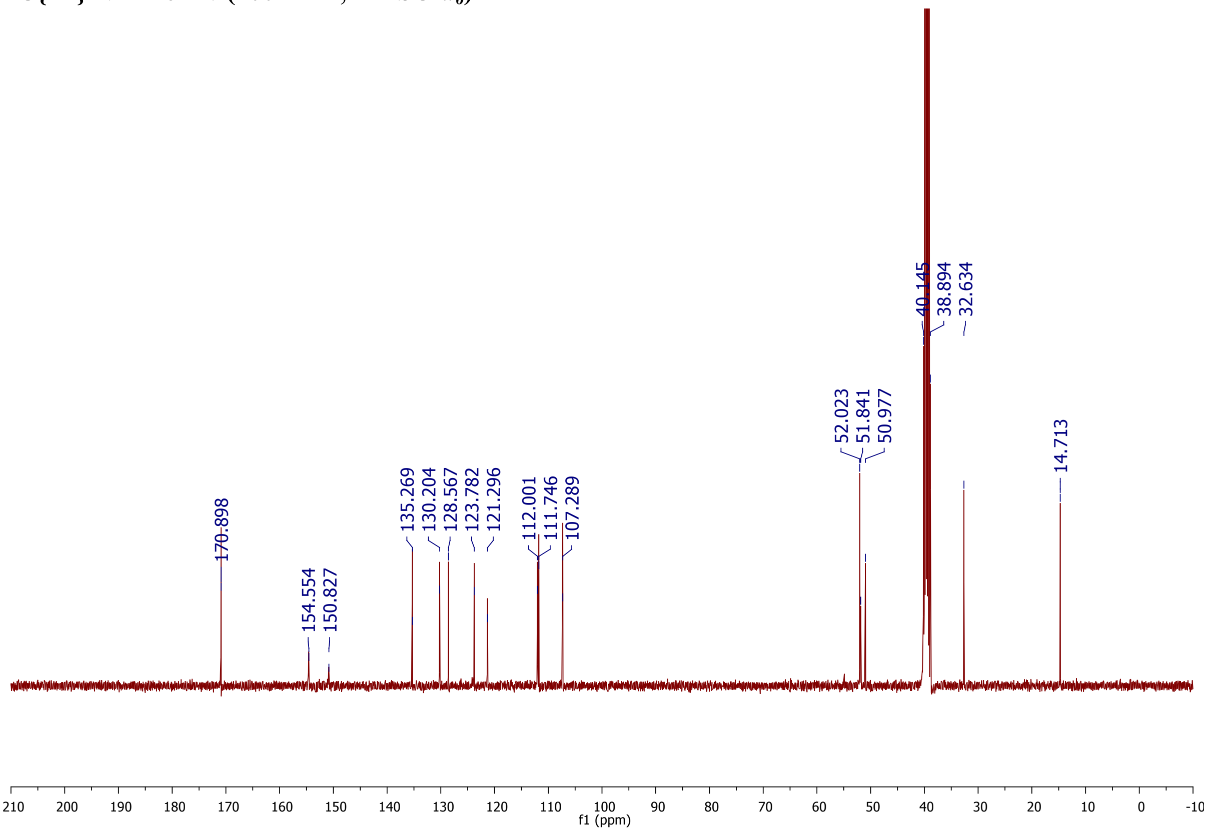

^{1}H NMR of 1w (400 MHz, DMSO-d_6)

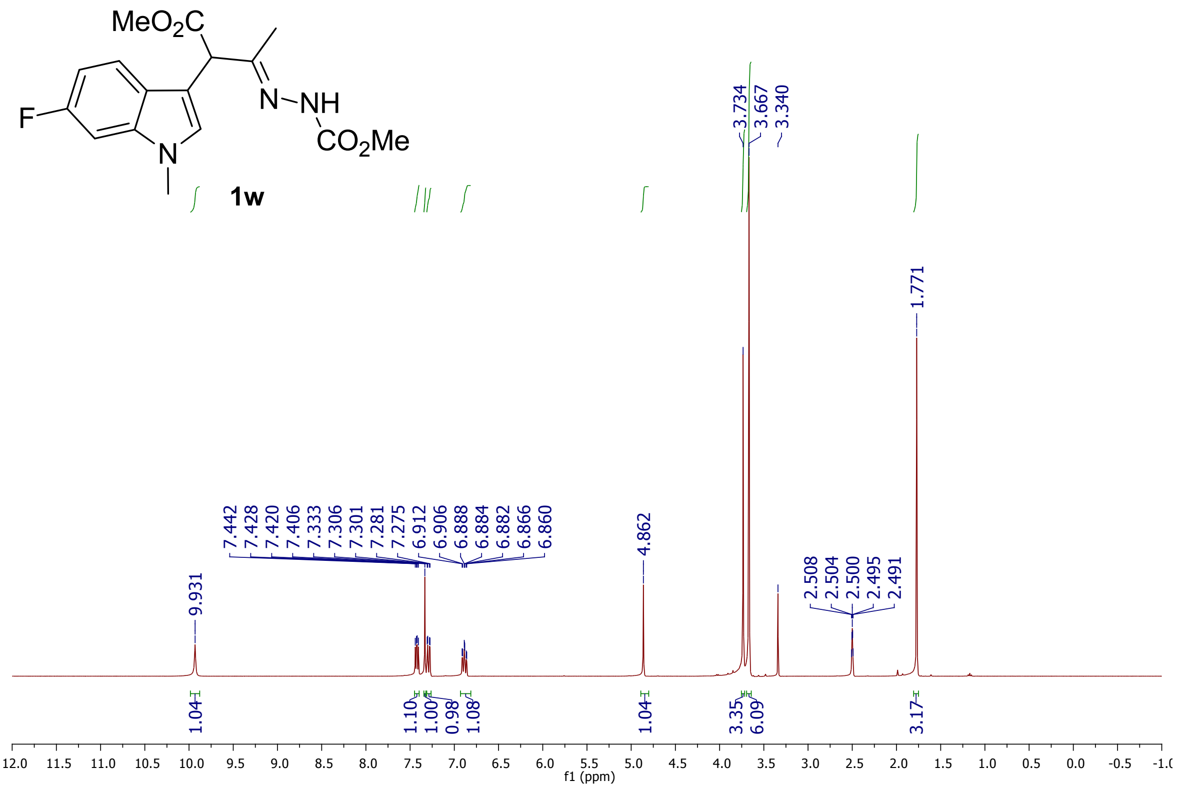

^{13}C{^{1}H} NMR of 1w (100 MHz, DMSO-d_6)

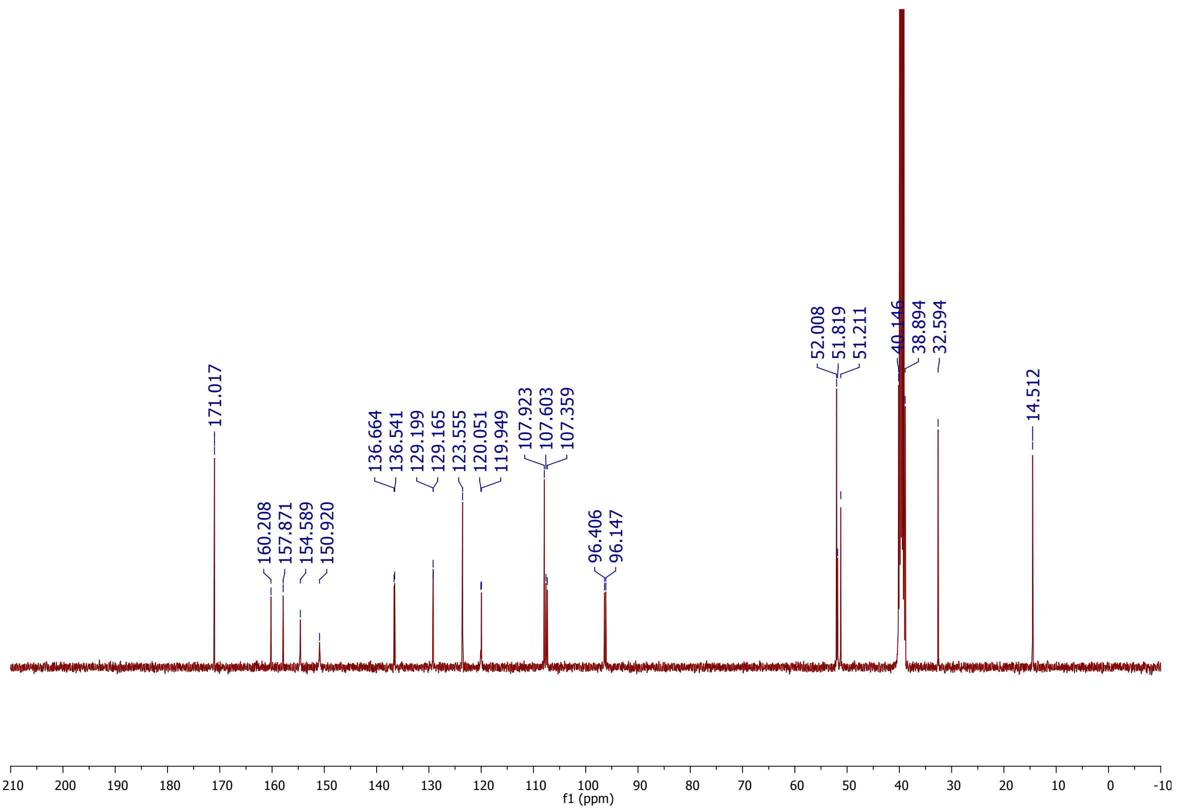

^{1}H NMR of 1x (400 MHz, DMSO-d_6)

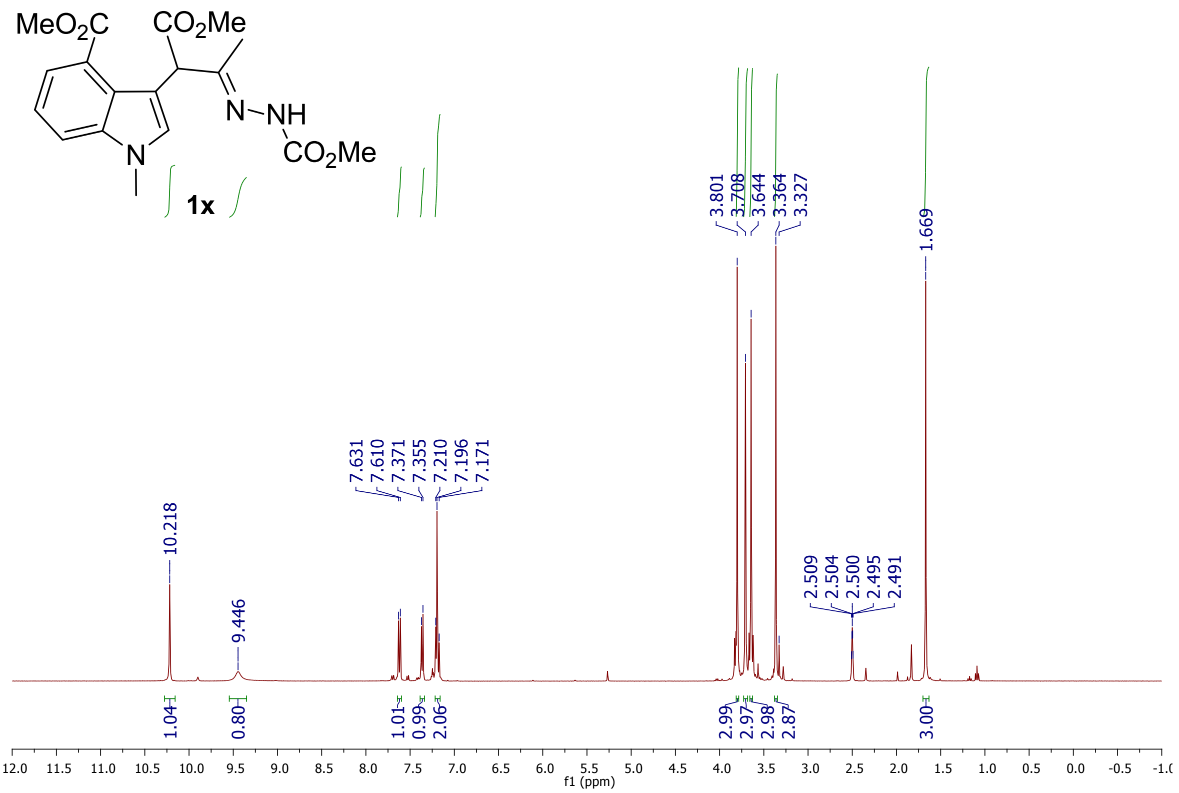

$^{13}C\{^{1}H\}$ NMR of 1x (100 MHz, DMSO-d_6)

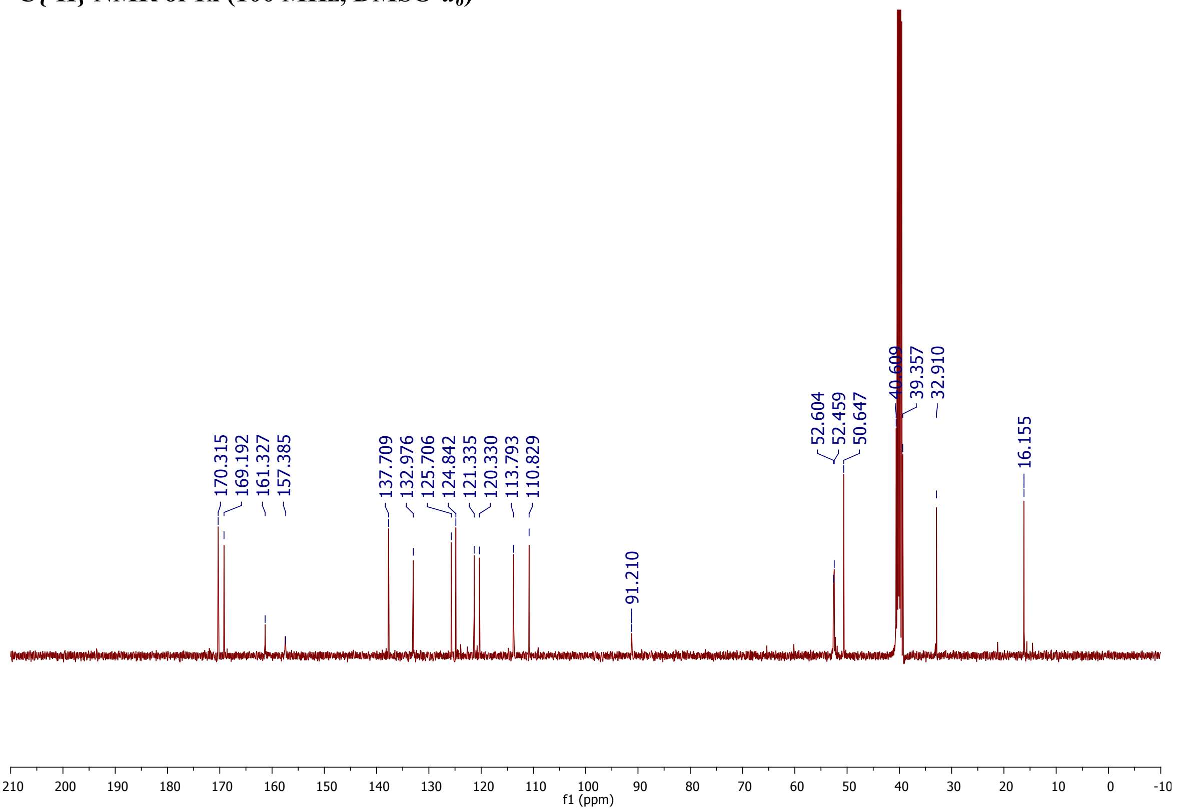

^{1}H NMR of 1y (400 MHz, DMSO-d_6)

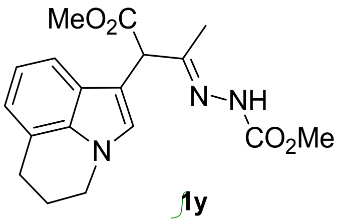

1y

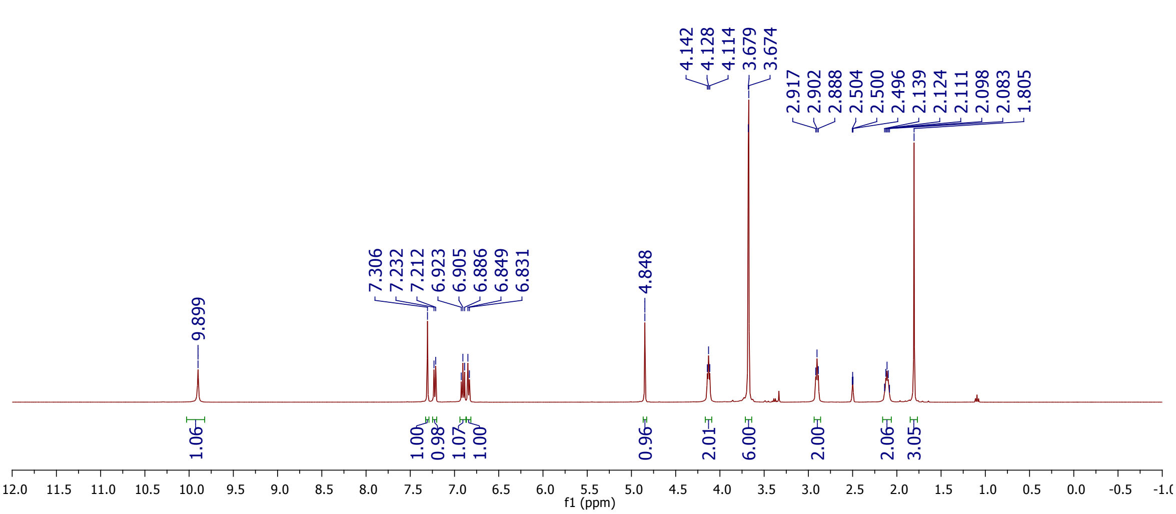

$^{13}C\{^{1}H\}$ NMR of 1y (100 MHz, DMSO-d_6)

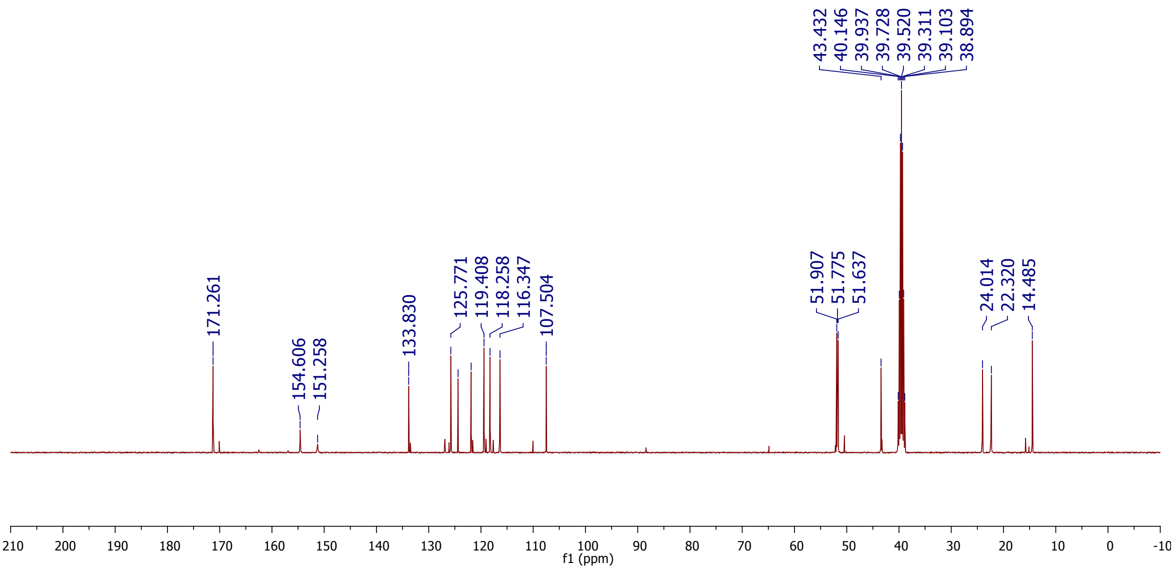

^{1}H NMR of 2a (400 MHz, DMSO-d_6)

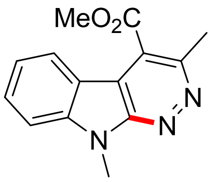

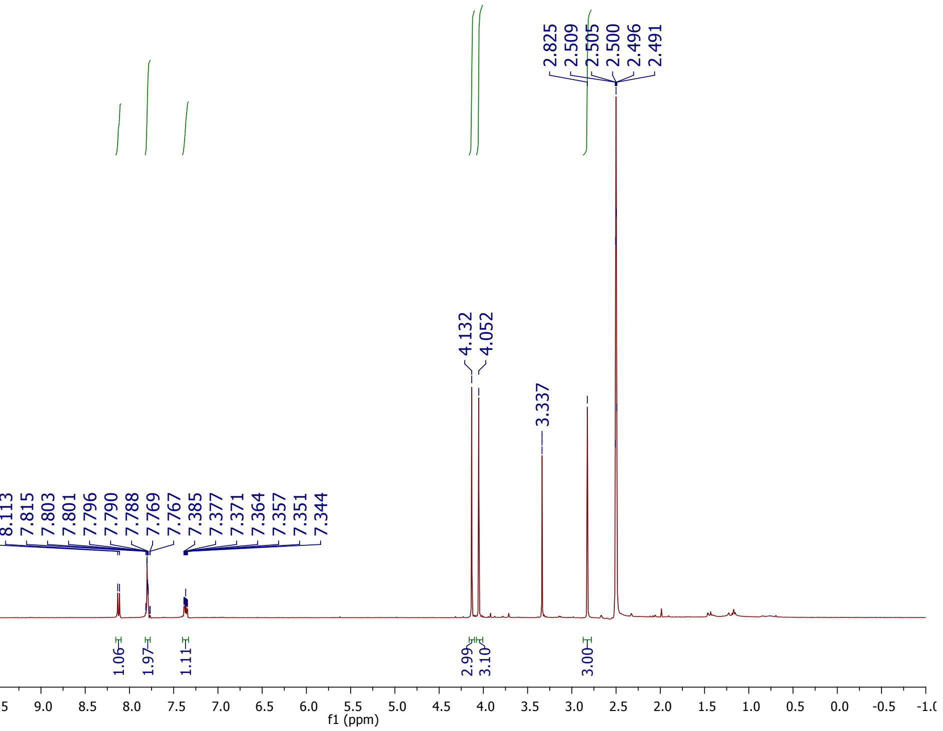

$^{13}C\{^{1}H\}$ NMR of 2a (100 MHz, DMSO-d_6)

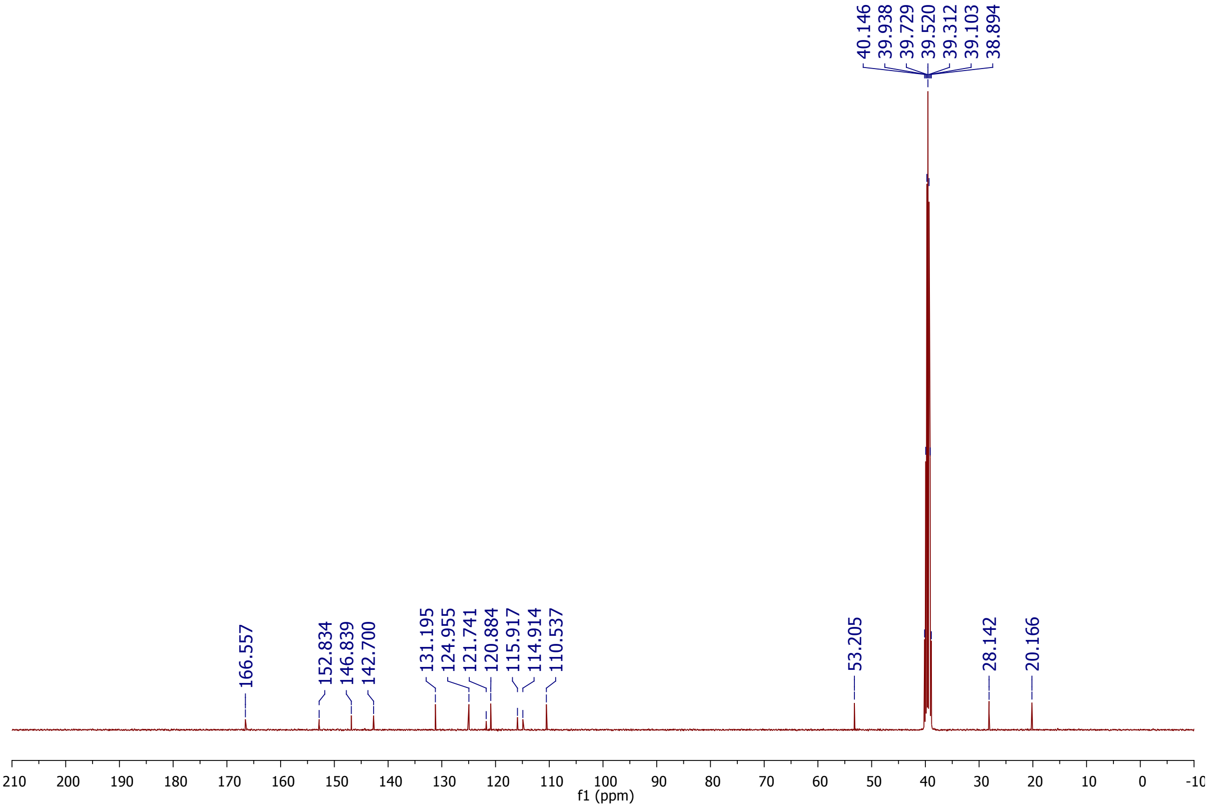

^{1}H NMR of 2b (400 MHz, DMSO-d_6)

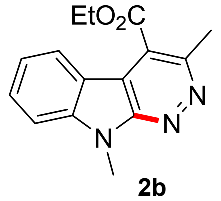

2b

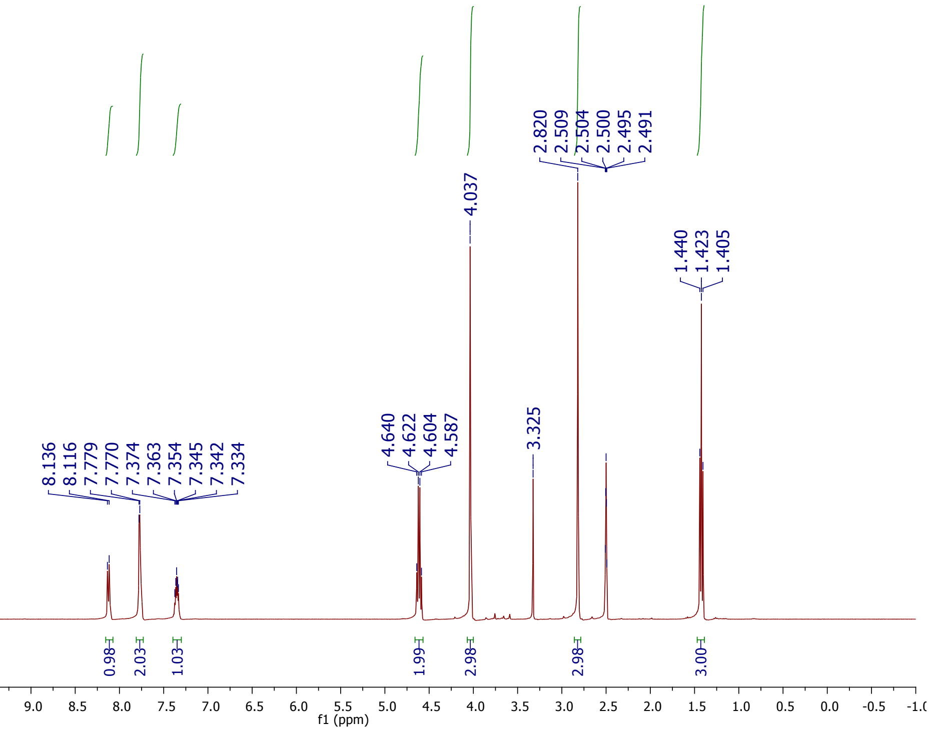

^{13}C{^{1}H} NMR of 2b (100 MHz, DMSO-d_6)

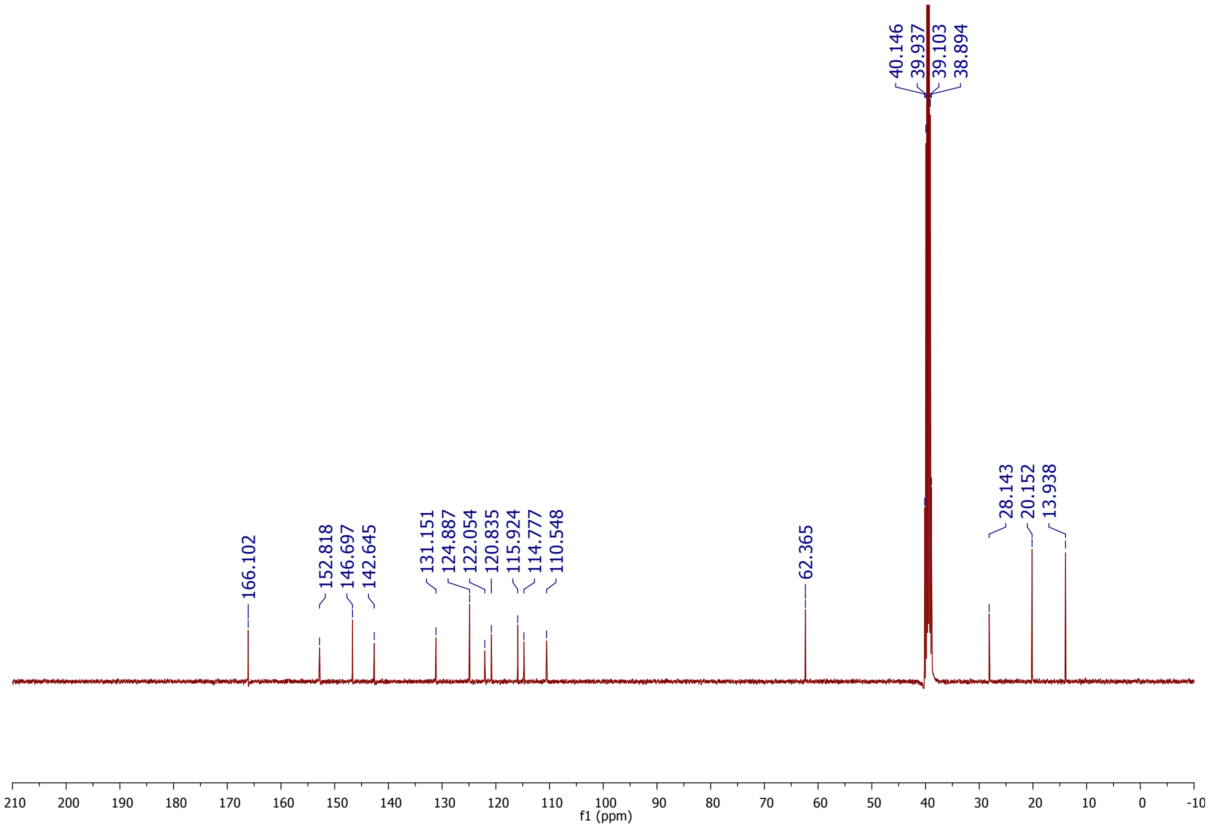

^{1}H NMR of 2c (400 MHz, DMSO-d_6)

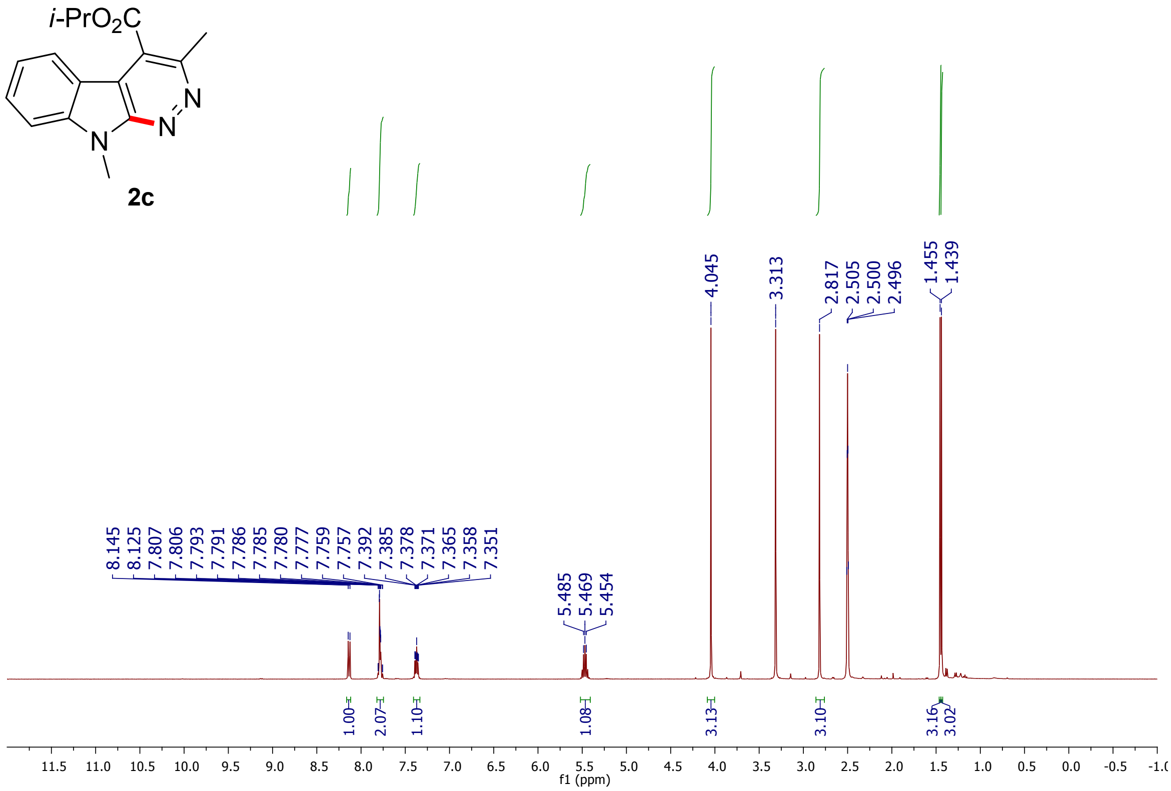

^{13}C{^{1}H} NMR of 2c (100 MHz, DMSO-d_6)

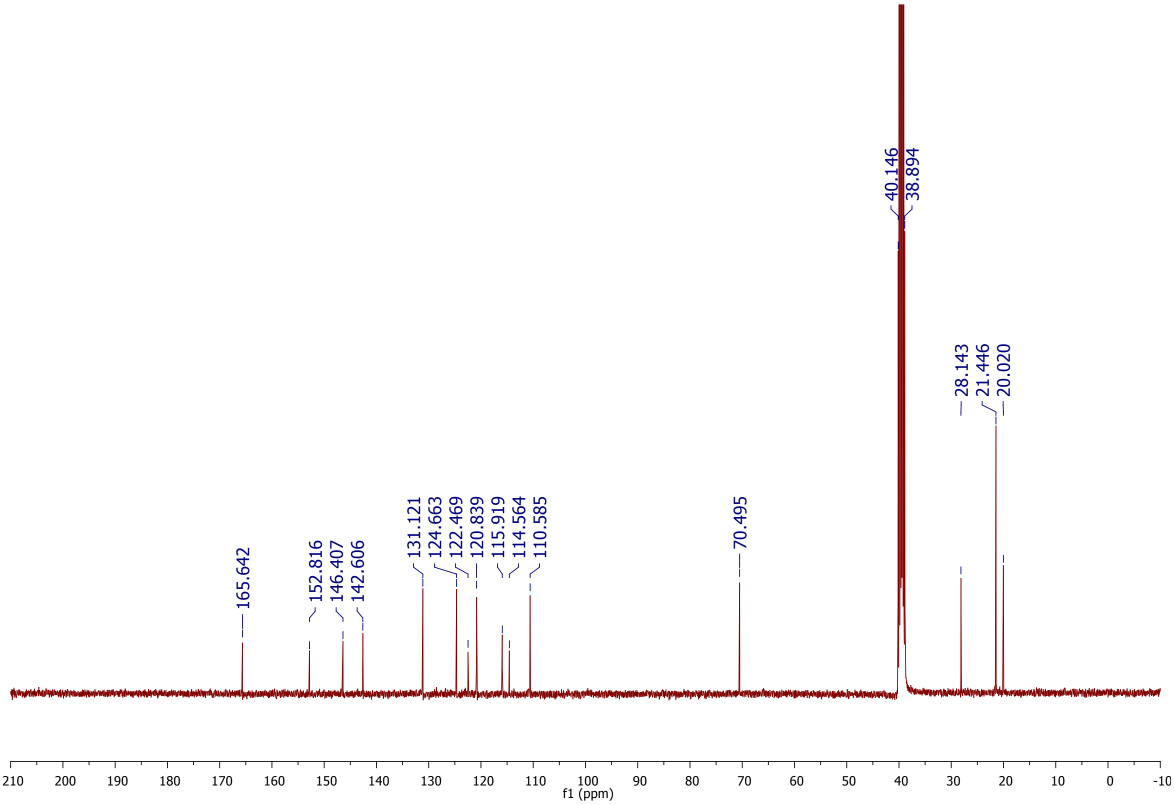

^{1}H NMR of 2d (400 MHz, DMSO-d_6)

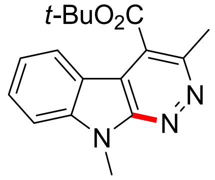

2d

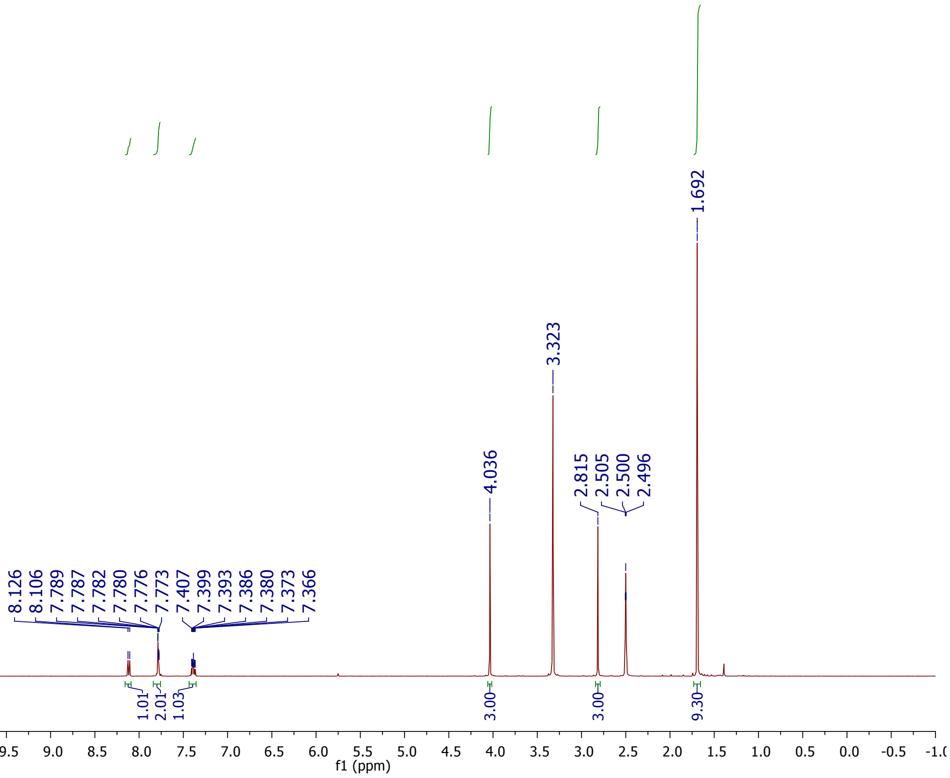

^{13}C{^{1}H} NMR of 2d (100 MHz, DMSO-d_6)

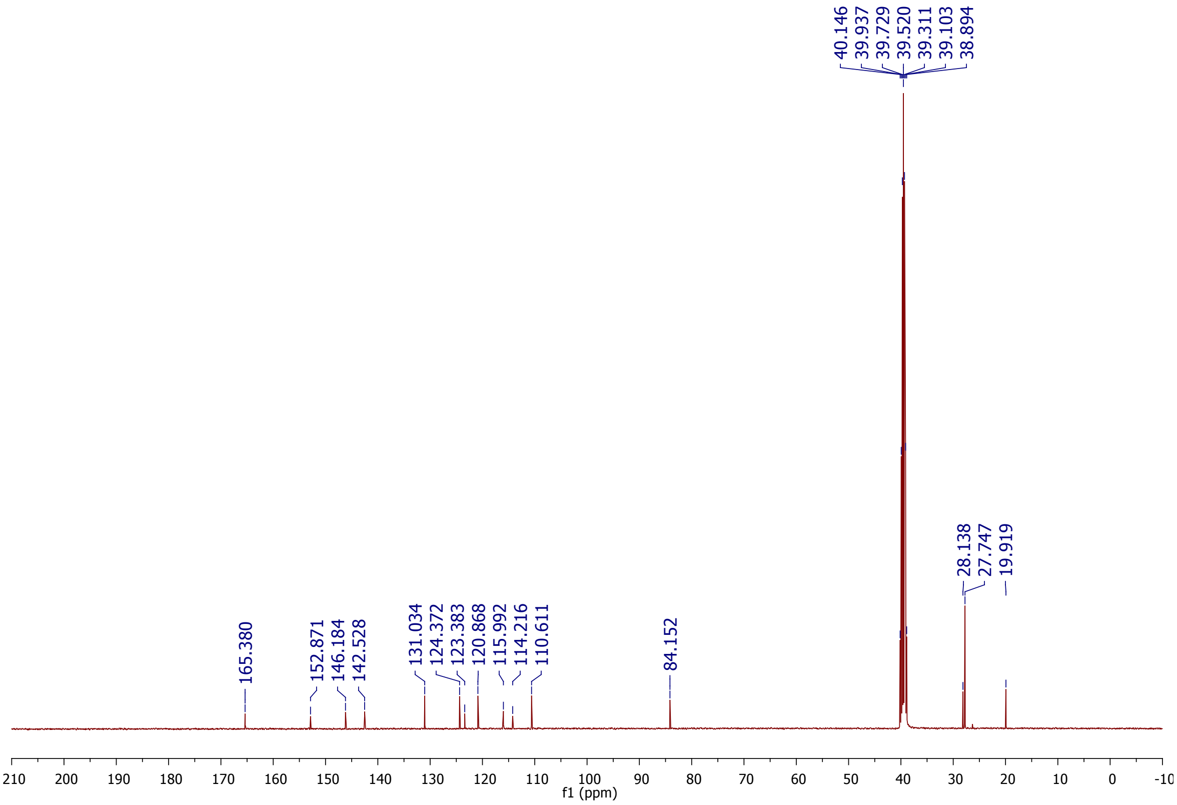

^{1}H NMR of 2e (400 MHz, DMSO-d_6)

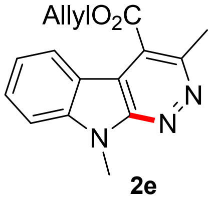

2e

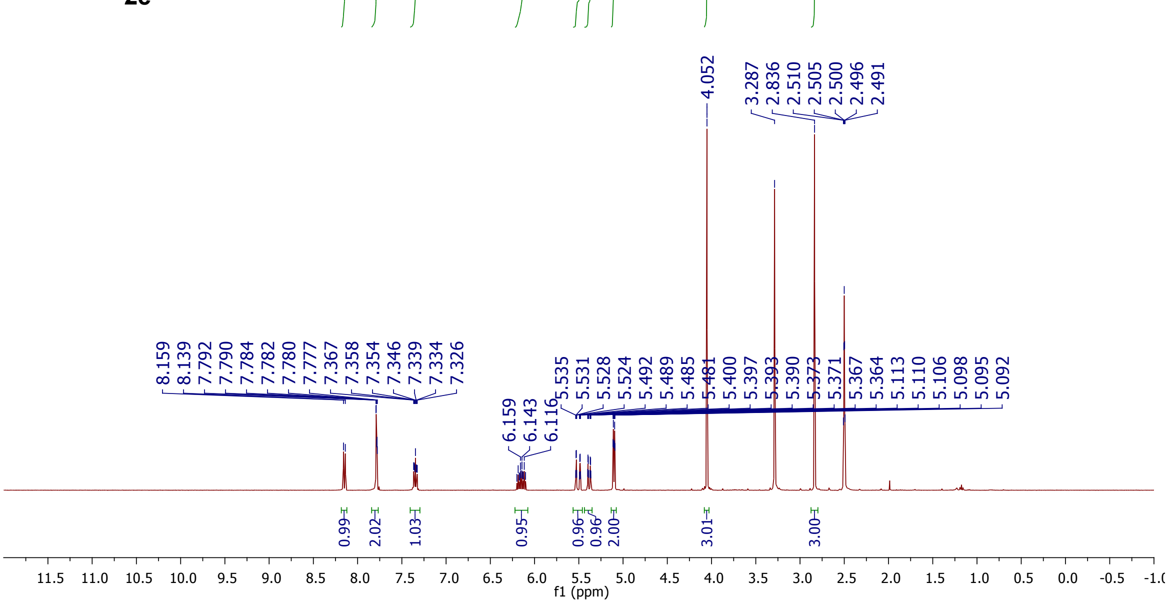

$^{13}C\{^{1}H\}$ NMR of 2e (100 MHz, DMSO-d_6)

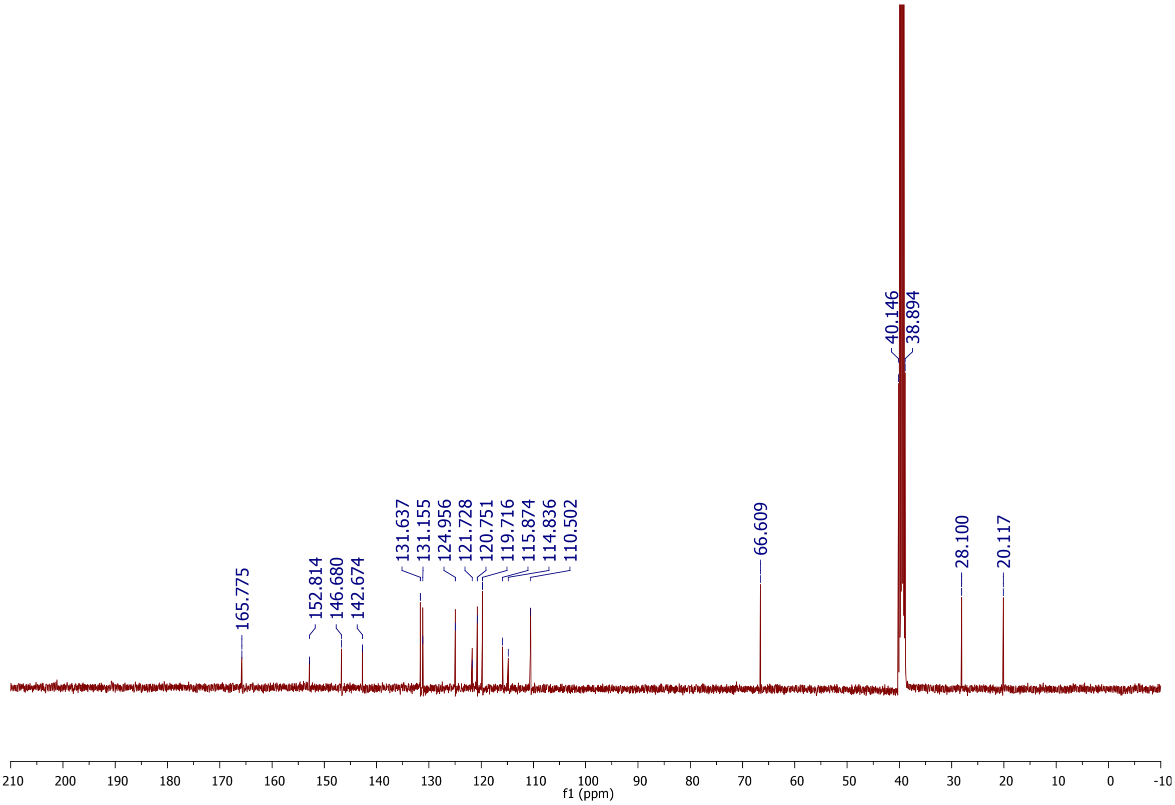

^{1}H NMR of 2f (400 MHz, DMSO-d_6)

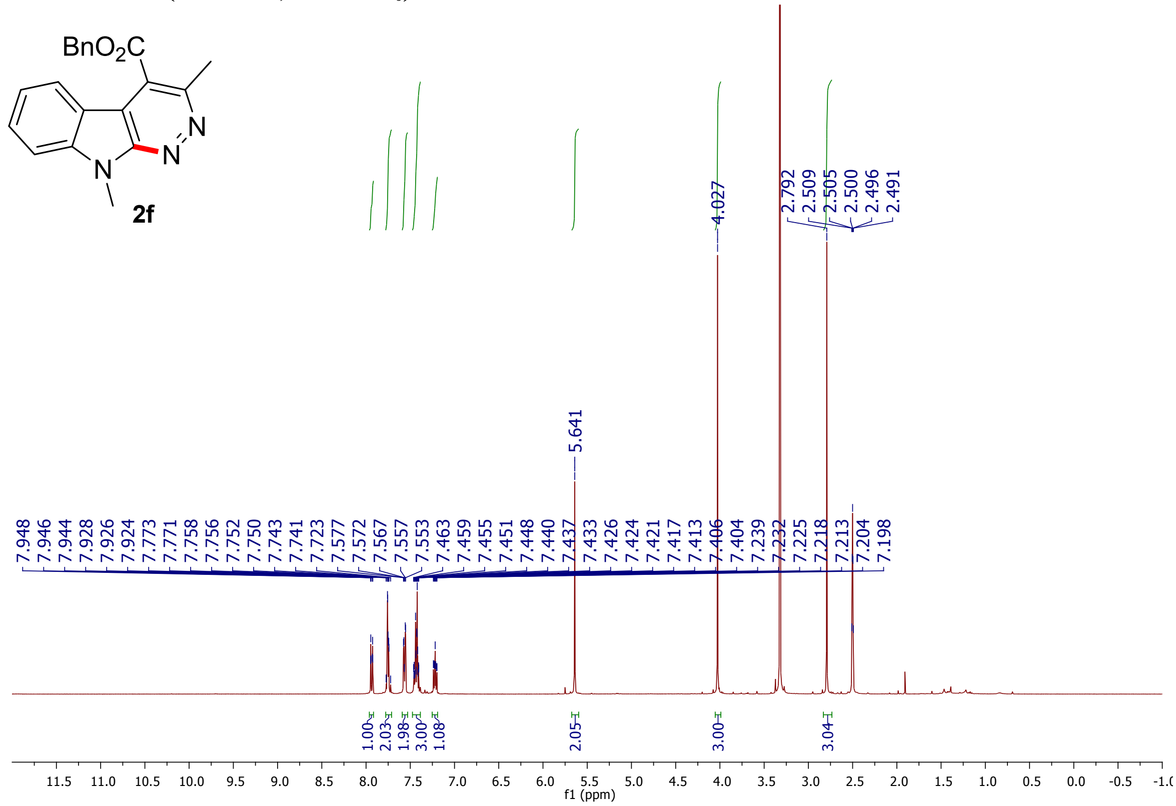

^{13}C{^{1}H} NMR of 2f (100 MHz, DMSO-d_6)

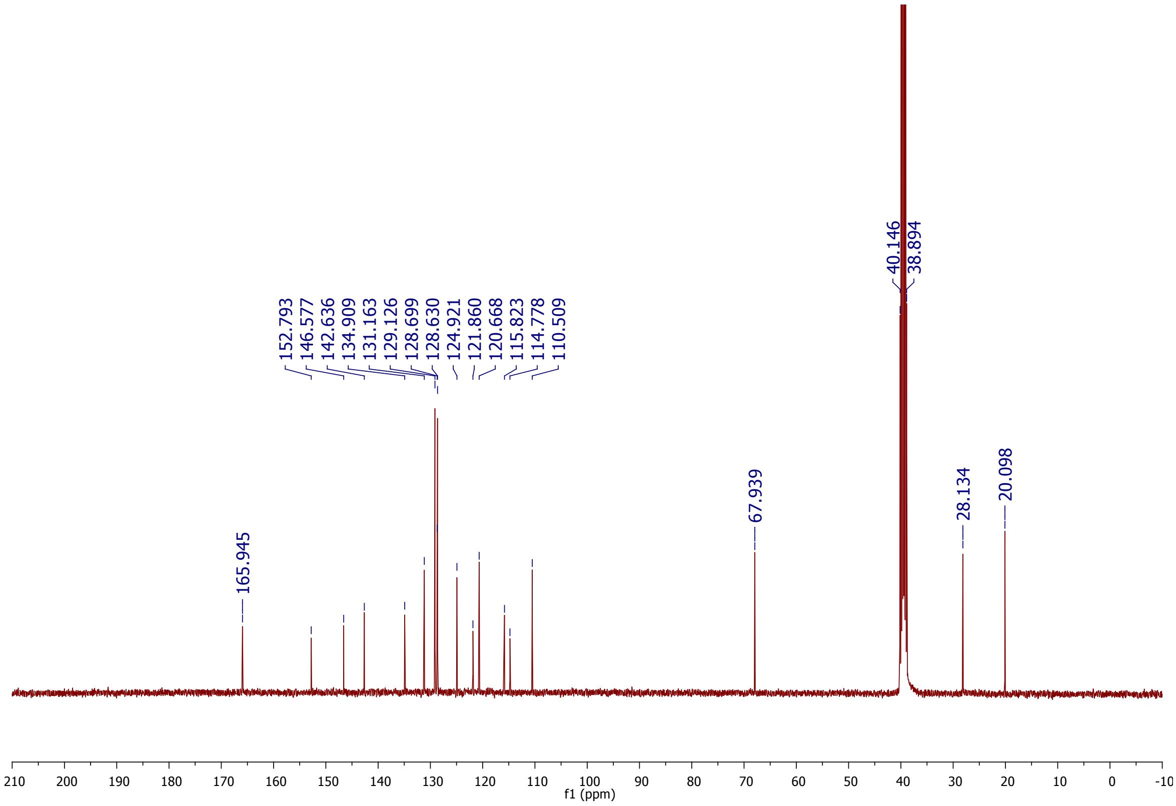

^{1}H NMR of 2g (400 MHz, DMSO-d_6)

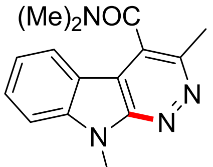

2g

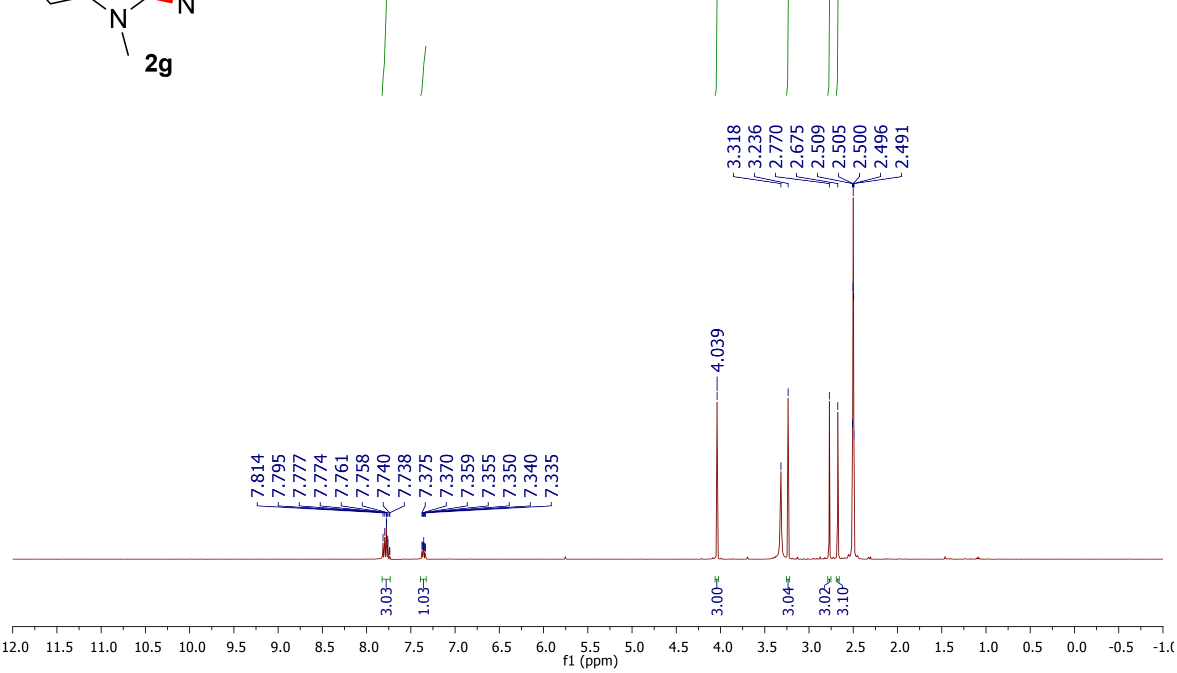

^{13}C{^{1}H} NMR of 2g (100 MHz, DMSO-d_6)

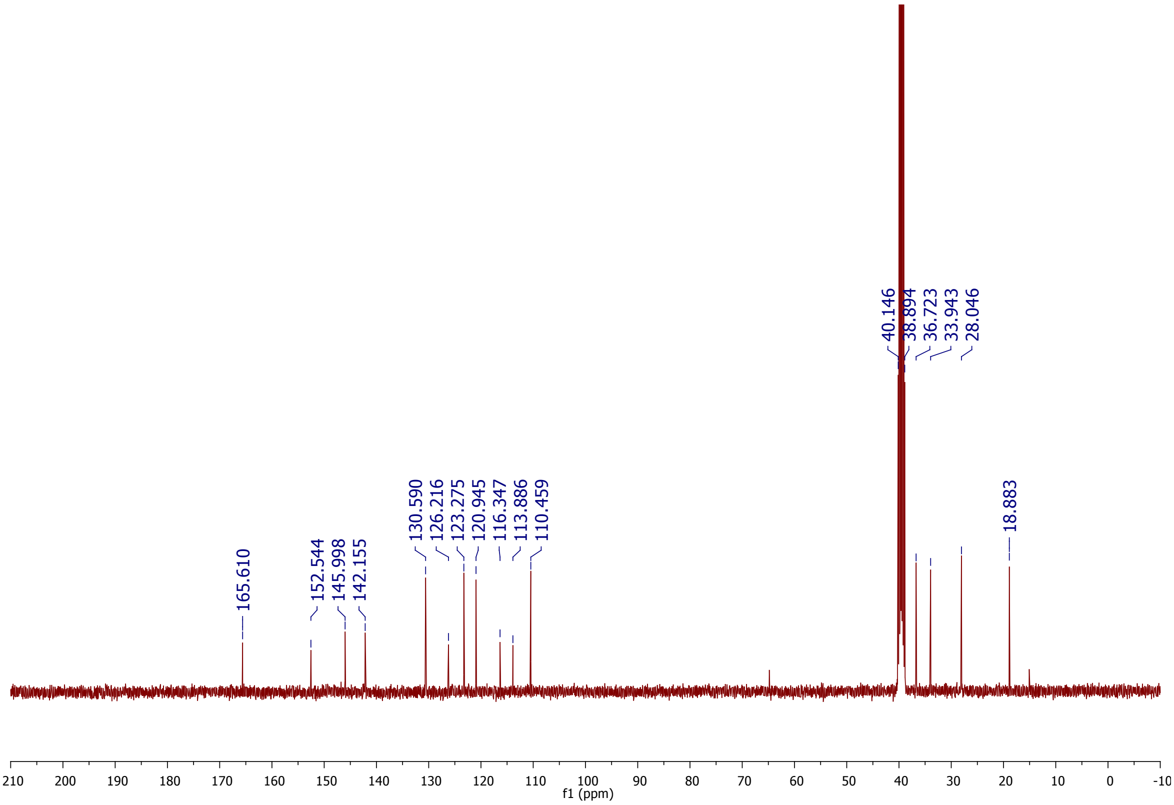

^{1}H NMR of 2h (400 MHz, DMSO-d_6)

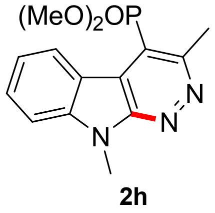

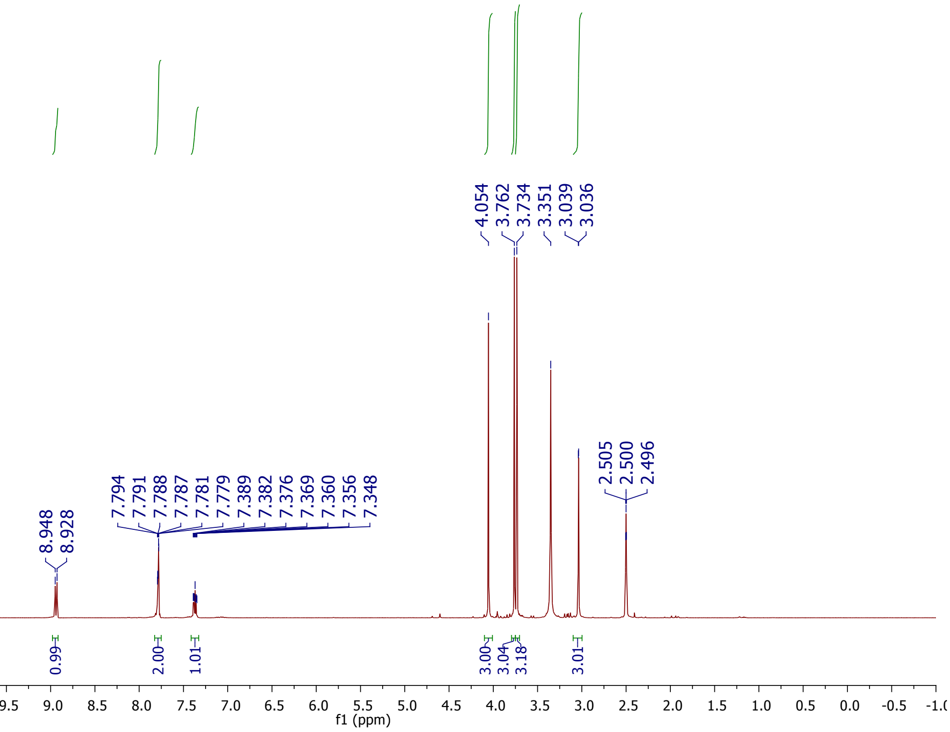

^{13}C{^{1}H} NMR of 2h (100 MHz, DMSO-d_6)

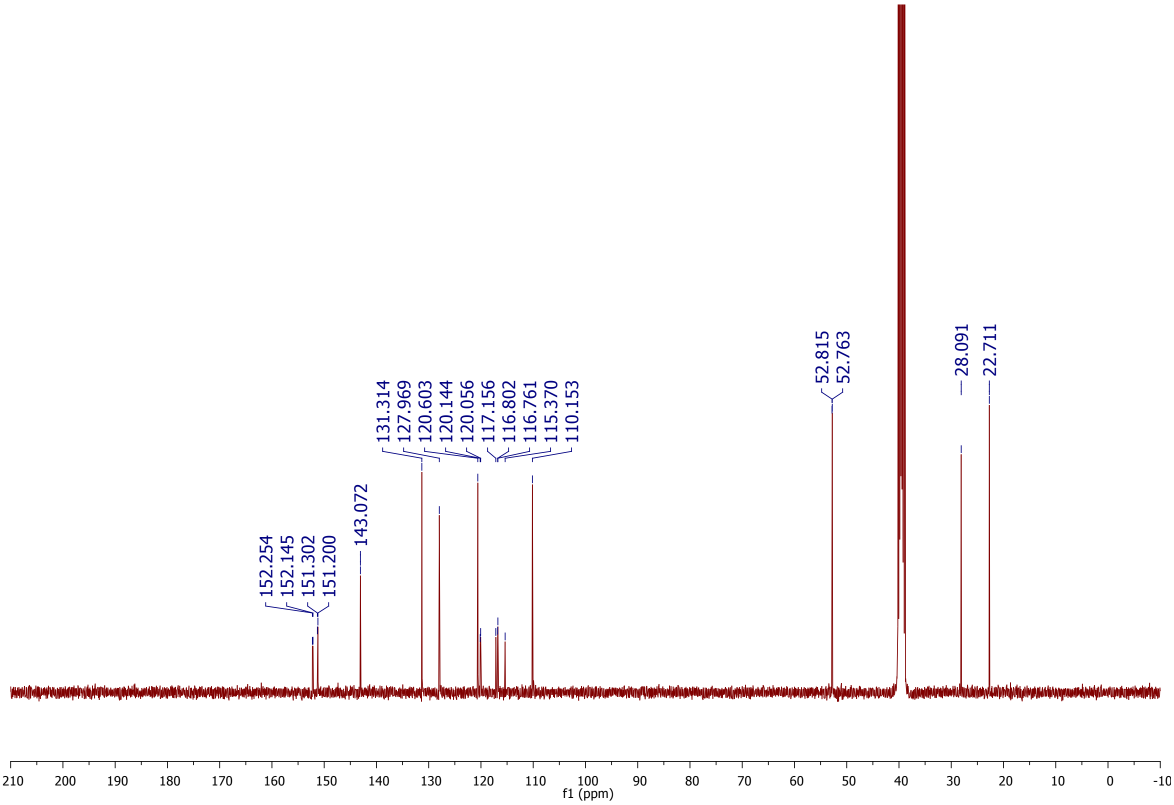

^{1}H NMR of 2i (400 MHz, DMSO-d_6)

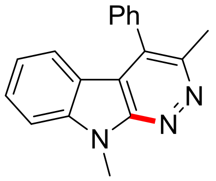

2i

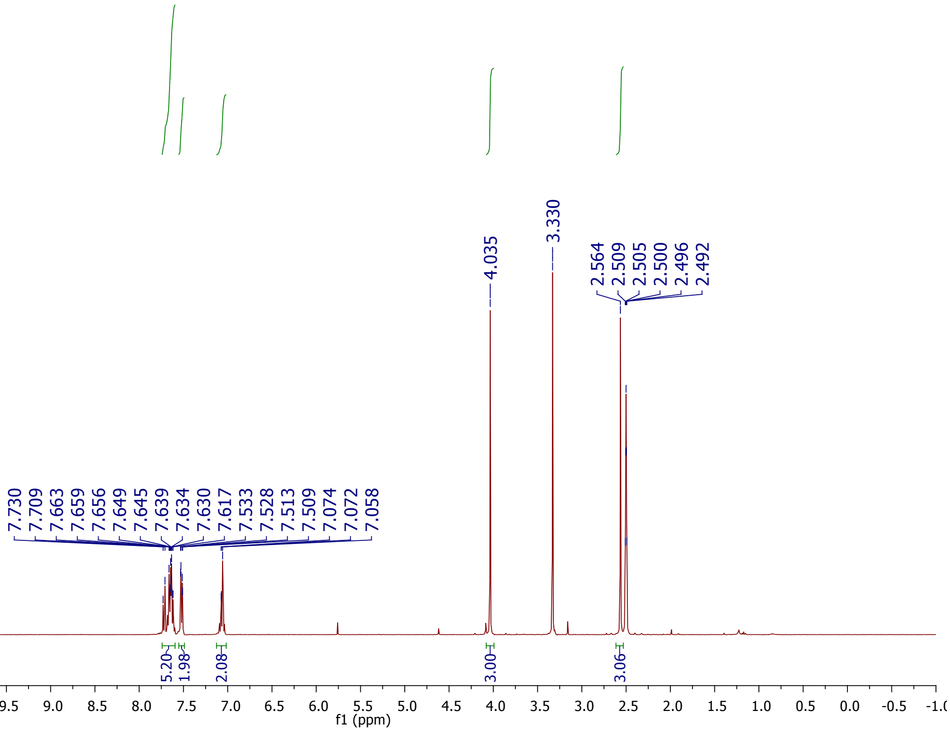

^{13}C{^{1}H} NMR of 2i (100 MHz, DMSO-d_6)

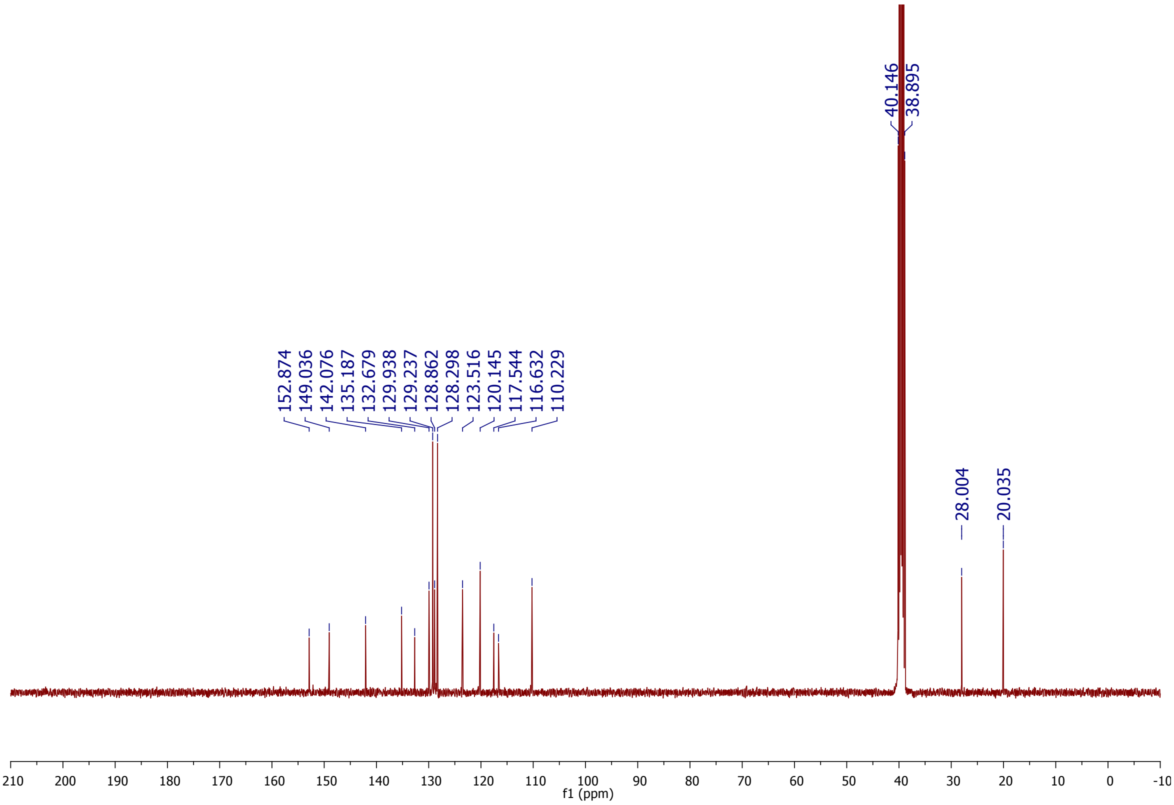

^{1}H NMR of 2j (400 MHz, DMSO-d_6)

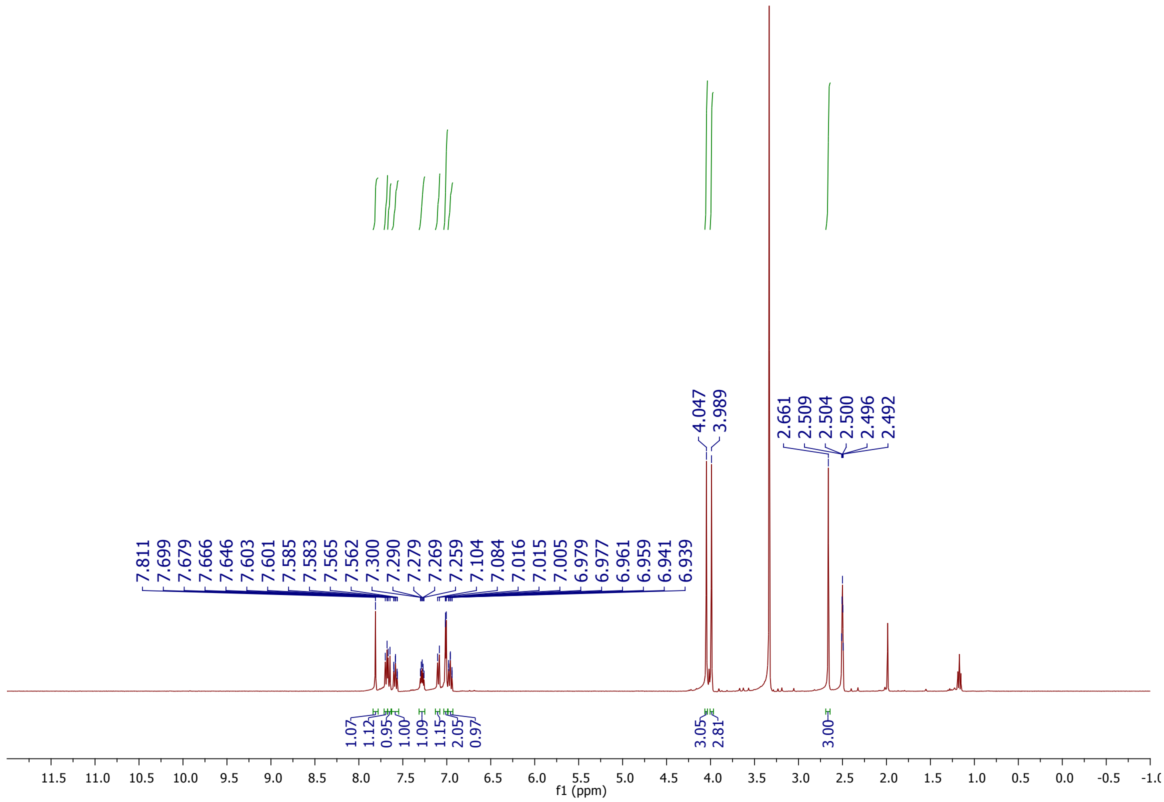

^{13}C{^{1}H} NMR of 2j (100 MHz, DMSO-d_6)

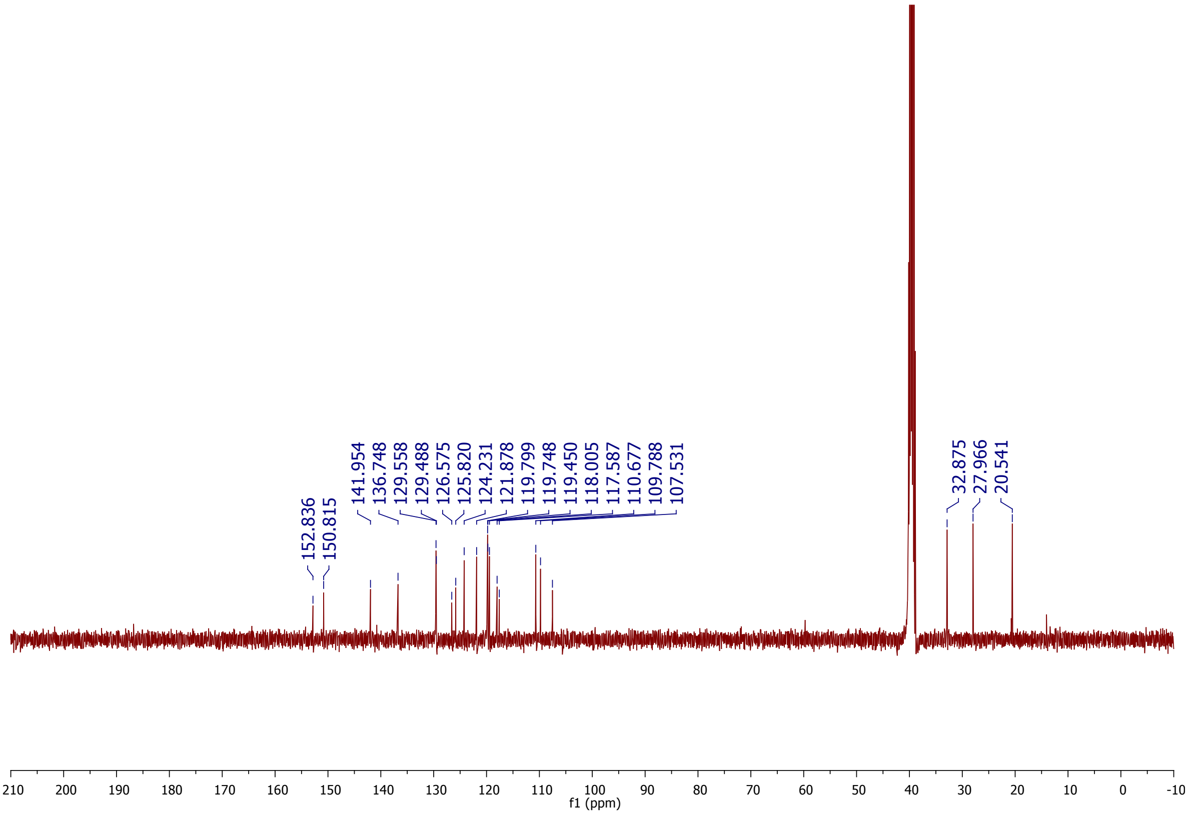

^{1}H NMR of 2k (400 MHz, DMSO-d_6)

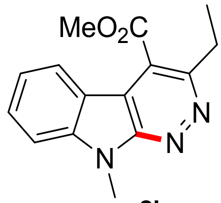

2k

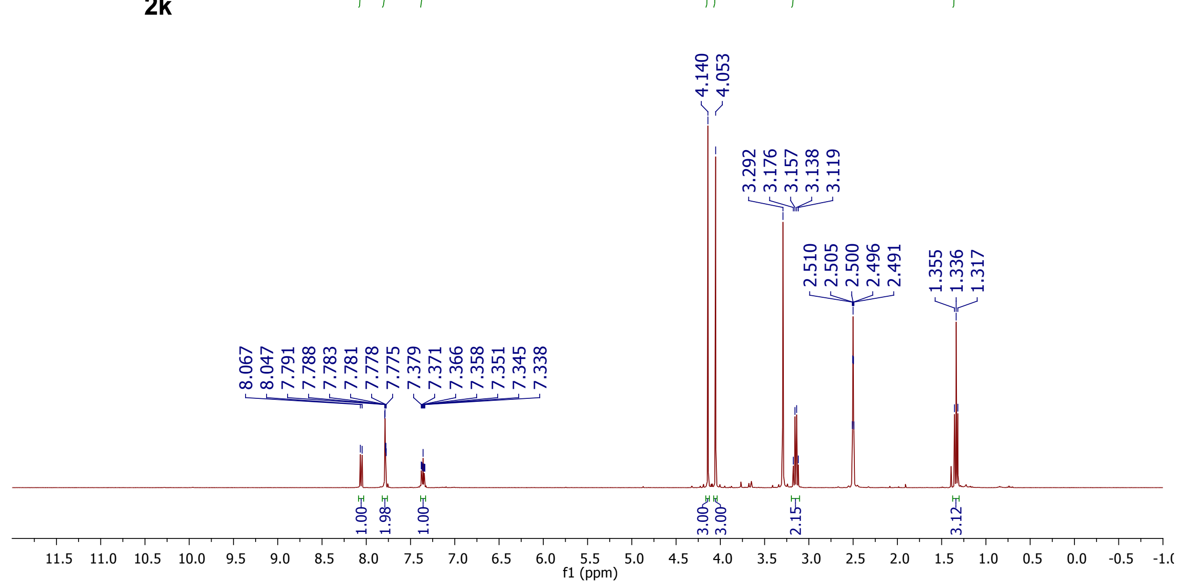

^{13}C{^{1}H} NMR of 2k (100 MHz, DMSO-d_6)

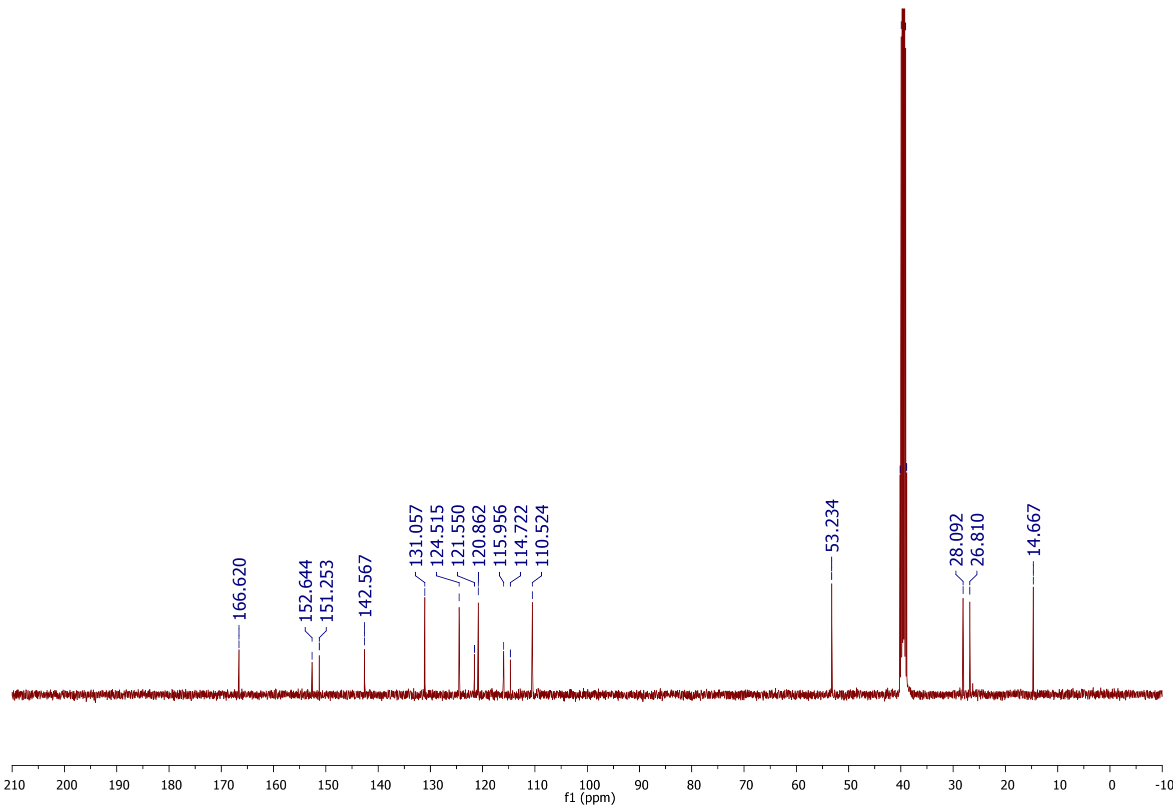

^{1}H NMR of 2l (400 MHz, DMSO-d_6)

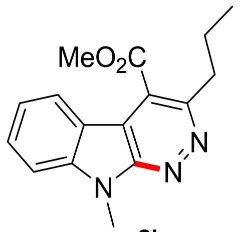

2l

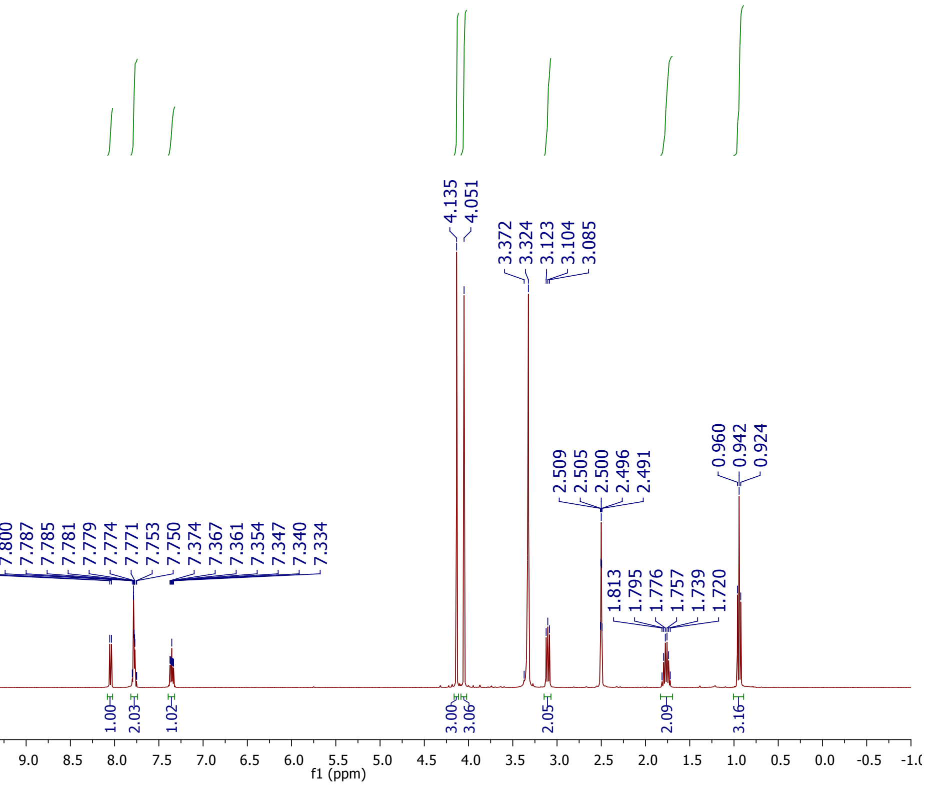

^{13}C{^{1}H} NMR of 2l (100 MHz, DMSO-d_6)

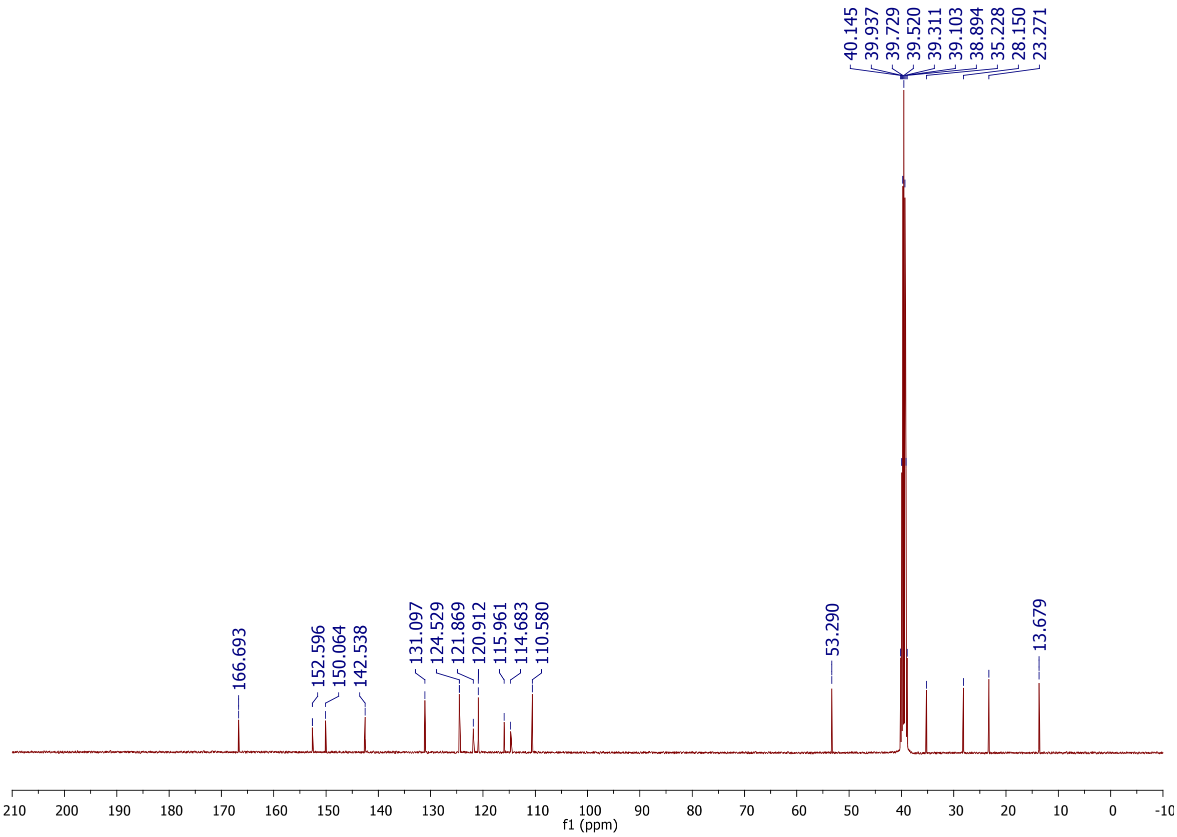

^{1}H NMR of 2m (400 MHz, DMSO-d_6)

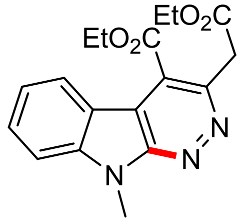

2m

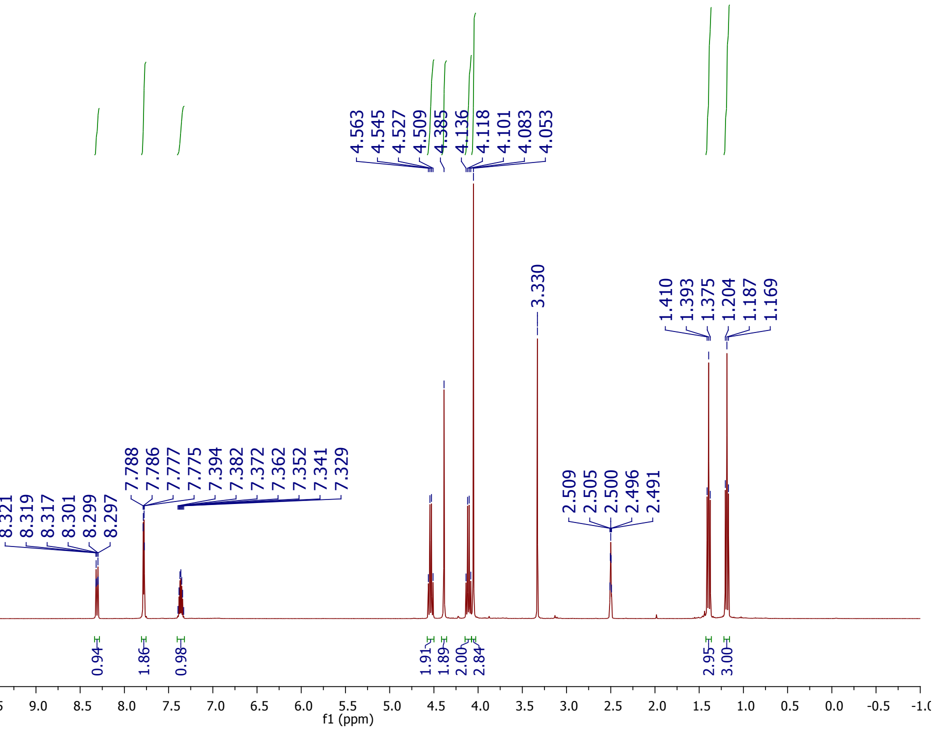

$^{13}C\{^{1}H\}$ NMR of 2m (100 MHz, DMSO-d_6)

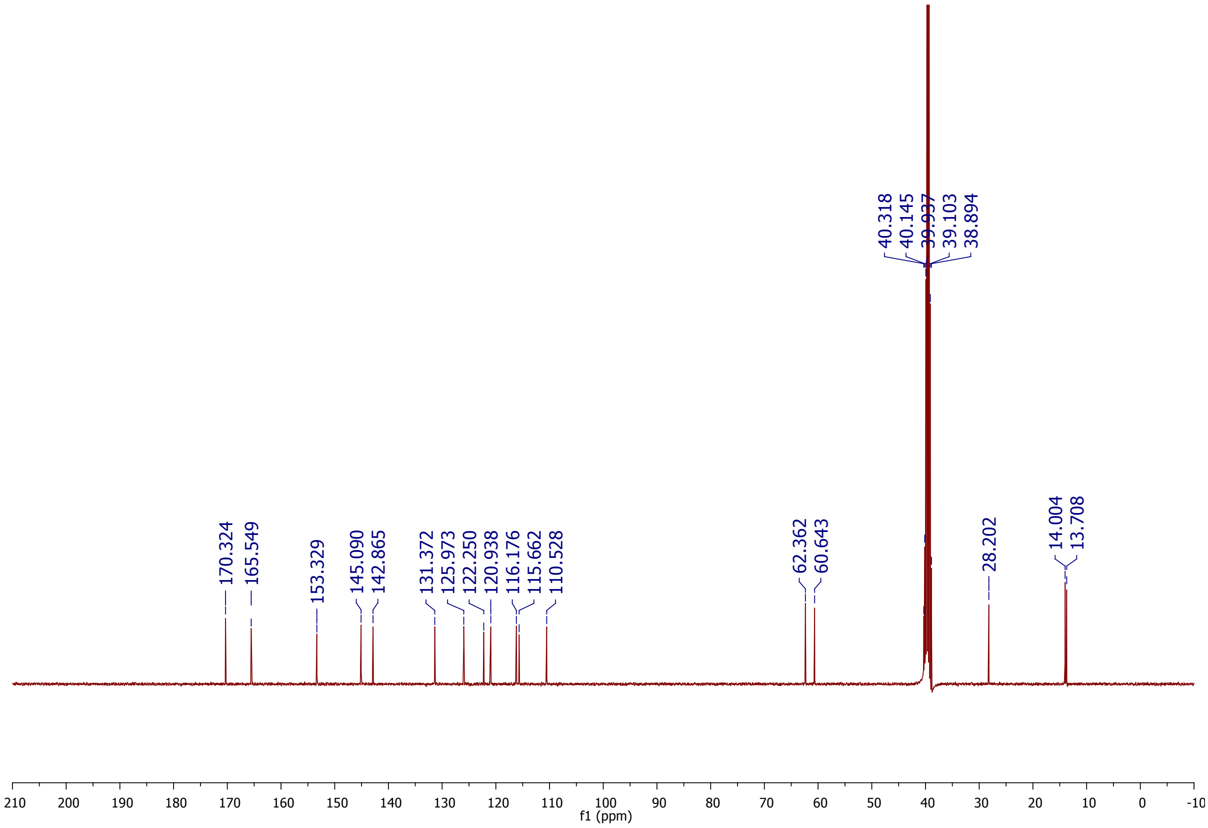

^{1}H NMR of 2n (400 MHz, DMSO-d_6)

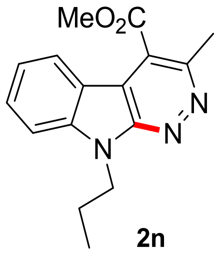

2n

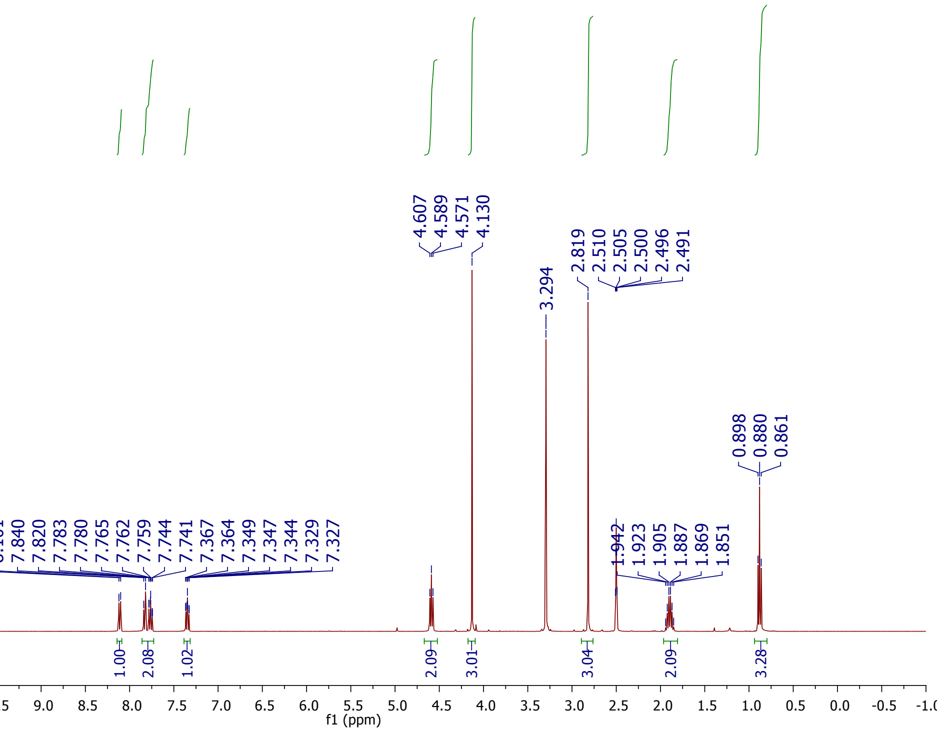

^{13}C{^{1}H} NMR of 2n (100 MHz, DMSO-d_6)

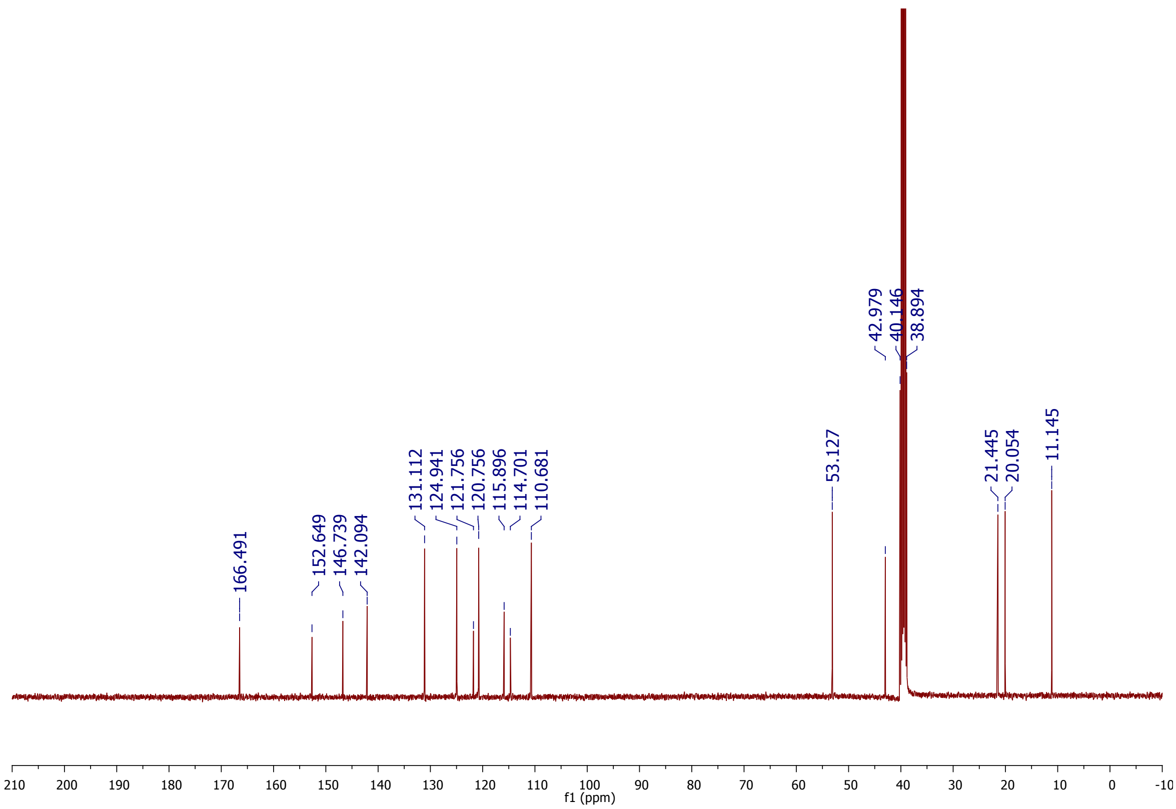

^{1}H NMR of 2o (400 MHz, DMSO-d_6)

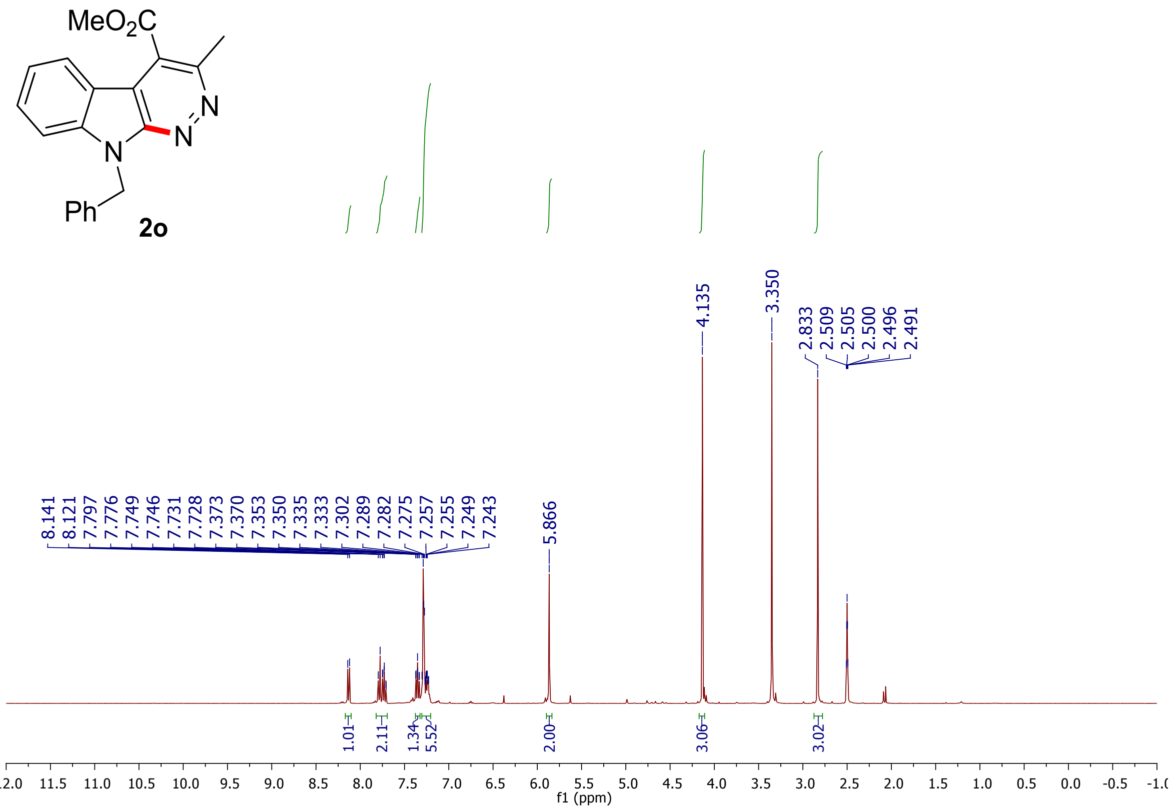

^{13}C{^{1}H} NMR of 2o (100 MHz, DMSO-d_6)

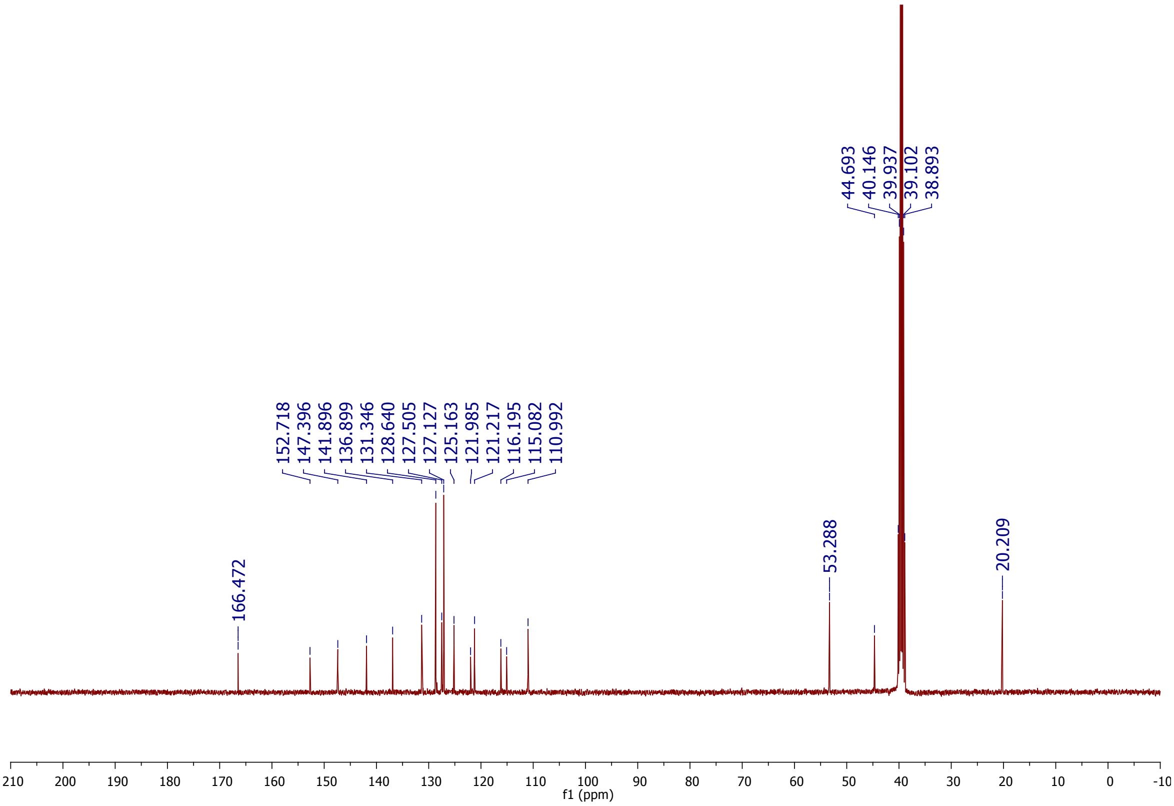

^{1}H NMR of 2p (400 MHz, DMSO-d_6)

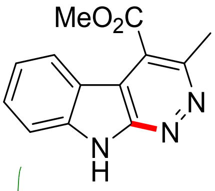

2p

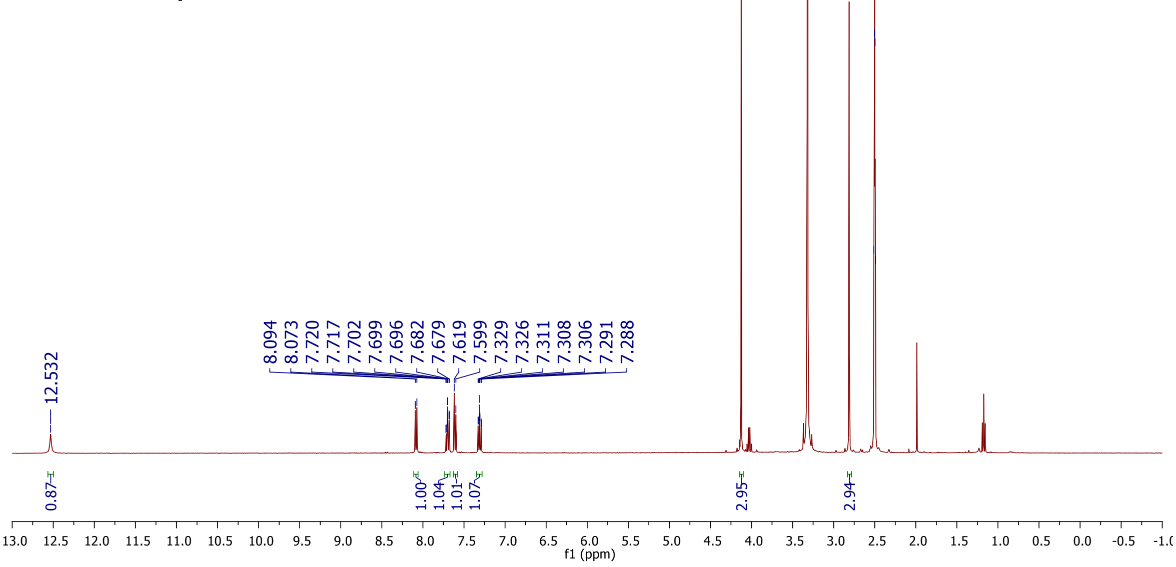

^{13}C{^{1}H} NMR of 2p (100 MHz, DMSO-d_6)

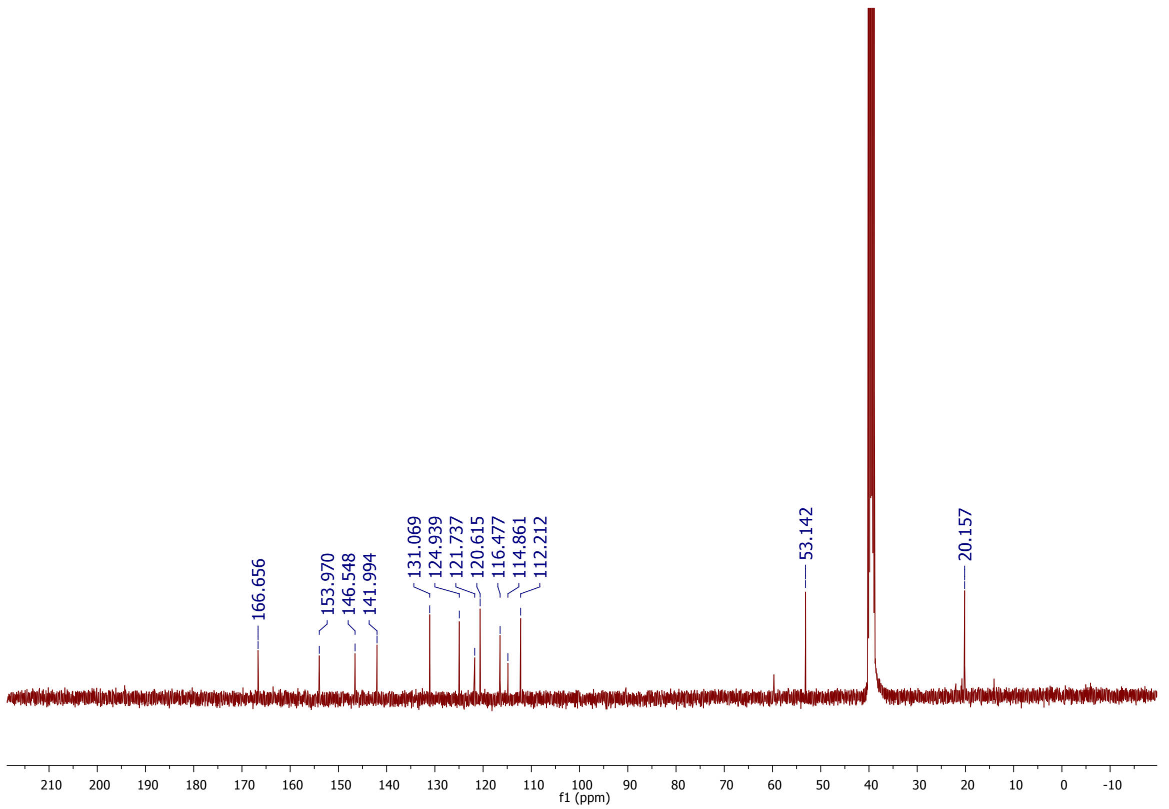

^{1}H NMR of 2q (400 MHz, DMSO-d_6)

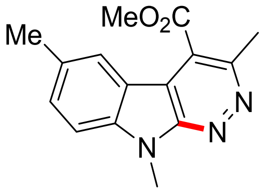

2q

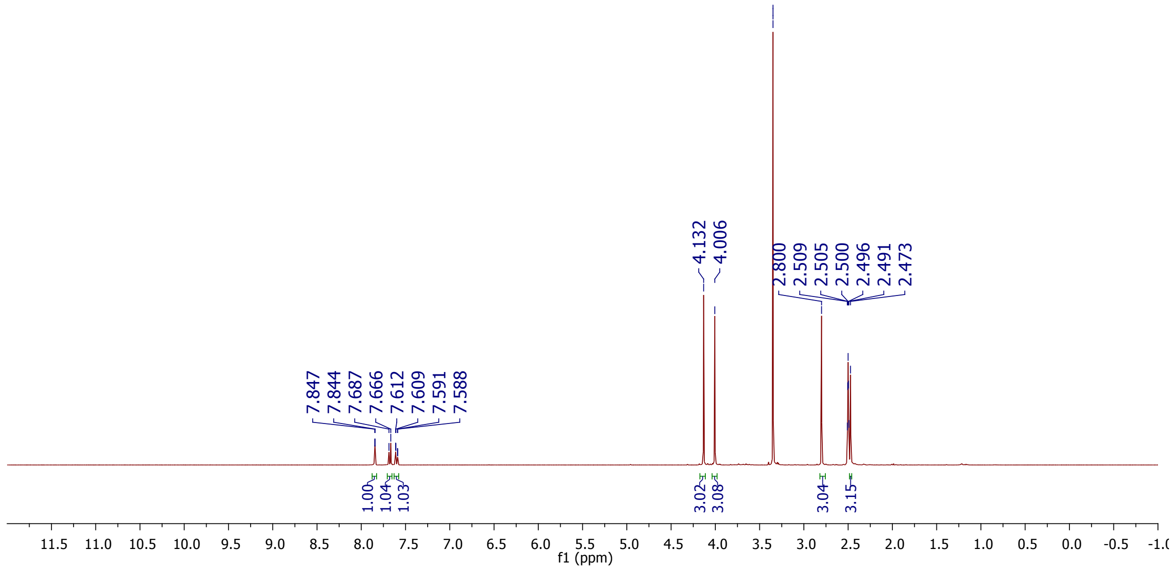

^{13}C{^{1}H} NMR of 2q (100 MHz, DMSO-d_6)

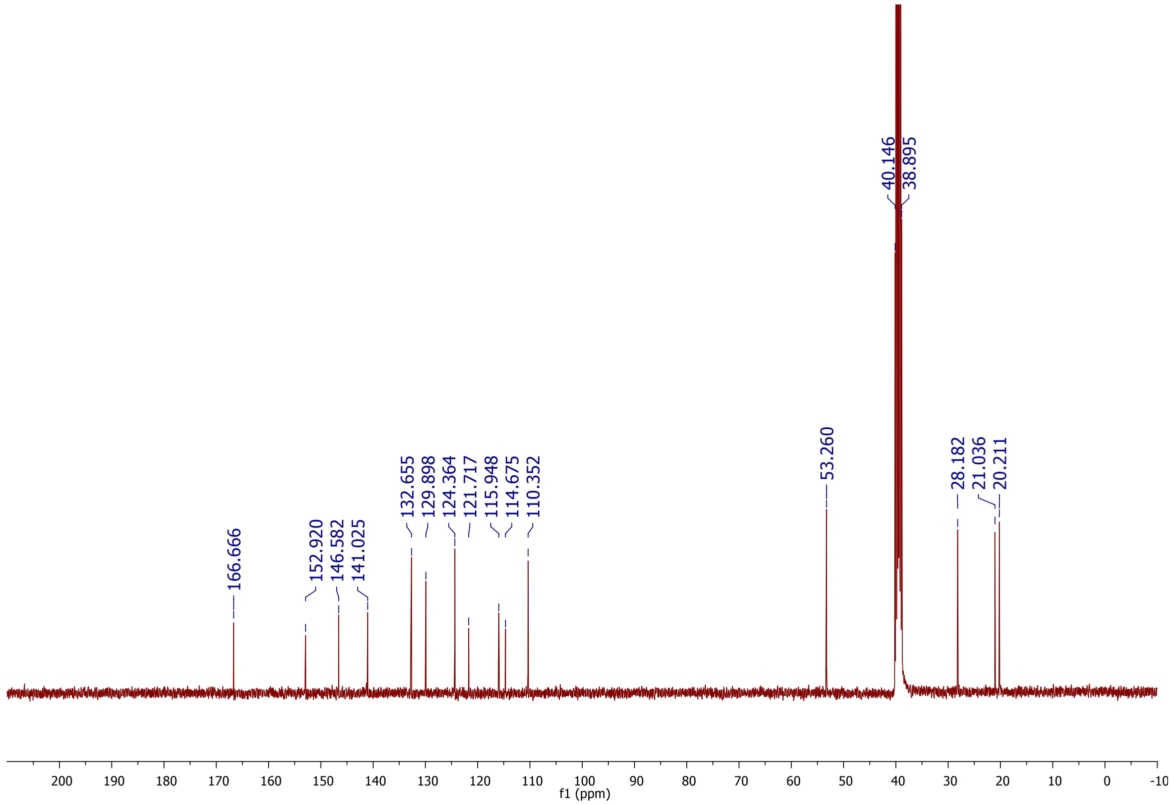

^{1}H NMR of 2r (400 MHz, DMSO-d_6)

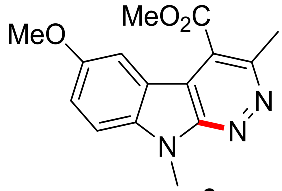

2r

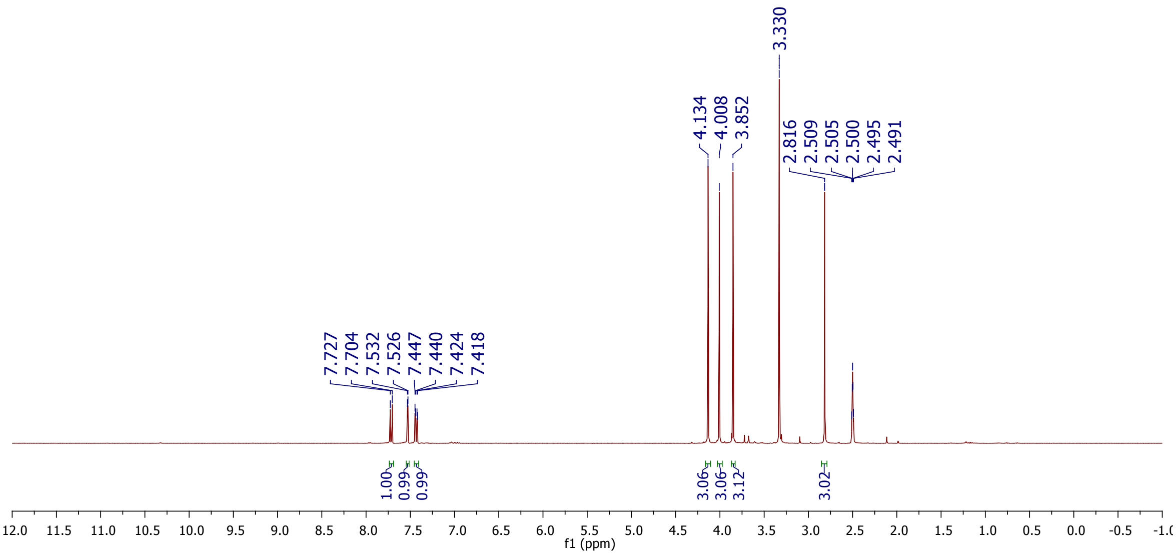

^{13}C{^{1}H} NMR of 2r (100 MHz, DMSO-d_6)

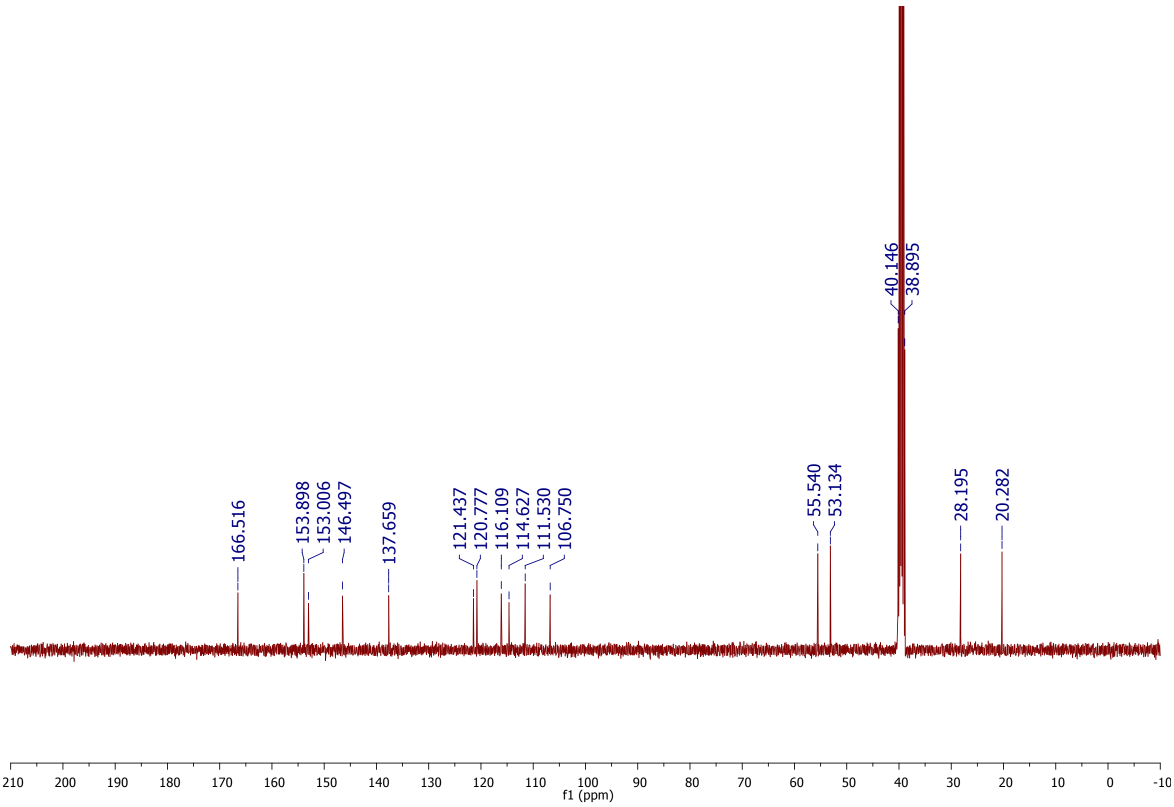

^{1}H NMR of 2s (400 MHz, DMSO-d_6)

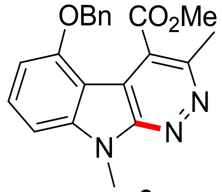

2s

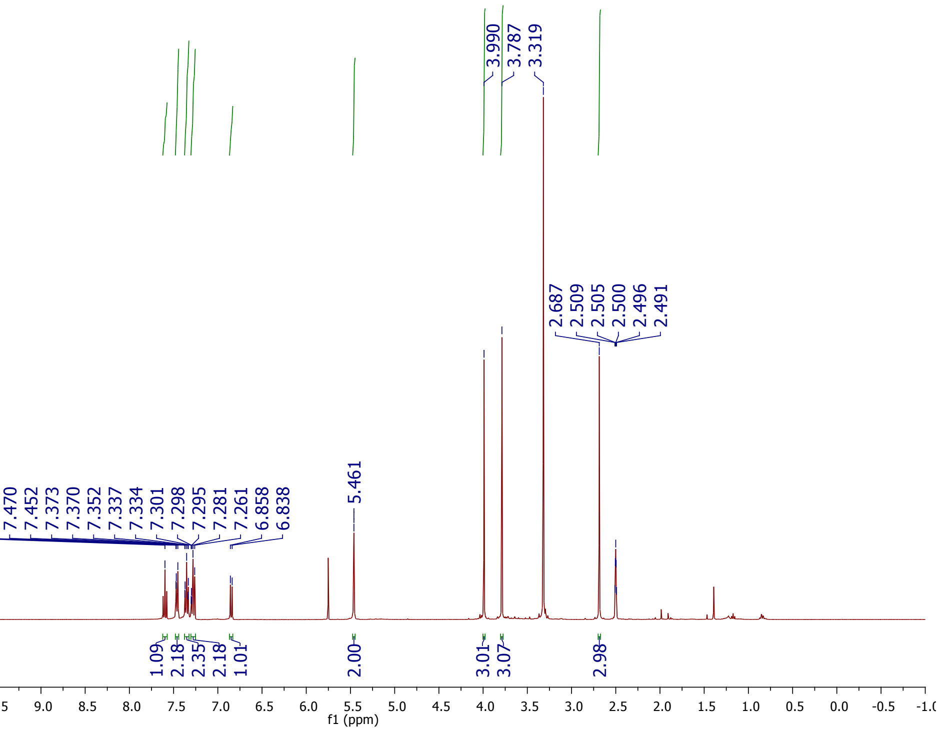

^{13}C{^{1}H} NMR of 2s (100 MHz, DMSO-d_6)

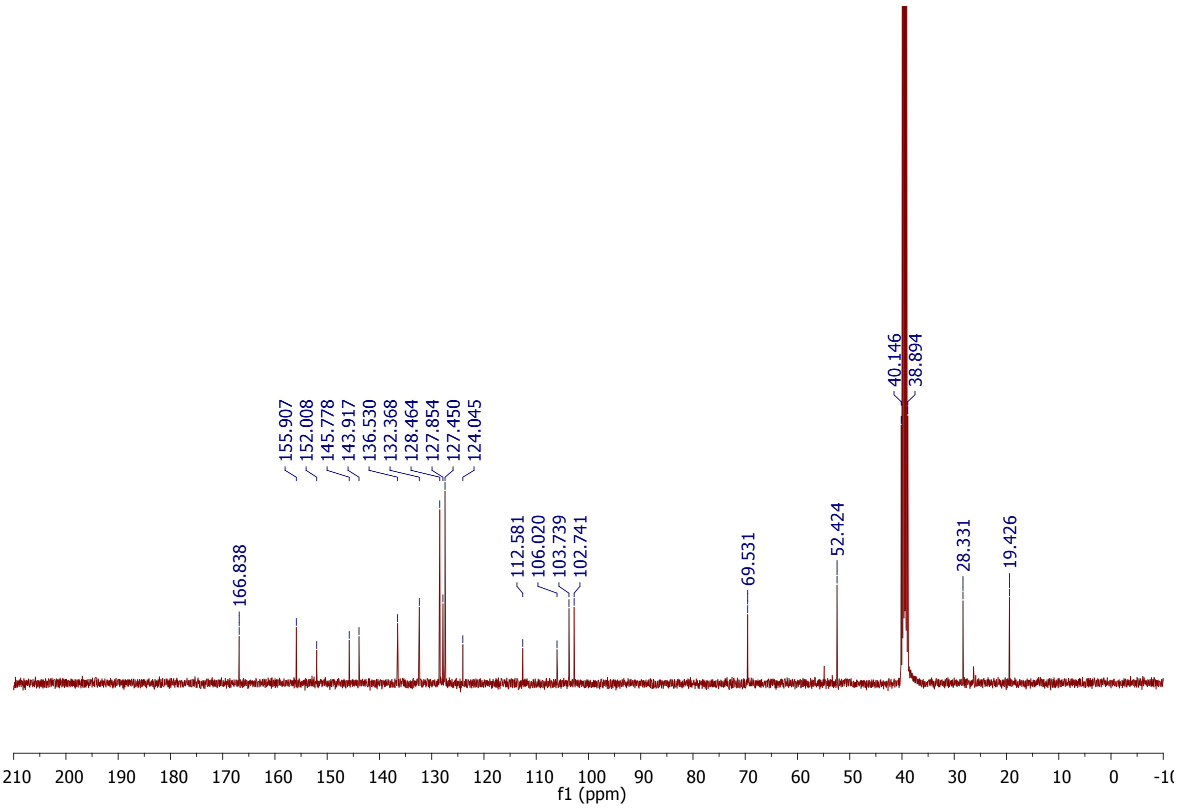

^{1}H NMR of 2t (400 MHz, DMSO-d_6)

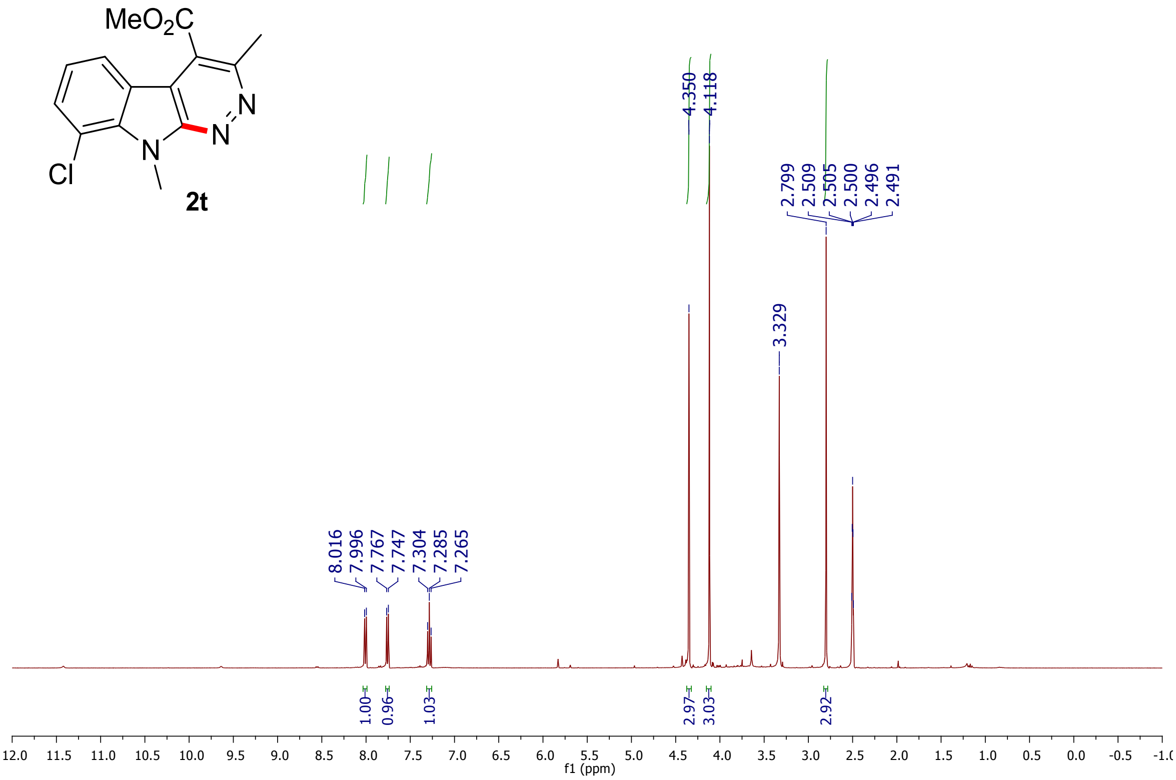

$^{13}C\{^{1}H\}$ NMR of 2t (100 MHz, DMSO-d_6)

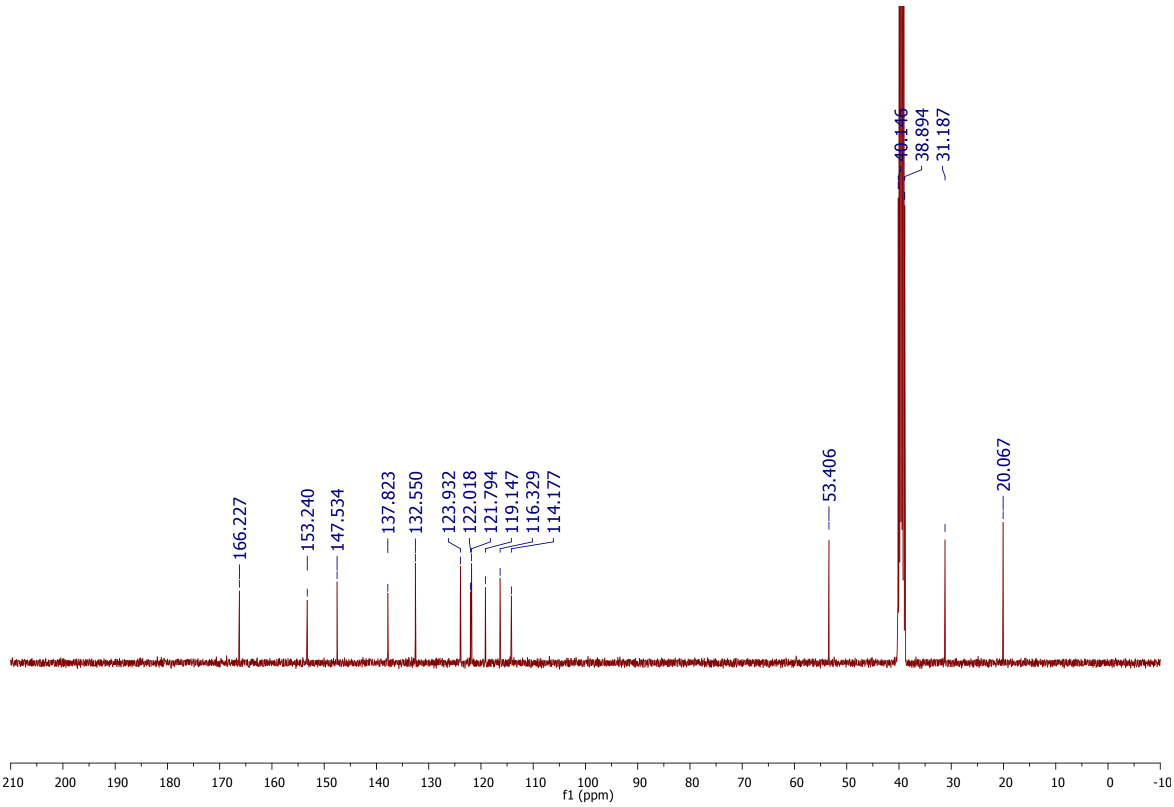

^{1}H NMR of 2u (400 MHz, DMSO-d_6)

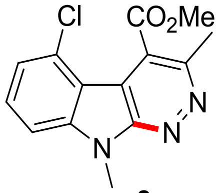

2u

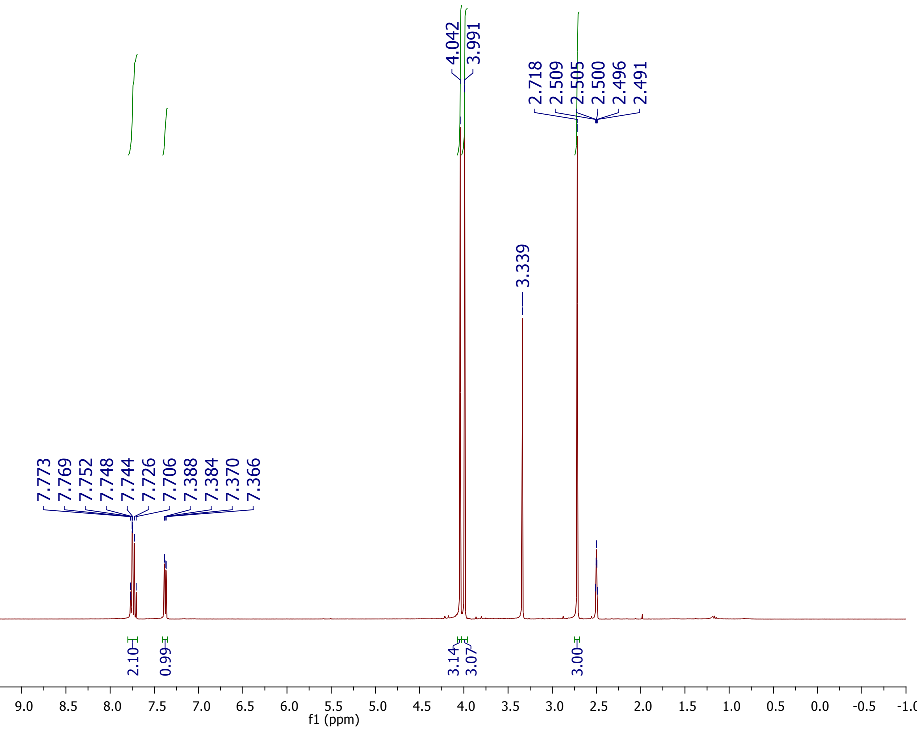

^{13}C{^{1}H} NMR of 2u (100 MHz, DMSO-d_6)

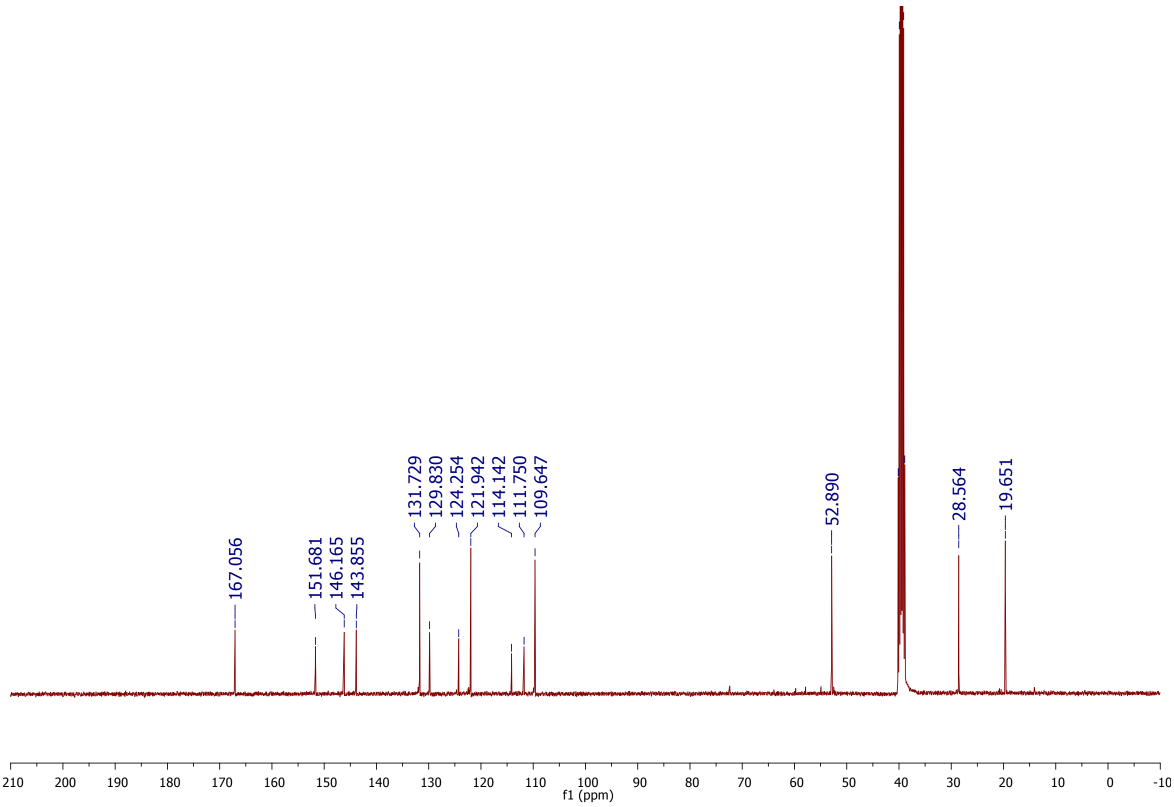

^{1}H NMR of 2v (400 MHz, DMSO-d_6)

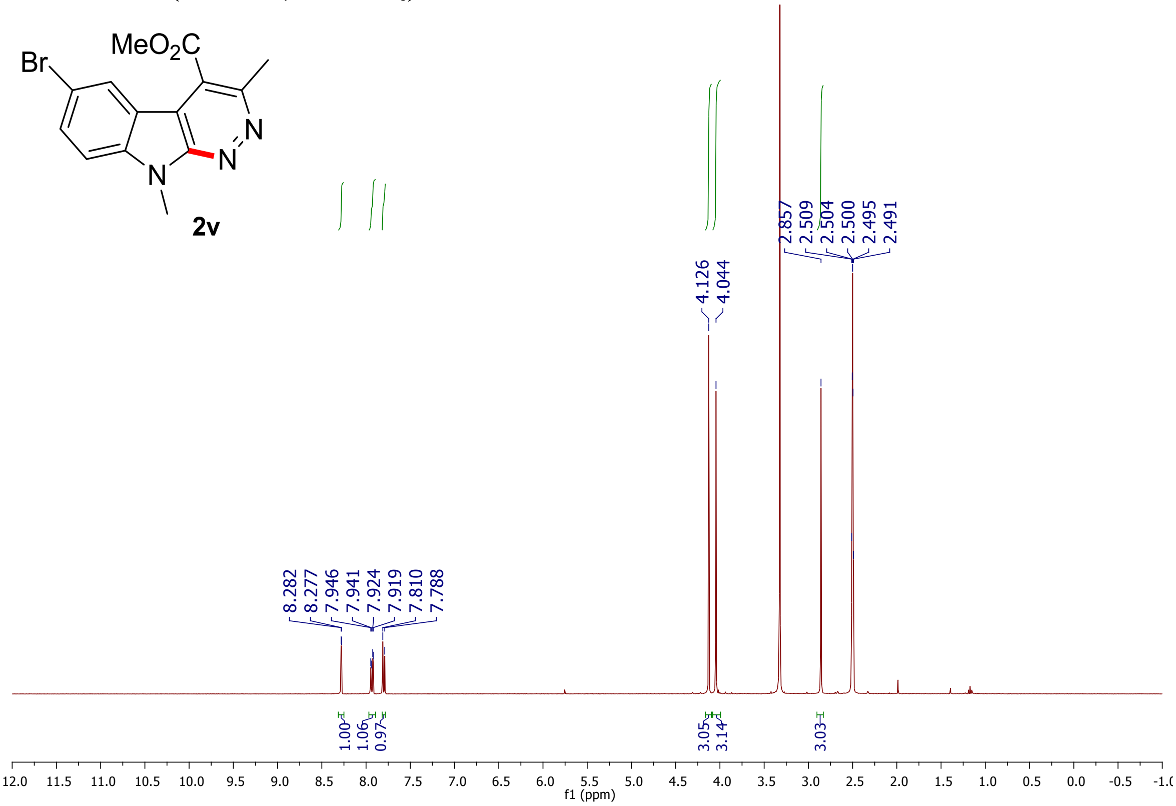

^{13}C{^{1}H} NMR of 2v (100 MHz, DMSO-d_6)

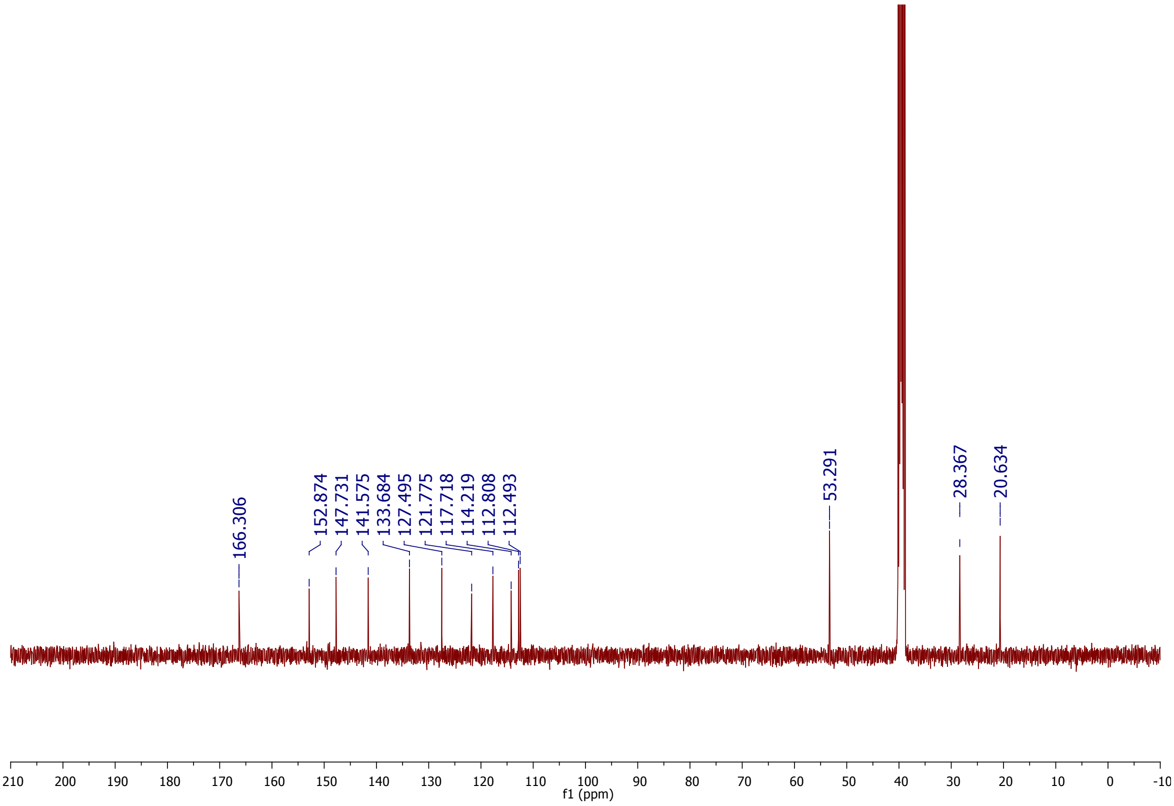

^{1}H NMR of 2w (400 MHz, DMSO-d_6)

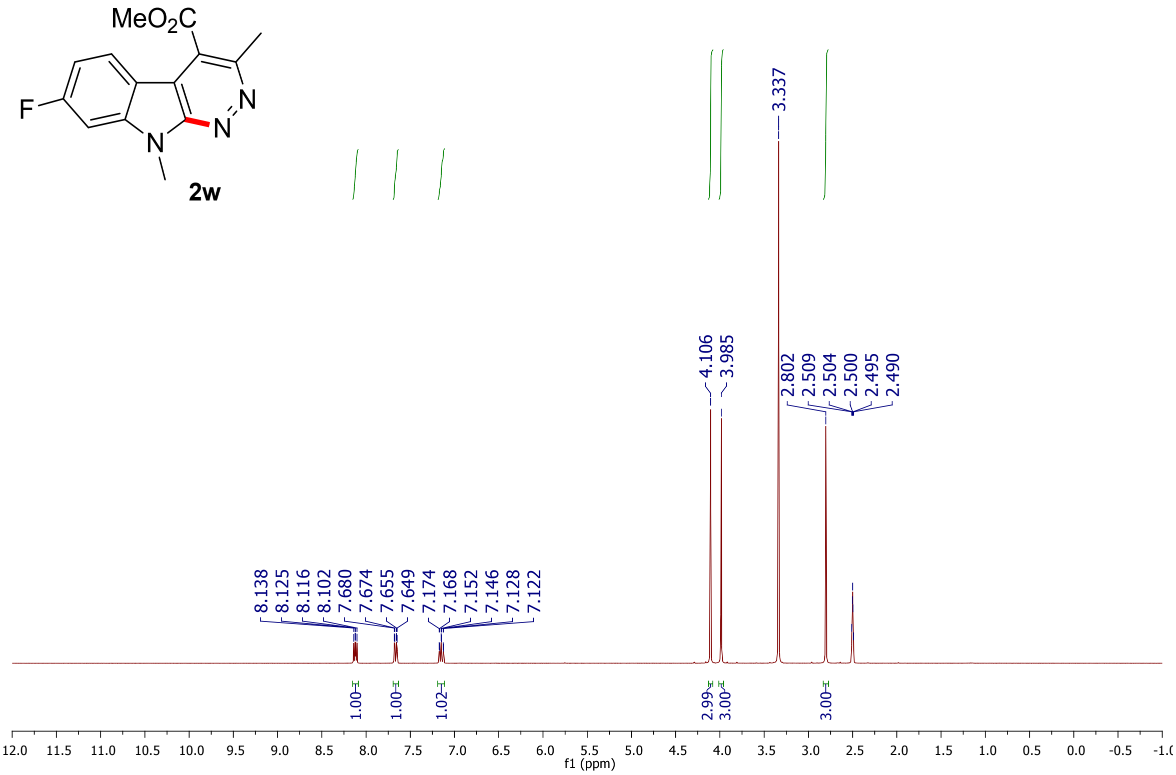

^{13}C{^{1}H} NMR of 2w (100 MHz, $CDCl_3$)

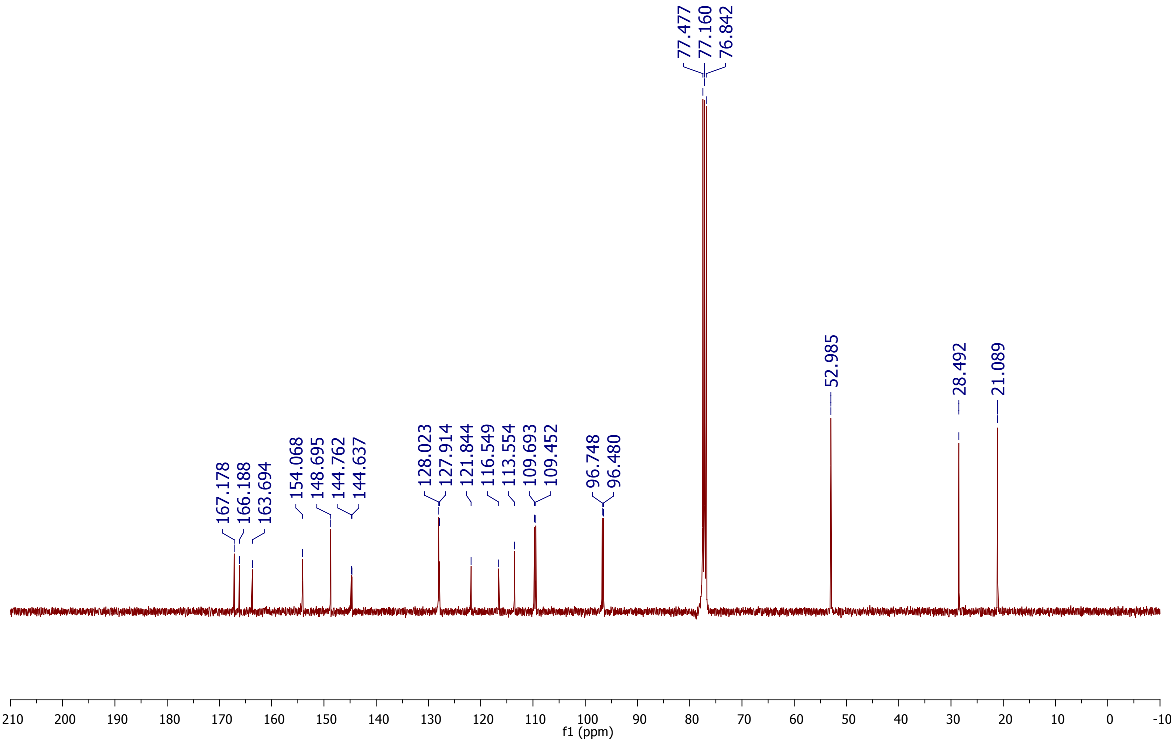

^{1}H NMR of 2x (400 MHz, DMSO-d_6)

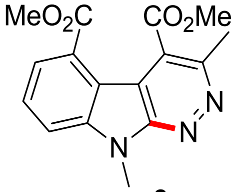

2x

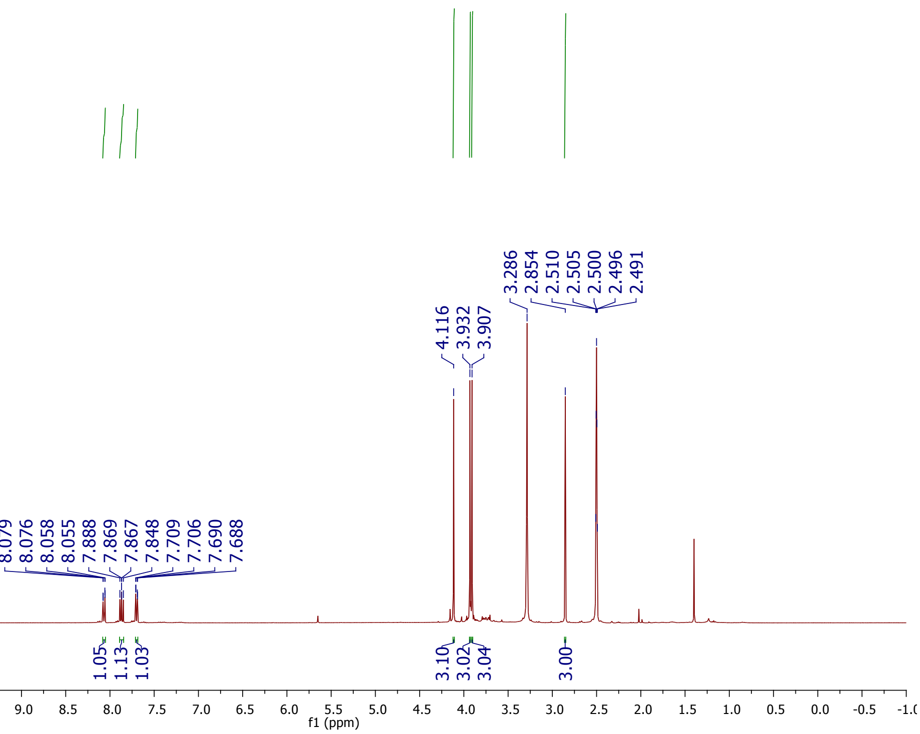

^{13}C{^{1}H} NMR of 2x (100 MHz, DMSO-d_6)

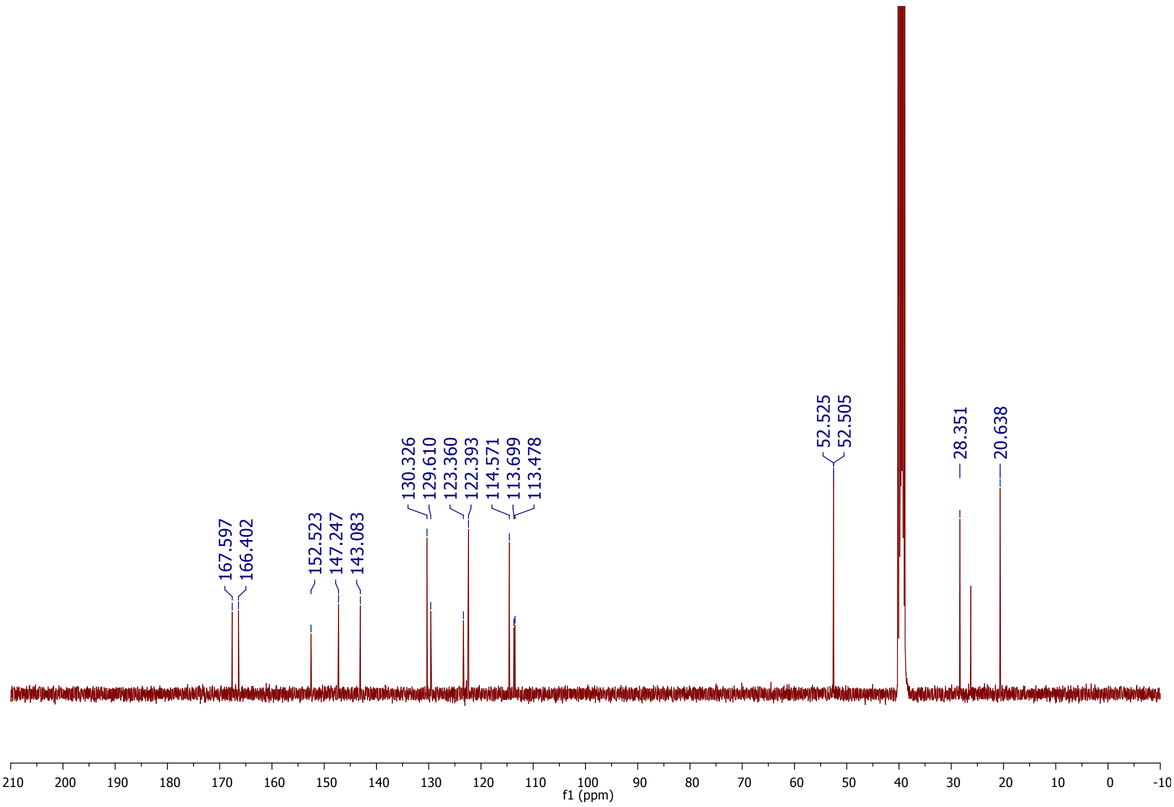

^{1}H NMR of 2y (400 MHz, DMSO-d_6)

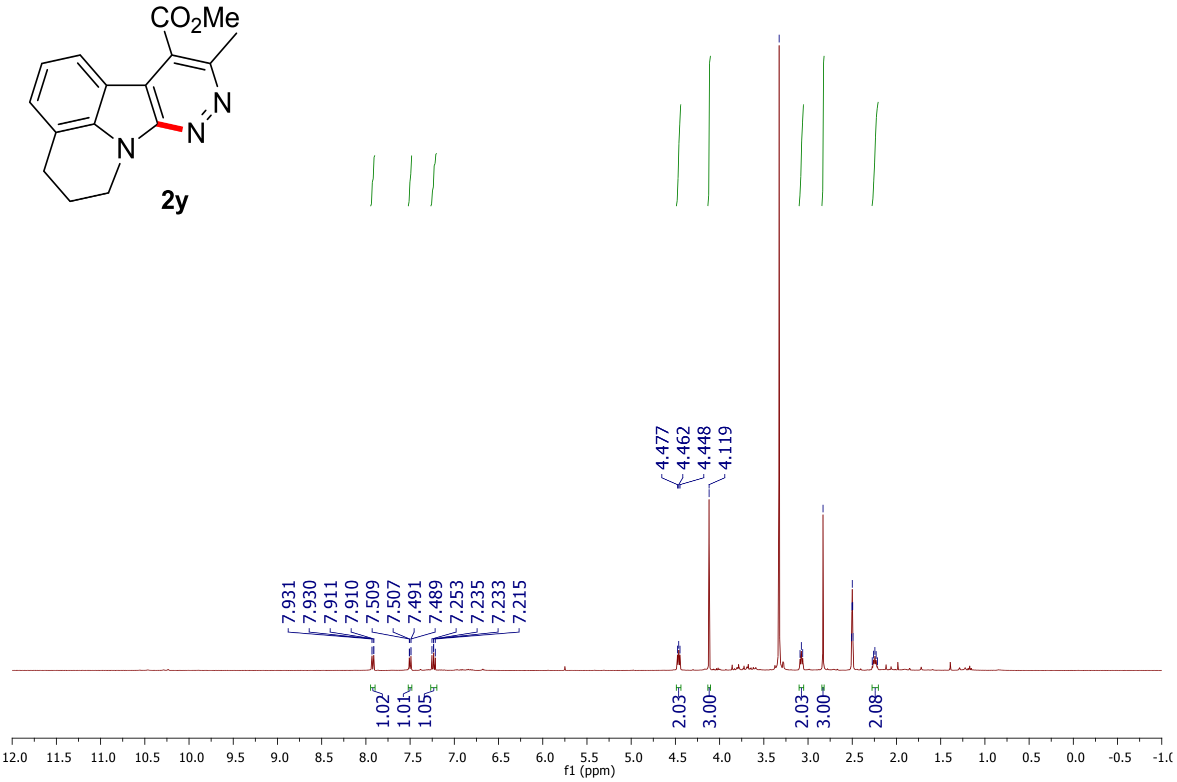

^{13}C{^{1}H} NMR of 2y (100 MHz, DMSO-d_6)

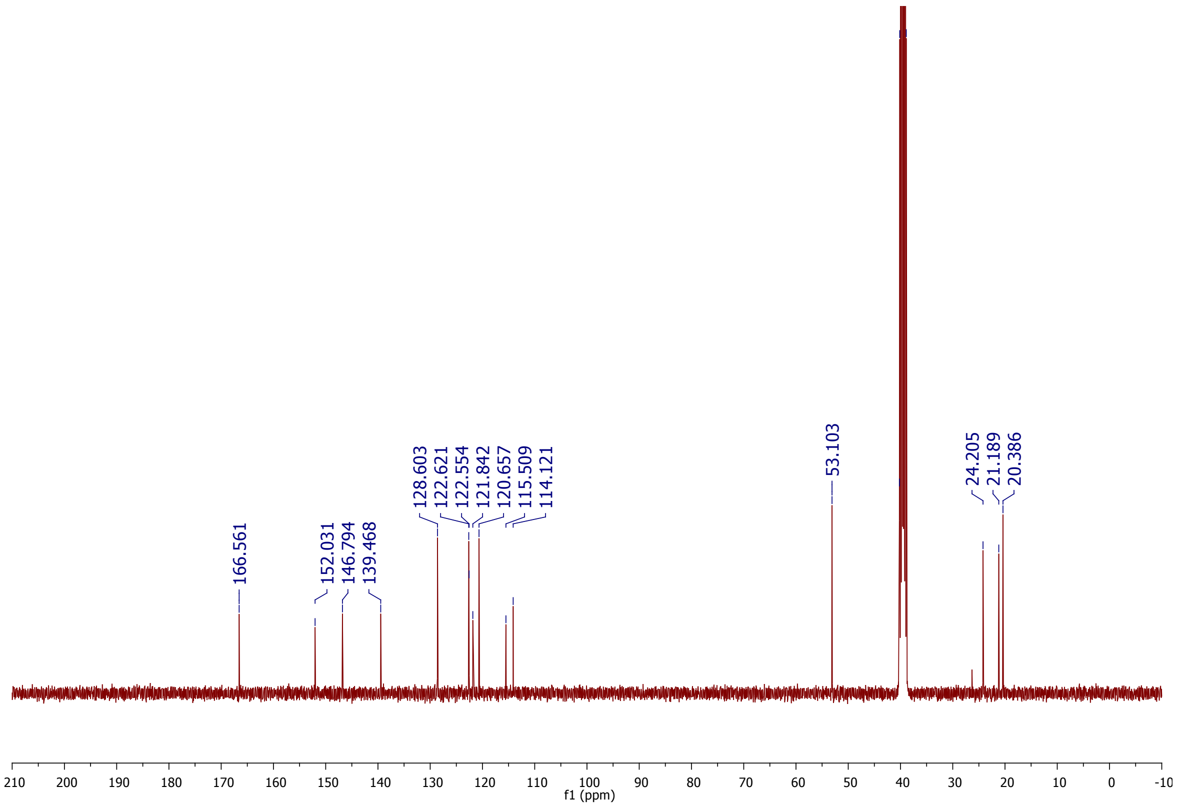

^{1}H NMR of intermediate C (400 MHz, DMSO-d_6)

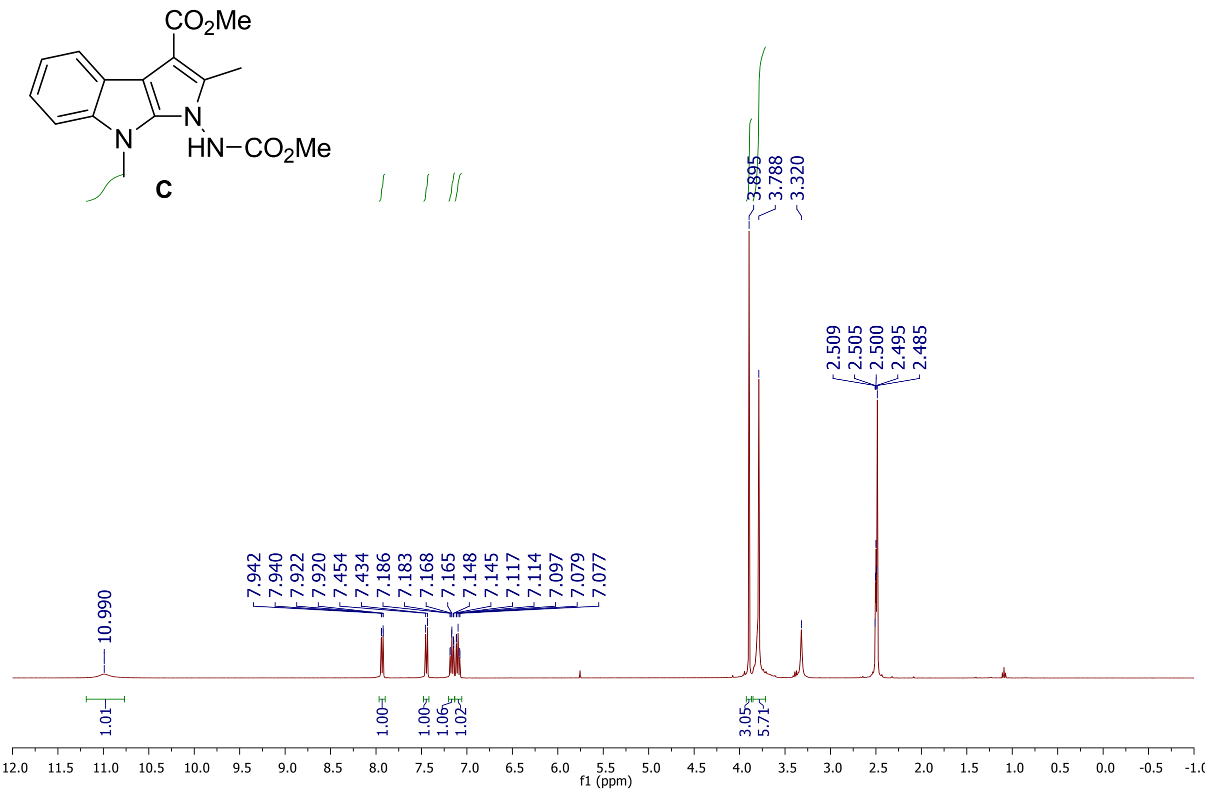

^{13}C{^{1}H} NMR of intermediate C (100 MHz, DMSO-d_6)

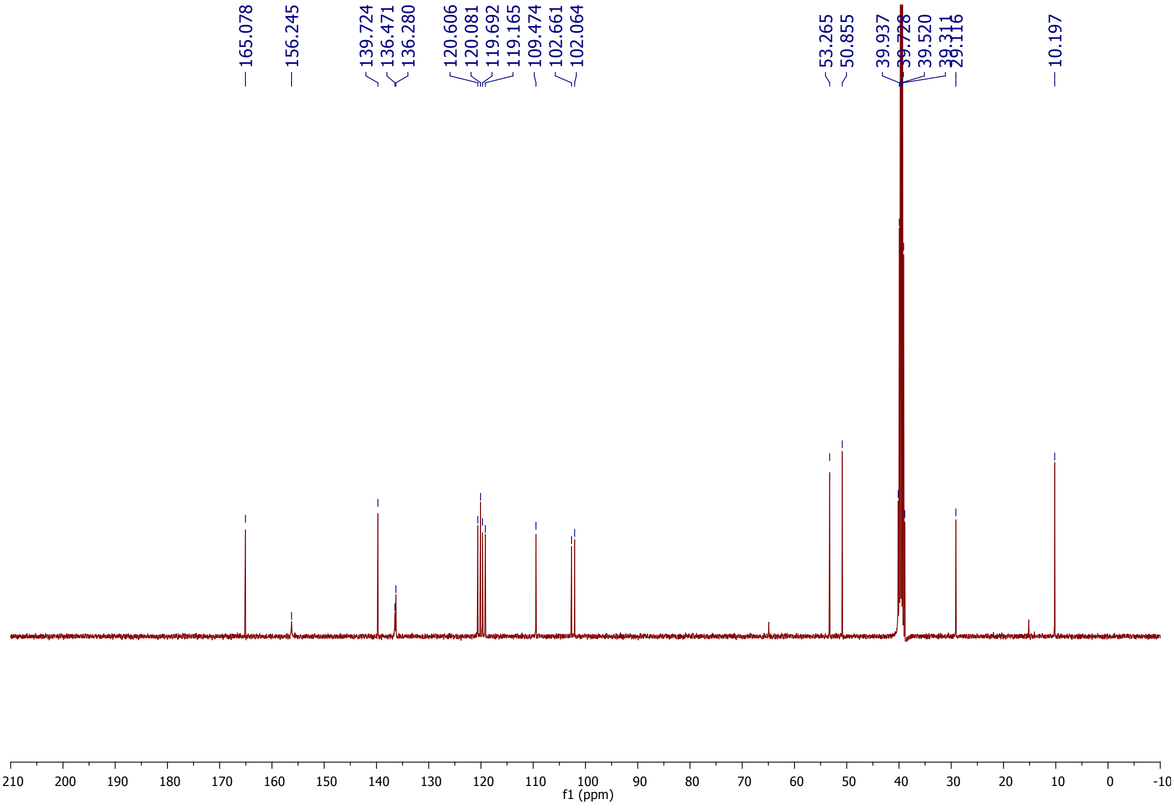

^{1}H NMR of D1 (400 MHz, DMSO-d_6)

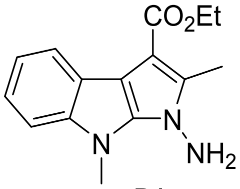

D1

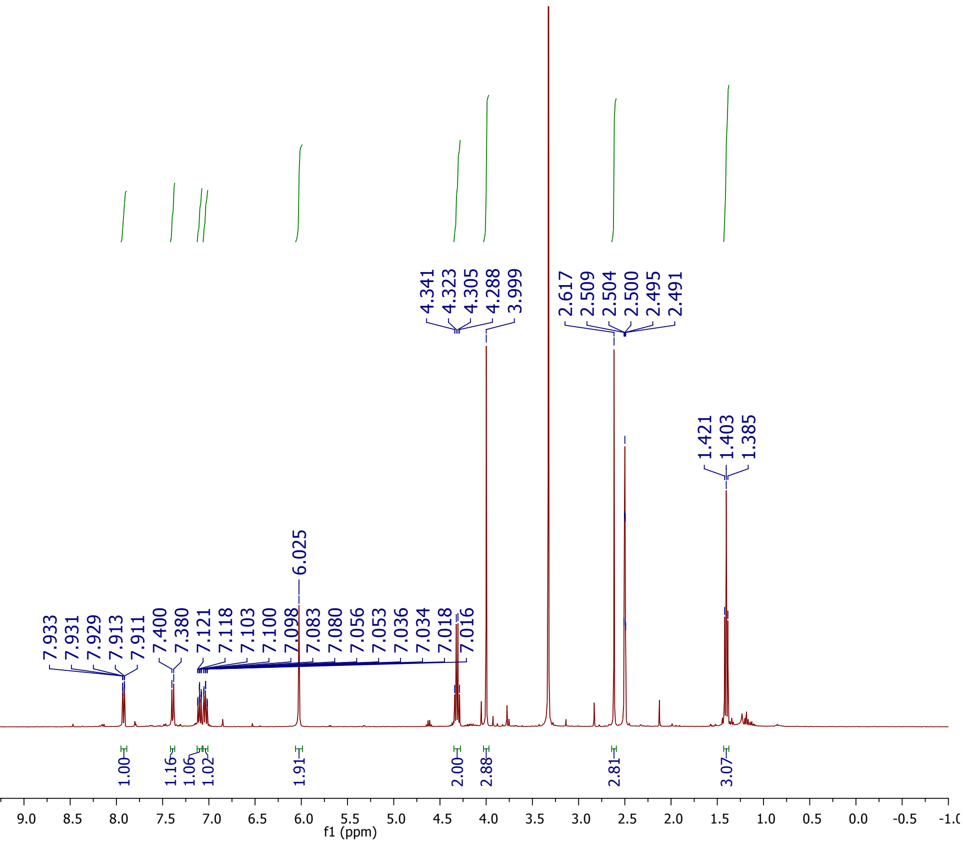

^{13}C{^{1}H} NMR of D1 (100 MHz, DMSO-d_6)

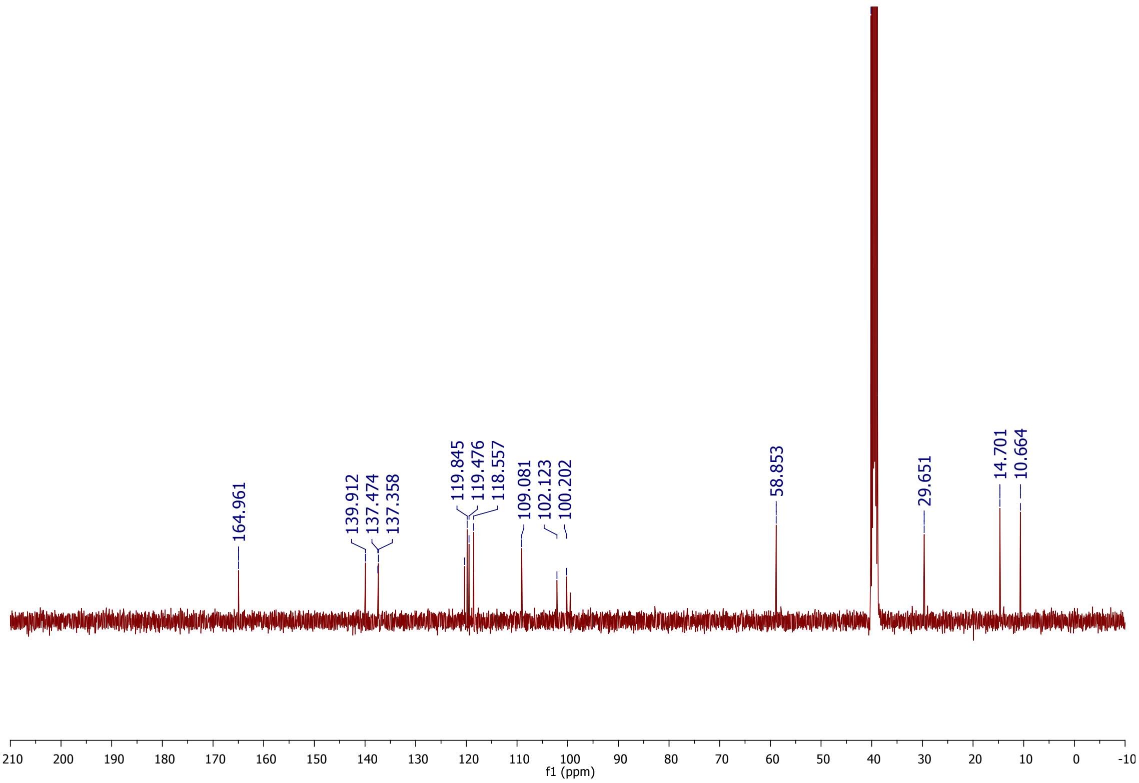

^{1}H NMR of 3 (400 MHz, DMSO-d_6)

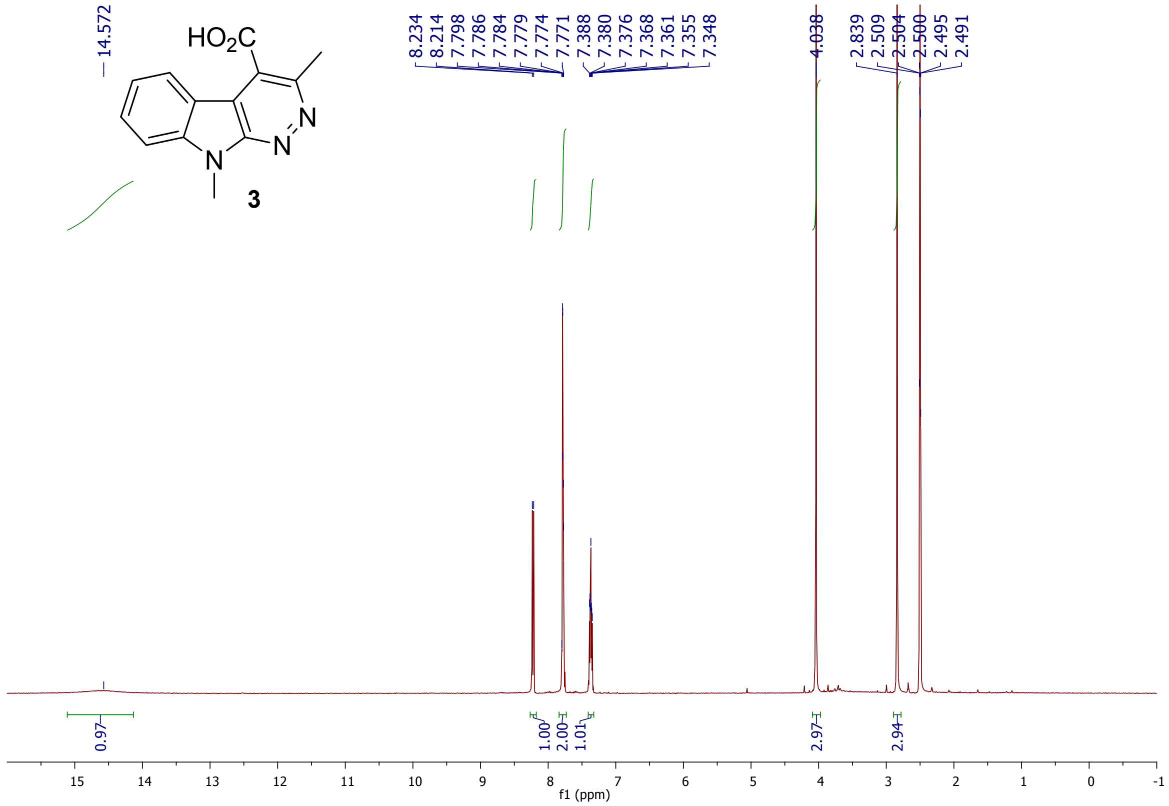

^{13}C{^{1}H} NMR of 3 (100 MHz, DMSO-d_6)

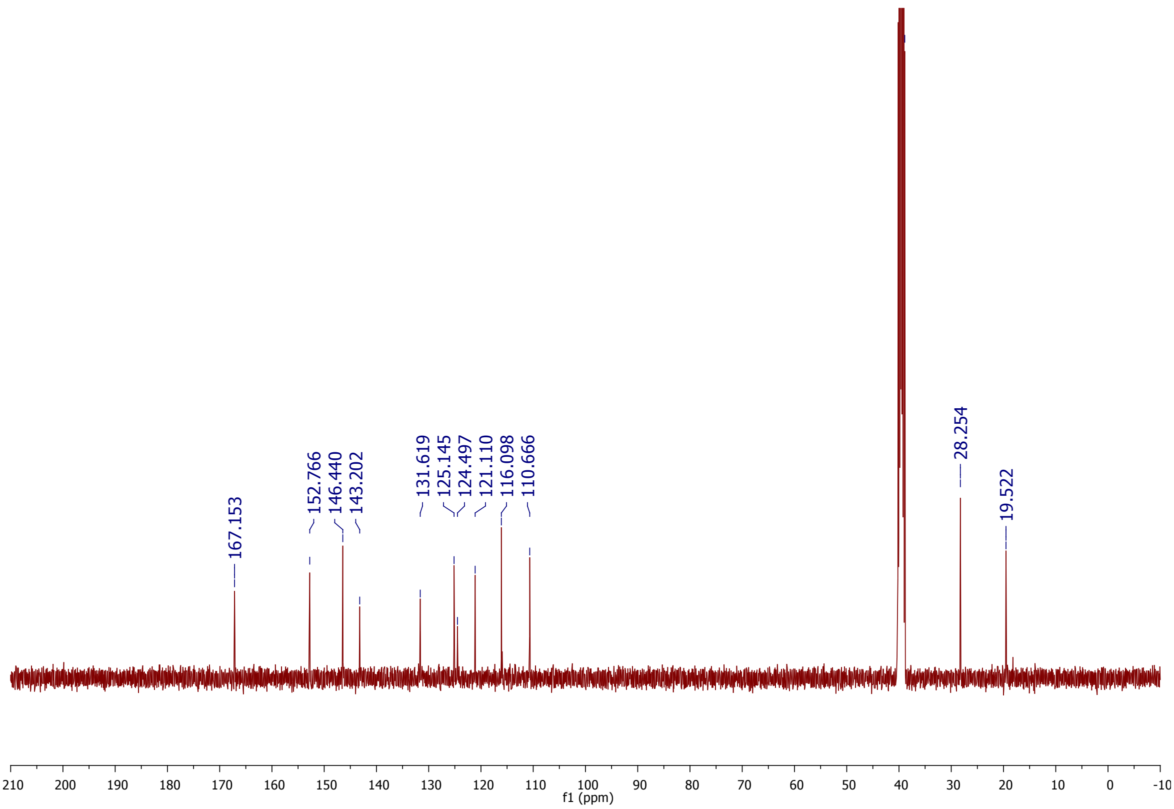

^{1}H NMR of 4 (400 MHz, DMSO-d_6)

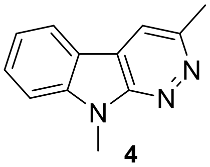

4

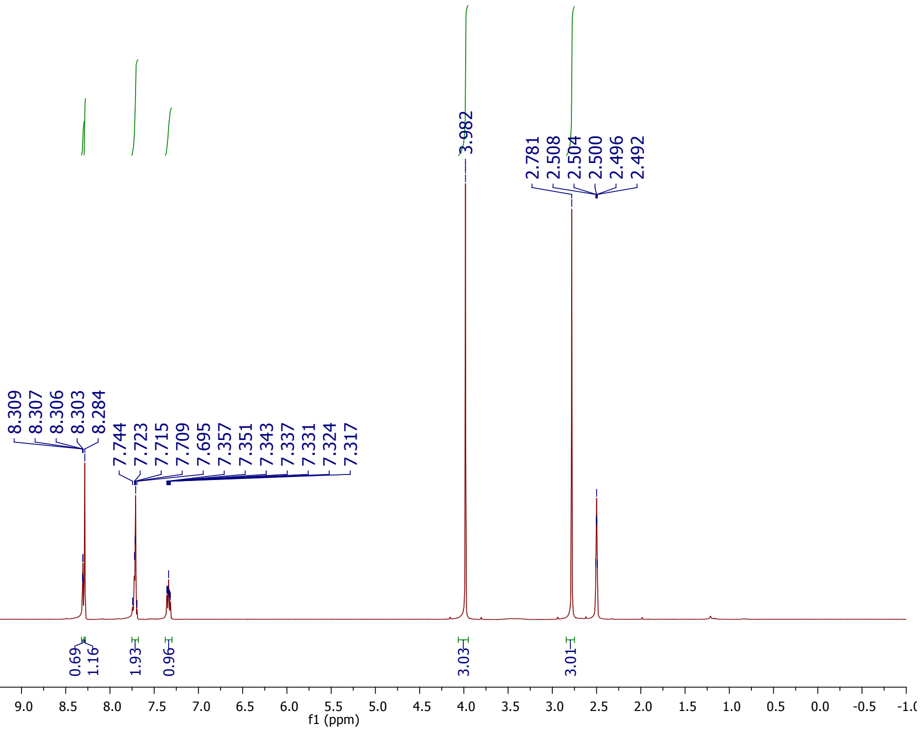

^{13}C{^{1}H} NMR of 4 (100 MHz, DMSO-d_6)

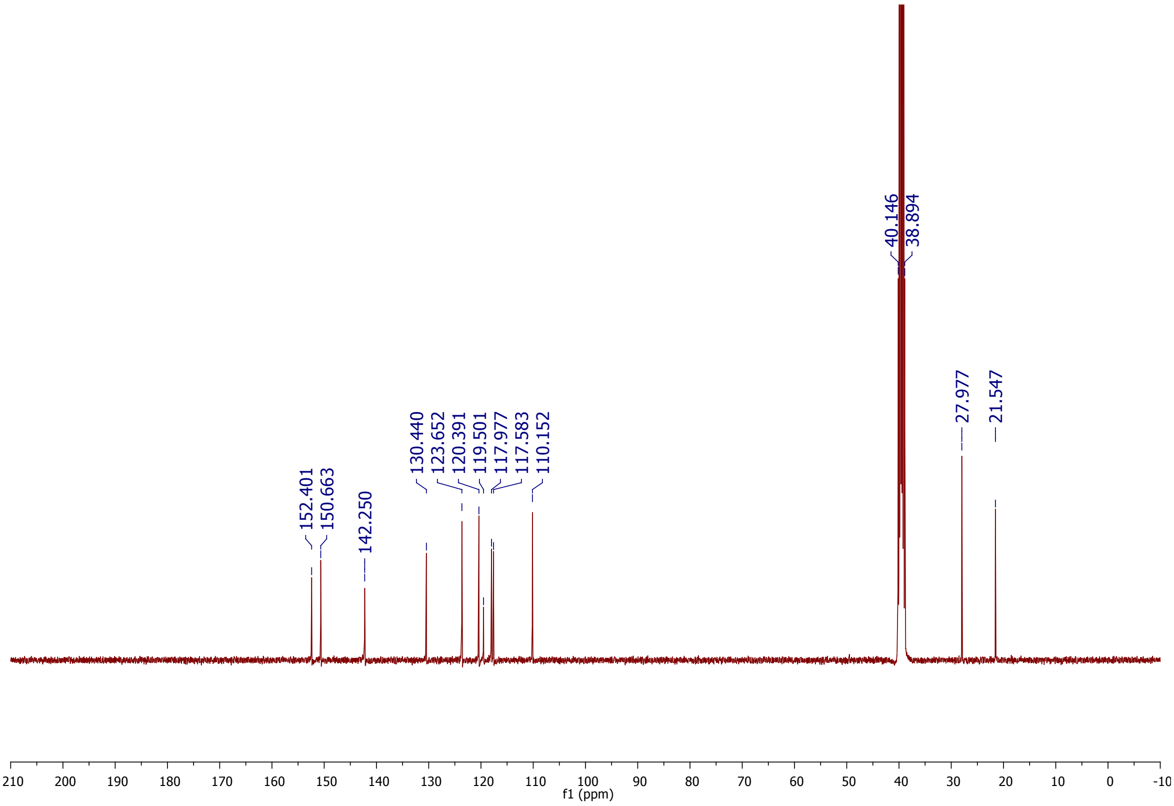

Supplement: Supplementary file 1 — jo1c02217_si_001.pdf [file jo1c02217_si_001.pdf]
